# Supplementary material for: Evidence to Underpin Vitamin A Requirements and Upper Limits in Children Aged 0 to 48 Months: A Scoping Review
Source: Nutrients. 2022 Jan 18;14(3):407. doi: 10.3390/nu14030407 (PMC8840537; doi:10.3390/nu14030407)
Supplement: Supplementary file 1 [file nutrients-14-00407-s001.zip › Vit A scoping review supp files 14Jan2022/nutrients-1494176-supplementary 14Jan2022.pdf]

Evidence to underpin vitamin A requirements and upper limits in children aged 0-48 months: a scoping review

Supplementary materials

Lee Hooper, Chizoba Esio-Bassey, Julii Brainard, Judith Fynn, Amy Jennings, Natalia Jones, Bhavesh Tailor, Asmaa Abdelhamid, Calvin Coe, Latife Esgunoglu, Ciara Fallon, Ernestina Gyamfi, Claire Hill, Stephanie Howard Wilsher, Nithin Narayanan, Titilopemi Oladosu, Ellice Parkinson, Emma Prentice, Meysoon Qurashi, Luke Read, Harriet Getley, Fujian Song, Ailsa Welch, Peter Aggett, Georg Lietz

This scoping review report is to be read in conjunction with its excel table (Vitamin A Tables - WHO Scoping excel 23 Dec 2021). The excel table tabulates the studies included within the review, providing brief details of each study, its bibliographic data and abstract (where available), and some further details as relevant.

Contents

Supplementary materials ... 1

Supplementary Table S1. Detailed inclusion criteria for the scoping review on vitamin A ... 2

Supplementary Table S2. Medline (Ovid) search strategy run 19th March 2021 ... 4

Supplementary Table S3. Embase (Ovid) search strategy run 19th March 2021 ... 6

Supplementary Table S4. CENTRAL and Cochrane Database of Systematic reviews search strategy run 19th March 2021 ... 8

Supplementary Text S5. Methodologies used in previous DRV development ... 10

Supplementary Table S6. References used in previous guidelines when setting Vitamin A requirements for infants and children ... 14

Supplementary Table S7. Dietary Reference Values and recommendations cited in existing guidelines. ... 79

Supplementary Table S8. Methodologies used in setting previous upper limits for vitamin A in infants and children ... 80

Supplementary Table S9. References relating to setting upper limits for vitamin A in infants and children ... 81

Supplementary References ... 88

Supplementary Table S1. Detailed inclusion criteria for the scoping review on vitamin A

Inclusion criteria: to be included a study must fit the inclusion criteria for population (part 1), question for vitamin A (part 2), AND study designs (part 3).

Area	Inclusion criteria	Exclusion criteria
1. Population of interest	• Generally healthy infants or children from birth to 9 years old (0 to 120 months)<br><br>**Healthy**: Generally healthy means that ≤20% of the population had a disease at baseline. Nutrition deficiencies of iron, magnesium and vitamin A can be included even at 100% (though not other nutritional deficiencies or malnutrition), as can children with obesity, underweight and overweight<br><br>**Age**: collect balance or toxicity studies where at least 1 child aged <10 years was included. Collect studies generally if mean age is likely to be <10. Exclude studies in adolescents, and where mean age is likely to be ≥10 years.	• Critically ill infants admitted to neonatal intensive care<br>• Feverish children as fever alters status markers<br>• Studies enrolling exclusively premature infants (≤32 weeks gestational age) or very low birth weight infants (≤1500 grams)<br>• Mothers during pregnancy or breastfeeding
2b. Questions of interest – vitamin A<br><br>Relevant terms: vitamin A comprises all-trans-retinol (called retinol) plus retinal, retinoic acid, retinyl esters, & provitamin A carotenoids such as beta-carotene (ß-carotene), *β*-cryptoxanthin and *α*-carotene. The biological value of substances with vitamin A activity is expressed as retinol equivalent (RE)*	• Vitamin A intake: quantified** oral intake of beta-carotene and/or Vitamin A (from breast milk, formula, foods, fortified and biofortified foods and supplements including assessments of exclusive or mixed breastfeeding duration, duration of formula use, weaning foods used) when related to outcomes such as status, growth, bioavailability, stores, losses, requirements, or growth/health outcomes<br>• Vitamin A bioavailability:<br>○ proportion of dietary beta-carotene or vitamin A absorbed (perhaps in relation to age, type of food, vitamin A status, other dietary components etc.)<br>○ Assessment of dietary factors affecting absorption/ bioavailability of beta-carotene or vitamin A<br>• Vitamin A stores: includes assessments of the quantity of vitamin A in the liver) and vitamin A stores at birth plus reports of new measurements that include calculation of stores<br>• Vitamin A status: assessed using total body store of retinol (i.e. as free retinol and retinyl esters) or, alternatively, as liver concentration of the vitamin.- or other markers of vitamin A status found useful in infants and children by BOND[1] such as dark adaptation or vit A in maternal umbilical cord blood when related to quantified intake or growth/ health outcomes<br>• Vitamin A losses: vitamin A lost through urine, in faeces via bile and to a lesser extent in breath (definitely include isotope dilution studies assessing losses, also include modelling studies, those on fractional catabolic rate, compartmental modelling, balance studies, excretion)<br>• Vitamin A requirements for growth: additional vitamin A needs to increase body size<br>• Health effects: assessment of relationships between quantified vitamin A intake (aged 0-9 years, including multifactorial interventions or exposures that include vitamin A intake) and any health or social outcome	• Multifactorial intervention studies where effects of vitamin A cannot be isolated from other non-nutritional interventions (smoking, lifestyle, medications etc.)<br>• Maternal health-related outcomes<br>• Any outcome measured only at birth in mothers or in infants (except for vitamin A stores assessed at any time point)<br>• Studies that only assess prevalence

	(including but not limited to night blindness, xerophthalmia, diarrhoea, infection mortality, all-cause mortality, infection rate, measures of growth– outcomes can be during childhood or later including<br>• Toxicity: Studies or reports (any design, even case studies) of health outcomes or toxicity associated with chronic or acute high beta-carotene or vitamin A intake, hyper-vitaminosis A or overload	
Comparators of interest	Any	None
3. Study designs of interest	• **Systematic reviews** (reviews including searching of at least 2 databases, predefined inclusion criteria and risk of bias assessment of included studies),<br>• **Intervention trials** with concurrent control groups (including individually and cluster randomised, crossover trials and non-RCTs), and before-after studies,<br>• **Mendelian randomization** studies,<br>• **Observational studies** including prospective cohort studies, retrospective cohort studies, nested case-control studies, other case-control studies, cross-sectional studies.<br>• Controlled or uncontrolled **absorption, excretion** (including using **isotope dilution** techniques) or **balance studies** (including chemical balance studies providing values for total vitamin A intake and losses).<br>• **Modelling** studies of iron, magnesium or vitamin A intake/status/health in infants or children<br>• **Toxicity** studies (assessing effects of high doses) are exceptions in that they can be ANY design including case studies and case series as well as dose-response studies<br>• Published **guidelines** of iron, magnesium or vitamin A requirements for infants or children (up to 9 years)<br>• **Protocols** (plans or trials registry entries) for relevant studies	• health-service utilization outcomes, cross-sectional studies reporting only prevalence data (no correlation or association analyses),<br>• case reports or case series except re toxicity,<br>• in vitro or cell-line studies,<br>• narrative (non-systematic) reviews,<br>• studies using non-concurrent cohorts or non-concurrent controls,<br>• studies in non-humans, animal studies

Footnotes:

* Vitamin A comprises all-*trans*-retinol (also called retinol) and the family of naturally occurring molecules associated with the biological activity of retinol (such as retinal, retinoic acid, retinyl esters), as well as provitamin A carotenoids that are dietary precursors of retinol. The biological value of substances with vitamin A activity is expressed as retinol equivalent (RE).

** oral dietary or supplemental intakes should be quantified. Where supplementary intakes are quantified dietary intakes do not need to be.

Supplementary Table S2. Medline (Ovid) search strategy run 19th March 2021

C1 - Database: Ovid MEDLINE(R) and Epub Ahead of Print, In-Process, In-Data-Review & Other Non-Indexed Citations, Daily and Versions(R) <1946 to March 18, 2021>

Search Strategy:

----

1 infan:.mp. or gestation:.tw. (1450804)
2 child, preschool/ or infant/ or infant, newborn/ (1596431)
3 p?ediatric$1.ti,ab. (355816)
4 (infant$1 or infancy or baby or babies or toddler* or neonate* or neo nate*).ti,ab. (529748)
5 (newborn$1 or new born$1 or pre school or preschool or kindergarten*).ti,ab. (205077)
6 1 or 2 or 3 or 4 or 5 (2160234)
7 animal/ not human/ (4768543)
8 6 not 7 (2061559)
9 exp Iron/ or exp Iron Compounds/ (154210)
10 (iron* or ferric or ferrous or fe2*).ti,ab. (219717)
11 9 or 10 (282209)
12 beta carotene/ or vitamin a/ (30259)
13 (retinol*or retinal* or cryptoxanthin* or caroten* or beta-caroten* or vitamin a or (retinyl adj ester*)).ab,ti. (55074)
14 12 or 13 (65225)
15 exp Magnesium/ or exp Magnesium Compounds/ (82220)
16 (magnesium* or mg2+ or img2+).ti,ab. (89428)
17 15 or 16 (131620)
18 11 or 14 or 17 (466541)
19 exp nutritional requirements/ or nutritional status/ (65432)
20 exp Eating/ or exp Dietary Supplements/ or breast feeding/ or exp infant nutritional physiological phenomena/ or diet/ or diet, vegetarian/ or diet, vegan/ or diet, healthy/ (359313)
21 (diet* or consum* or intake* or feed* or store or stores or TBI or food* or nutrition* or milk or wean* or eat or eating or eaten or formula* or breastmilk* or lactat* or food*).ti,ab. (2488706)
22 (need* or requir* or absorb* or overload* or absorption* or storage* or deficien* or metaboli* or status* or bioavailabilit* or adequat* or adequac* or concentration* or grow* or development* or homeostasis or transport*).ti,ab. (10658662)
23 exp Gastrointestinal Absorption/ (44731)
24 exp nutrition assessment/ (15617)
25 Iron/ae, df, me, to [Adverse Effects, Deficiency, Metabolism, Toxicity] (41655)
26 Magnesium/ae, me, to [Adverse Effects, Metabolism, Toxicity] (15065)
27 vitamin a/ae, df, me, to [Adverse Effects, Deficiency, Metabolism, Toxicity] (5574)
28 beta carotene/ae, df, me, to [Adverse Effects, Deficiency, Metabolism, Toxicity] (1378)
29 exp Biological Transport/ (306095)
30 Reference Values/ (161121)
31 Homeostasis/ (62995)
32 (balance stud* or isotope*).ti,ab,kf,kw. (81922)
33 ((composition* or measur*) adj3 tissue*).tw. (29463)
34 ((dietary or reference) adj3 (value* or dose* or level*)).tw. (43387)
35 exp Toxicity Tests/ (114104)
36 exp Ferritins/ or exp Receptors, Transferrin/ or Hepcidins/ (28426)
37 (ferritin* or hepcidin* or s-ferritin or (transferrin adj (receptor* or saturation*))).ti,ab. (39187)
38 hemoglobins/ or hemoglobin a/ (72178)
39 Dark Adaptation/ or Night Blindness/ (7264)
40 (total adj3 body).ti,ab. (40385)
41 19 or 20 or 21 or 22 or 23 or 24 or 25 or 26 or 27 or 28 or 29 or 30 or 31 or 32 or 33 or 34 or 35 or 36 or 37 or 38 or 39 or 40 (12197935)
42 18 and 41 (312507)

43 exp Anemia, Iron-Deficiency/ (10283)
44 ((anaemi* or anemi*) adj3 iron*).ti,ab. (13303)
45 Hypervitaminosis A/ (578)
46 exp Vitamin A Deficiency/ (5784)
47 exp Magnesium Deficiency/ (4302)
48 exp Iron, Dietary/ (2974)
49 42 or 43 or 44 or 45 or 46 or 47 or 48 (316965)
50 49 and 8 (21545)

****

Supplementary Table S3. Embase (Ovid) search strategy run 19th March 2021

Embase <1974 to 2021 March 18>

Search Strategy:

----

1 infan*.ti,ab. (519331)

2 exp infant/ or exp toddler/ (1021801)

3 gestation*.ti,ab. (300464)

4 (infant$1 or infancy or baby or babies or toddler* or neonate* or neo nate*).ti,ab. (634059)

5 (newborn$1 or new born$1 or pre school or preschool or kindergarten*).ti,ab. (240437)

6 1 or 2 or 3 or 4 or 5 (1521989)

7 animal/ not human/ (1106815)

8 6 not 7 (1491187)

9 exp iron/ (166018)

10 (iron* or ferric or ferrous or fe2*).ti,ab. (258217)

11 9 or 10 (313110)

12 retinal/ or retinol/ or alpha carotene/ or beta carotene/ or beta cryptoxanthin/ or cryptoxanthin/ (59072)

13 (retinol*or retinal* or cryptoxanthin* or caroten* or beta-caroten* or vitamin a or (retinyl adj ester*)).ab,ti. (59495)

14 12 or 13 (86986)

15 exp Magnesium/ (79203)

16 (magnesium* or mg2+ or img2+).ti,ab. (81169)

17 15 or 16 (121199)

18 11 or 14 or 17 (496585)

19 exp nutritional status/ or exp nutritional requirement/ (84789)

20 eating/ or dietary supplement/ or exp infant feeding/ (113730)

21 experimental diet/ or healthy diet/ or unhealthy diet/ or vegetarian diet/ or western diet/ (13610)

22 (diet* or consum* or intake* or feed* or store or stores or TBI or food* or nutrition* or milk or wean* or eat or eating or eaten or formula* or breastmilk* or lactat*).ti,ab. (3132935)

23 (requir* or absorb* or overload* or absorption* or storage* or deficien* or metaboli* or status* or bioavailabilit* or adequat* or adequac* or concentration* or grow* or development* or homeostasis or transport*).ti,ab. (11954558)

24 gastrointestinal absorption/ or exp intestine absorption/ (35913)

25 nutritional assessment/ (30939)

26 iron/to, si, th [Drug Toxicity, Side Effect, Therapy] (2921)

27 magnesium/ae, to [Adverse Drug Reaction, Drug Toxicity] (1015)

28 retinol/ae, to [Adverse Drug Reaction, Drug Toxicity] (1491)

29 beta carotene/ae, to [Adverse Drug Reaction, Drug Toxicity] (499)

30 active transport/ or iron transport/ or transport kinetics/ (32145)

31 exp reference value/ (135858)

32 homeostasis/ or iron homeostasis/ (123043)

33 [(balance stud* or isotope*).ti,ab,kf,kw.] (0)

34 ((composition* or measur*) adj3 tissue*).tw. (38945)

35 ((dietary or reference) adj3 (value* or dose* or level*)).tw. (57872)

36 exp toxicity/ or toxicity testing/ (736670)

37 iron binding protein/ or ferritin/ or hemosiderin/ or transferrin/ (76632)

38 (ferritin* or hepcidin* or s-ferritin or (transferrin adj (receptor* or saturation*))).ti,ab. (57622)

39 hemoglobins/ or hemoglobin a/ (161125)

40 (total adj3 body).ti,ab. (54886)

41 19 or 20 or 21 or 22 or 23 or 24 or 25 or 26 or 27 or 28 or 29 or 30 or 31 or 32 or 33 or 34 or 35 or 36 or 37 or 38 or 39 or 40 (14023001)

42 18 and 41 (349144)

43 iron deficiency anemia/ (26350)

44 retinol intoxication/ (595)

45 exp retinol deficiency/ (6368)

46 exp Magnesium Deficiency/ (13111)

47 42 or 43 or 44 or 45 or 46 (366264)

48 47 and 8 (22942)

Supplementary Table S4. CENTRAL and Cochrane Database of Systematic reviews search strategy run 19th March 2021

Database: Cochrane Database of Systematic Reviews & CENTRAL
Search Name: Infants search for WHO 21Mar2021 run
Run: 21/03/2021 21:57:05

ID Search
#1 MeSH descriptor: [Child, Preschool] explode all trees
#2 (infant* or infancy):ti,ab,kw (Word variations have been searched)
#3 MeSH descriptor: [Infant] explode all trees
#4 p?ediatric*:ti,ab
#5 (infant or infants or infancy or baby or babies or toddler* or neonate or neonates):ti,ab
#6 (newborn or newborns or preschool* or pre-school* or kindergarten*):ti,ab
#7 #1 or #2 or #3 or #4 or #5 or #6
#8 MeSH descriptor: [Iron] explode all trees
#9 (iron* or ferric or ferrous or fe2):ti,ab
#10 MeSH descriptor: [Iron Compounds] explode all trees
#11 #8 or #9 or #10
#12 MeSH descriptor: [Vitamin A] explode all trees
#13 MeSH descriptor: [Carotenoids] explode all trees
#14 (retinol* or retinal* or cyptoxanthin* or caroten* or beta-caroten* or (retinyl next ester*) or (vitamin A)):ti,ab
#15 #12 or #13 or #14
#16 MeSH descriptor: [Magnesium] explode all trees
#17 MeSH descriptor: [Magnesium Compounds] explode all trees
#18 (magnesium*):ti,ab
#19 #16 or #17 or #18
#20 #11 or #15 or #19
#21 MeSH descriptor: [Nutritional Requirements] explode all trees
#22 MeSH descriptor: [Nutritional Status] explode all trees
#23 MeSH descriptor: [Gastrointestinal Absorption] explode all trees
#24 MeSH descriptor: [Breast Feeding] explode all trees
#25 MeSH descriptor: [Bottle Feeding] explode all trees
#26 MeSH descriptor: [Infant Nutritional Physiological Phenomena] explode all trees
#27 MeSH descriptor: [Eating] explode all trees
#28 MeSH descriptor: [Dietary Supplements] explode all trees
#29 MeSH descriptor: [Diet] this term only
#30 MeSH descriptor: [Diet, Healthy] explode all trees
#31 (diet* or consum* or intake* or feed* or store or stores or TBI or food* or nutrition* or milk or wean* or eat or eating or eaten or formula* or breastmilk* or lactat* or food*):ti,ab
#32 (need* or requir* or absorb* or overload* or absorption* or storage* or deficien* or metaboli* or status* or bioavailabilit* or adequat* or adequac* or concentration* or grow* or development* or homeostasis or transport*):ti,ab
#33 MeSH descriptor: [Nutrition Assessment] explode all trees
#34 MeSH descriptor: [Iron] explode all trees and with qualifier(s): [adverse effects - AE, toxicity - TO, deficiency - DF, metabolism - ME]
#35 MeSH descriptor: [Magnesium] explode all trees and with qualifier(s): [metabolism - ME, toxicity - TO, adverse effects - AE]
#36 MeSH descriptor: [Vitamin A] explode all trees and with qualifier(s): [metabolism - ME, toxicity - TO, adverse effects - AE]
#37 MeSH descriptor: [beta Carotene] explode all trees and with qualifier(s): [deficiency - DF, metabolism - ME, toxicity - TO, adverse effects - AE]
#38 MeSH descriptor: [Nutrition Assessment] explode all trees
#39 MeSH descriptor: [Biological Transport] explode all trees
#40 MeSH descriptor: [Reference Values] explode all trees
#41 MeSH descriptor: [Homeostasis] explode all trees

#42 (balance stud* or isotope*):ti,ab
#43 ((composition* or measur*) near tissue*):ti,ab
#44 ((dietary or reference) near (value* or dose* or level*)):ti,ab
#45 MeSH descriptor: [Toxicity Tests] explode all trees
#46 MeSH descriptor: [Ferritins] explode all trees
#47 MeSH descriptor: [Receptors, Transferrin] explode all trees
#48 MeSH descriptor: [Hepcidins] explode all trees
#49 MeSH descriptor: [Hemoglobins] this term only
#50 MeSH descriptor: [Hemoglobin A] this term only
#51 (ferritin* or hepcidin* or s-ferritin or (transferrin adj (receptor* or saturation*))):ti,ab
#52 (total next body):ti,ab
#53 MeSH descriptor: [Dark Adaptation] explode all trees
#54 MeSH descriptor: [Night Blindness] explode all trees
#55 #21 or #22 or #23 or #24 or #25 or #26 or #27 or #28 or #29 or #30
#56 #31 or #32 or #33 or #34 or #35 or #36 or #37 or #38 or #39 or #40
#57 #41 or #42 or #43 or #44 or #45 or #46 or #47 or #48 or #49 or #50
#58 #51 or #52 or #53 or #54 or #55 or #56 or #57
#59 #20 and #58
#60 MeSH descriptor: [Anemia, Iron-Deficiency] explode all trees
#61 ((anaemi* or anemi*) near iron*):ti,ab
#62 MeSH descriptor: [Hypervitaminosis A] explode all trees
#63 MeSH descriptor: [Vitamin A Deficiency] explode all trees
#64 MeSH descriptor: [Magnesium Deficiency] explode all trees
#65 MeSH descriptor: [Iron, Dietary] explode all trees
#66 #59 or #60 or #61 or #62 or #63 or #64 or #65
#67 #7 and #66

Supplementary Text S5. Methodologies used in previous DRV development

Methods text directly quoted from existing guidelines appear below. The texts are copied directly from the methodology sections of earlier guidelines, giving insight into how previous DRVs were set for infants and children.

Scientific Opinion on Dietary Reference Values for vitamin A EFSA Journal 2015;13(3):4028 11[2]

Breast milk retinol concentration is influenced by maternal vitamin A status, and large variation in mean total retinol concentration of mature breast milk is observed (Section 2.3.6.3 [editors note: apologies we have not been able to retain all of these sections and links]). Using estimated retinol intakes in exclusively breast-fed infants as a basis for setting a DRV for infants aged 7–11 months is therefore associated with considerable uncertainty. Thus, the Panel considers it more appropriate to derive DRVs for infants aged 7–11 months on the same basis as for adults.

For infants aged 7–11 months and children, the average intake of vitamin A required to maintain a concentration of 20 µg retinol/g liver is calculated with the same equation as for adults but with values for reference body weight and liver/body weight ratio specific to infants and children (Section 5.1.3.1). Although there is some indication that the fractional catabolic rate of retinol may be higher in children than in adults, data are limited (Section 2.3.6.1). In the absence of more robust data, the Panel decides to apply the value for fractional catabolic rate observed in adults and to correct it on the basis of a growth factor (Section 5.1.3.1).

This approach is preferred to scaling down from adults based on body weight (either isometric or allometric), as retinol is mainly stored in the liver, the size of which does not linearly change with body weight during growth, and as vitamin A requirement is not directly related to energy needs and expenditure.

The requirement to maintain a concentration of 20 µg retinol/g liver can be calculated in infants and children on the basis of the factorial approach as follows:

AR (µg RE/day) = target liver concentration (µg retinol/g) × body/liver retinol stores ratio × liver/body weight ratio (%) × fractional catabolic rate of retinol (%) × (1/efficiency of body storage (%)) × reference body weight (kg) × (1 + growth factor) × 103

The Panel uses the following values for infants aged 7–11 months and children (Section 5.1.3.1): (1) a total body/liver retinol stores ratio of 1.25 (i.e. 80 % of retinol in the body is stored in the liver); (2) an age-specific liver/body weight ratio; (3) a fractional catabolic rate of retinol of 0.7 % per day; (4) an efficiency of storage in the whole body of ingested retinol of 50 %; and (5) a growth factor of 0.57 for infants aged 7–11 months, 0.25 for boys and girls aged 1–3 years, 0.06 for boys and girls aged 4–6 years, 0.13 for boys and girls aged 7–10 years, 0.11 for boys and 0.08 for girls aged 11–14 years, and 0.08 for boys and 0.03 for girls aged 15–17 years (EFSA NDA Panel, 2014a).

It was considered unnecessary to give sex-specific values for infants and children up to 14 years of age. As for adults, a CV of 15 % is used for setting PRIs for the respective age groups (Table 6). PRIs were rounded to the closest 50 or 100.

Vitamin and mineral requirements in human nutrition. 2004: World Health Organization[3]

Vitamin A
2.6 The mean requirement for an individual is defined as the minimum daily intake of vitamin A, expressed as µg retinol equivalents (µg RE), to prevent xerophthalmia in the absence of clinical or subclinical infection. This intake

should account for the proportionate bioavailability of preformed vitamin A (about 90%) and provitamin A carotenoids from a diet that contains sufficient fat (e.g. at least 10 g daily). The required level of intake is set to prevent clinical signs of deficiency, allow for normal growth, and reduce the risk of vitamin A-related severe morbidity and mortality within any given population. It does not allow for frequent or prolonged periods of infections or other stresses.

The safe level of intake for an individual is defined as the average continuing intake of vitamin A required to permit adequate growth and other vitamin A-dependent functions and to maintain an acceptable total body reserve of the vitamin. This reserve helps offset periods of low intake or increased need resulting from infections and other stresses. Useful indicators include a plasma retinol concentration above 0.70mmol/l, which is associated with a relative dose response below 20%, or a modified relative dose response below 0.06. For lactating women, breast-milk retinol levels above 1.05mmol/l (or above 8µg/g milk fat) are considered to reflect minimal maternal stores because levels above 1.05mmol/l are common in populations known to be healthy and without evidence of insufficient dietary vitamin A.

2.6.1. Vitamin A requirements for infants are calculated from the vitamin A provided in human milk. During at least the first 6 months of life, exclusive breastfeeding can provide sufficient vitamin A to maintain health, permit normal growth, and maintain sufficient stores in the liver (104). Reported retinol concentrations in human milk vary widely from country to country (0.70–2.45mmol/l). In some developing countries, the vitamin A intake of breast-fed infants who grow well and do not show signs of deficiency ranges from 120 to 170µg RE/day (25, 104). Such intakes are considered adequate to cover infant requirements if the infant's weight is assumed to be at least at the 10th percentile according to WHO standards (62). However, this intake is unlikely to build adequate body stores, given that xerophthalmia is common in preschool-age children in the same communities with somewhat lower intakes. Because of the need for vitamin A to support the growth rate of infancy, which can vary considerably, a requirement estimate of 180µg RE/day seems appropriate. The safe level for infants up to 6 months of age is based on observations of breast-fed infants in communities in which good nutrition is the norm. Average consumption of human milk by such infants is about 750ml/day during the first 6 months (104). Assuming an average concentration of vitamin A in human milk of about 1.75mmol/l, the mean daily intake would be about 375µg RE, which is therefore the recommended safe level. From 7–12 months, human milk intake averages 650ml/day, which would provide 325µg of vitamin A daily. Because breast-fed infants in endemic vitamin A-deficient populations are at increased risk of death from 6 months onward, the requirement and recommended safe intake levels are increased to 190µg RE/day and 400µg RE/day, respectively. The requirement (with allowance for variability) and the recommended safe intake for older children may be estimated from those derived for late infancy (i.e. 20 and 39µg RE/kg body weight/day) (62). On this basis, and including allowances for storage requirements and variability, requirements for preschool-age children would be in the range of 200–400µg RE daily. In poor communities where children 1–6 years old are reported to have intakes of about 100–200µg RE/day, signs of VAD do occur; in southern India these signs were relieved and risk of mortality was reduced when the equivalent of 350–400µg RE/day was given to children weekly (105). In the United States, most preschool-age children maintain serum retinol levels of 0.70mmol/l or higher while consuming diets providing 300–400µg RE/day (from the databank for the third National Health and Nutrition Examination Survey [http://www.cdc.gov/nchs/nhanes.htm]).

Dietary Reference Intakes for Vitamin A, Vitamin K, Arsenic, Boron, Chromium, Copper, Iodine, Iron, Manganese, Molybdenum, Nickel, Silicon, Vanadium, and Zinc[4]

Vitamin A
No functional criteria of vitamin A status have been demonstrated that reflect response to dietary intake in infants. Thus, recommended intakes of vitamin A are based on an Adequate Intake (AI) that reflects a calculated mean vitamin A intake of infants principally fed human milk.

Ages 0 through 6 Months. Using the method described in Chapter 2, the AI of vitamin A for infants ages 0 through 6 months is based on the average amount of vitamin A in human milk that is consumed. After rounding, an AI of 400 µg retinol activity equivalents (RAE)/day is set based on the average volume of milk intake of 0.78 L/day (see Chapter 2) and an average concentration of vitamin A in human milk of 1.70 µmol/L (485 µg/L) during the first 6 months of lactation (Canfield et al., 1997, 1998) (see Table 4-5). Because the bioconversion of carotenoids in milk and in infants is not known, the contribution of carotenoids in human milk to meeting the vitamin A requirement of infants was not considered.

Ages 7 through 12 Months. Using the method described in Chapter 2 to extrapolate from the AI for infants ages 0 through 6 months fed human milk, the intake from human milk for the older infants is 483 µg RAE/day of vitamin A.

The vitamin A intake for older infants can also be determined by estimating the intake from human milk (concentration × 0.6 L/ day) and complementary foods (Chapter 2). Vitamin A intake data (n = 45) from complementary foods was estimated to be 244 µg/day based on data from the Third National Health and Nutrition Examination Survey. The average intake from human milk is approximately 291 µg/day (485 µg/L × 0.6 L/day). Thus, the total vitamin A intake is estimated to be 535 µg RAE/day (244 µg/day + 291 µg/day).

On the basis of these two approaches and rounding, the AI was set at 500 µg RAE/day. The AI for infants is greater than the Recommended Dietary Allowance (RDA) for young children because the RDA is based on extrapolation of adult data (see “Children and Adolescents Ages 1 through 18 Years”).

Children and Adolescents Ages 1 through 18 Years: No data are available to estimate an average requirement for children and adolescents. A computational method is used that includes an allowance for adequate liver vitamin A stores to set the Estimated Average Requirement (EAR) (see “Adults Ages 19 Years and Older”). The EAR for children and adolescents is extrapolated from adults by using metabolic body weight and the method described in Chapter 2. If total body weight is used, the RDA for children 1 through 3 years would be 200 µg RAE/day. If metabolic weight (kg0.75) is used, the RDA would be 300 µg RAE/day. Studies conducted in developing countries indicate that xerophthalmia and serum retinol concentrations of less than 20 µg/dL exist among preschool children with daily intakes of up to 200 µg of vitamin A, whereas 300 µg/day of vitamin A is associated with serum retinol concentrations greater than 30 µg/dL (Reddy, 1985). Although similar data are lacking in developed countries, to ensure that the RDA will meet the requirement of almost all North American preschool children, metabolic weight was used to extrapolate from adults.

The RDA for vitamin A is set by using a coefficient of variation (CV) of 20 percent based on the calculated half-life values for liver vitamin A (see “Adults Ages 19 Years and Older”). The RDA is defined as equal to the EAR plus twice the CV to cover the needs of 97 to 98 percent of individuals in the group (therefore, for vitamin A the RDA is 140 percent of the EAR). The calculated values for the RDAs have been rounded to the nearest 100 µg.

Tolerable upper intake limits
Data Selection. Case reports of hypervitaminosis A in infants were used to identify a LOAEL and derive a UL. Data were not available to identify a NOAEL.

Identification of a LOAEL. A LOAEL of 6,460 µg/day of vitamin A (which was rounded to 6,000 µg/day) was identified by averaging the lowest doses of four case reports (Persson et al., 1965). Four cases of hypervitaminosis A occurred after doses of 5,500 to 6,750 µg/day of preformed vitamin A for 1 to 3 months (Table 4-10). The age of onset of symptoms ranged from 2.5 to 5.5 months and included anorexia, hyperirritability, occipital edema, pronounced craniotabes, bulging fontanels, increased intracranial pressure, and skin lesions and desquamation. The lowest dose associated with a bulging fontanel involved a 4-month-old girl given a daily dose of 24 drops of AD-vimin (about 5,500 µg of vitamin A) for 3 months. Her fontanels bulged 0.5 centimetres above the plane of the skull. The other three cases involved a dose of 6,750 µg/day of vitamin A for 1 to 2.5 months. Increased intracranial pressure and bulging fontanels were observed in these cases as well. Other effects observed at the higher dose included anorexia, hyperirritability, occipital edema, pronounced craniotabes, skin lesions, skin desquamation, epiphyseal line changes, and cortical hyperostosis on x-rays.

Uncertainty Assessment. A UF of 10 was selected to account for the uncertainty of extrapolating a LOAEL to a NOAEL for a non-severe and reversible effect (i.e., bulging fontanel) and the inter-individual variability in sensitivity.

Derivation of a UL. The LOAEL of 6,000 µg/day was divided by a UF of 10 to calculate a UL of 600 µg/day of preformed vitamin A for infants.

Children and Adolescent Boys. There are limited case report data of hypervitaminosis A (e.g., bulging anterior fontanels, increased intracranial pressure, hair loss, increased suture markings on the skull, and periosteal new bone formation) in children and adolescents after doses ranging from 7,000 µg/day in young children to 15,000 µg/day in older children and adolescents (Farris and Erdman, 1982; Siegel and Spackman, 1972; Smith and Goodman, 1976). Given the dearth of information and the need for conservativism, the UL values for children and adolescents are extrapolated from those established for adults. Thus, the adult UL of 3,000 µg/day of preformed vitamin A was adjusted for children and adolescents on the basis of relative body weight as described in Chapter 2 with use of reference weights from Chapter 1 (Table 1-1). Values have been rounded.

Dietary Reference Values for Food Energy and Nutrients for the United Kingdom 1991[5]

Vitamin A

RDAs for infants have usually been based on the vitamin A provided by breast milk. Breast fed infants do not show signs of vitamin A deficiency even on intakes little above 100 µg/d[8'9], although these would probably not be enough to maintain satisfactory reserves. The recent FAO/ WHO Expert Group considered that a daily intake of 350 µg retinol equivalents would meet the needs of all healthy infants and allow the building and maintaining of sufficient liver stores[6], and the Panel has accepted this value as the RNI. If the coefficient of variation is assumed to be 20 per cent as in adults, the rounded value for the EAR becomes 250 µg/d and for the LRNI 150 µg/d.

Children have a requirement for vitamin A for growth, in addition to the requirement (as in adults) to compensate for the loss of body stores. In the absence of data on which to set DRVs, the Panel have interpolated from the values for infants up to the adult values (Table 8.1). Intakes lower than those suggested here maintained satisfactory plasma retinol concentrations in preschool children in India.

Supplementary Table S6. References used in previous guidelines when setting Vitamin A requirements for infants and children

Guideline - title	Guideline – linking section	Guideline – linking text	Study - title and Endnote link	Study - abstract	Study - ref	Study - type
Scientific Opinion on Dietary Reference Values for vitamin A EFSA Journal 2015;13(3):4028 11	Health consequences of deficiency	Night blindness, the first ocular symptom of deficiency, responds rapidly to an increase in vitamin A intake	Impact of vitamin A supplementation on prevalence and incidence of xerophthalmia in Nepal [6]	**Purpose:** To assess the impact of vitamin A supplementation at 4-month intervals on the prevalence and incidence of xerophthalmia among preschool-age children. **Methods:** A stratified, random sample of 40 wards with 4766 children in Sarlahi district of Nepal was selected to participate in a randomized, controlled, community trial. In the vitamin A group, at 4-month intervals, neonates received 50,000 IU, 1- to 11-month-old infants received 100,000 IU, and children 1 through 4 years of age received 200,000 IU. Children underwent eye examination before the intervention and 16 months later. Results: Before the intervention, 4318 children were examined for xerophthalmia. The prevalence was 2.3% in the vitamin A group and 3.3% in the placebo group. All children with xerophthalmia were treated with vitamin A at the time of the examination. Of those examined at baseline, 38 in the vitamin A group and 48 in the placebo group died in the 16 months after intervention. There were 1871 (84%) surviving children in the vitamin A group and 1711 (85%) in the placebo group examined at follow-up. After adjustment for the baseline prevalence of xerophthalmia, vitamin A reduced the prevalence at follow-up by 63% (95% confidence interval, 21% to 83%). The apparent incidence was 3.2/1000 per year in the vitamin A group and 9.2/1000 per year in the placebo group, an adjusted reduction of 62% (95% confidence interval, 0% to 86%).<br>Conclusions: Supplementation was effective at reducing the prevalence and incidence of xerophthalmia.	2	RCT
Dietary Reference Intakes for Vitamin A, Vitamin K, Arsenic, Boron, Chromium, Copper, Iodine, Iron, Manganese, Molybdenum, Nickel, Silicon, Vanadium, and Zinc	Clinical Effects of Inadequate Intake	High-dose (60 mg) vitamin A supplementation reduced the incidence of night blindness by 63 percent in Nepalese children				
Scientific Opinion on Dietary Reference Values for vitamin A EFSA Journal 2015;13(3):4028 11	Health consequences of deficiency	Vitamin A deficiency also induces follicular hyperkeratosis, which disappears after retinol or β-carotene supplementation	Nutritional Status of Preschool Mexican-American Migrant Farm Children [7]	The nutritional and medical problems of 300 Mexican-American preschool children of migrant workers were evaluated in the spring of 1969 in Colorado. Outstanding in the history was the high infant mortality of 63 deaths per 1,000 live births. Frequent findings on physical examination included low height attainment, upper-respiratory tract infections, skin infections, dental caries, hypertrophied tongue papillae, and conjunctival folliculitis. Biochemical testing showed low vitamin A levels in 159 children, low alkaline phosphatase levels in 120 children, and low total serum proteins in 28 children. The relationship between nutritional and health problems was apparent in this study. Possible methods for improving the nutrition and health of the migrant children are discussed.	5	Observational
Dietary Reference Intakes for Vitamin A, Vitamin K, Arsenic, Boron, Chromium, Copper, Iodine, Iron,	Clinical Effects of Inadequate Intake	Because of the role of vitamin A in maintaining the structural integrity of epithelial cells, follicular				

Manganese, Molybdenum, Nickel, Silicon, Vanadium, and Zinc		hyperkeratosis has been observed with inadequate vitamin A intake				
Scientific Opinion on Dietary Reference Values for vitamin A EFSA Journal 2015;13(3):4028 11	Health consequences of deficiency	In low-income countries, vitamin A deficiency in young infants and children has been associated with increased infectious morbidity and mortality, including respiratory infection and diarrhoea	Vitamin A supplements for preventing mortality, illness, and blindness in children aged under 5: systematic review and meta-analysis [8]	Objective To determine if vitamin A supplementation is associated with reductions in mortality and morbidity in children aged 6 months to 5 years. Design Systematic review and meta-analysis. Two reviewers independently assessed studies for inclusion. Data were double extracted; discrepancies were resolved by discussion. Meta-analyses were performed for mortality, illness, vision, and side effects. Data sources Cochrane Central Register of Controlled Trials (CENTRAL) in the Cochrane Library, Medline, Embase, Global Health, Latin American and Caribbean Health Sciences, meta Register of Controlled Trials, and African Index Medicus. Databases were searched to April 2010 without restriction by language or publication status. Eligibility criteria for selecting studies Randomised trials of synthetic oral vitamin A supplements in children aged 6 months to 5 years. Studies of children with current illness (such as diarrhoea, measles, and HIV), studies of children in hospital, and studies of food fortification or β carotene were excluded. Results 43 trials with about 215 633 children were included. Seventeen trials including 194 483 participants reported a 24% reduction in all cause mortality (rate ratio=0.76, 95% confidence interval 0.69 to 0.83). Seven trials reported a 28% reduction in mortality associated with diarrhoea (0.72, 0.57 to 0.91). Vitamin A supplementation was associated with a reduced incidence of diarrhoea (0.85, 0.82 to 0.87) and measles (0.50, 0.37 to 0.67) and a reduced prevalence of vision problems, including night blindness (0.32, 0.21 to 0.50) and xerophthalmia (0.31, 0.22 to 0.45). Three trials reported an increased risk of vomiting within the first 48 hours of supplementation (2.75, 1.81 to 4.19). Conclusions: Vitamin A supplementation is associated with large reductions in mortality, morbidity, and vision problems in a range of settings, and these results cannot be explained by bias. Further placebo controlled trials of vitamin A supplementation in children between 6 and 59 months of age are not required. However, there is a need for further studies comparing different doses and delivery mechanisms (for example, fortification). Until other sources are available, vitamin A supplements should be given to all children at risk of deficiency, particularly in low and middle income countries.	1	Systematic review
Scientific Opinion on Dietary Reference Values for vitamin A EFSA Journal 2015;13(3):4028 11	Health consequences of excess	ULs for children were extrapolated from the UL for adults, based on allometric scaling	Opinion on the Tolerable Upper Intake Level of preformed vitamin A (retinol	This opinion is one in the series of opinions of the Scientific Committee on Food (SCF) on the upper levels of vitamins and minerals. The terms of reference given by the European Commission for this task, the related background and the guidelines used by the Committee to develop tolerable upper intake levels for vitamins and minerals used in this opinion, which were expressed by the SCF on	6	Non-systematic review

		(body weight to the power of 0.75)	and retinyl esters) [9]	19 October 2000, are available on the Internet at the pages of the SCF, at the address: http://www.europa.eu.int/comm/food/fs/sc/scf/index_en.html.		
Scientific Opinion on Dietary Reference Values for vitamin A EFSA Journal 2015;13(3):4028 11	Health consequences of excess	The Panel is aware of other studies which looked at possible associations between preformed vitamin A intake or blood retinol concentration and adverse health outcomes	Vitamin A supplementation and childhood morbidity from diarrhea and respiratory infections: a meta-analysis [10]	Objectives: To perform an updated meta-analysis of the effect of vitamin A supplementation on childhood morbidity from respiratory tract infections and diarrhea. Study design A comprehensive search of the 1966 to 2000 MEDLINE database and review of the reference lists of relevant articles identified 9 randomized controlled trials dealing with morbidity from respiratory infections and diarrhea in children 6 months to 7 years of age and provided "intention-to-treat" data. Results: The combined results indicated that vitamin A supplementation has no consistent overall protective effect on the incidence of diarrhea (relative risk, 1.00; 95% CI, 0.94-1.07) and that it slightly increases the incidence of respiratory tract infections (relative risk, 1.08; 95% CI, 1.05-1.11). Conclusions: High-dose vitamin A supplements are not recommended on a routine basis for all preschool children and should be offered only to individuals or populations with vitamin A deficiency.	1	System atic review
Scientific Opinion on Dietary Reference Values for vitamin A EFSA Journal 2015;13(3):4028 11	Health consequences of excess	The Panel is aware of other studies which looked at possible associations between preformed vitamin A intake or blood retinol concentration and adverse health outcomes	Vitamin A for preventing acute lower respiratory tract infections in children up to seven years of age [11]	Background: Vitamin A supplements are effective for preventing diarrhoea. There are theoretical reasons it might also be effective for acute lower respiratory tract infections (LRTIs), also very common in children, especially in low income countries. Objectives: To assess the effectiveness and safety of vitamin A for preventing acute LRTIs in children up to seven years of age. Search strategy: We searched the Cochrane Central Register of Controlled Trials (CENTRAL) (The Cochrane Library 2007, Issue 2); MEDLINE (1966 to July 2007); EMBASE (1974 to July 2007); and the Chinese Biomedicine Database (CBM) (1976 to July 2007). Selection criteria: Randomised controlled trials (RCTs) that assessed the effectiveness of vitamin A in the prevention of acute LRTI in children up to seven years of age. Data collection and analysis: The review authors independently extracted data and assessed trial quality. Study authors were contacted for additional information. Main results: Most studies found no significant effect of vitamin A on the incidence of acute LRTI, or prevalence of symptoms of acute LRTI. Vitamin A caused an increased incidence of acute LRTI in one study; an increase in cough and fever; and increased symptoms of cough and rapid breathing in two others. Three reported no differences and no protective effect of vitamin A. Two studies reported that vitamin A significantly reduced the incidence of acute LRTI with children with poor nutritional status or weight, but increased it in normal children. Authors' conclusions: This unexpected result is outside our current understanding of the use of vitamin A for preventing acute LRTIs. Accordingly, vitamin A should not be given to all children to prevent acute LRTIs. There is evidence for vitamin A supplements to prevent acute LRTIs in children with low serum retinol or those with a poor nutritional status.	1	System atic review

Scientific Opinion on Dietary Reference Values for vitamin A EFSA Journal 2015;13(3):4028 11	Efficiency of storage	Mean retention was 82.2 ± 2.0 % in healthy children (n = 5) and 57.6 ± 6.0 % in a group of children (n = 8) with infection	Studies on vitamin A absorption in children [12]	No abstract available	3a	Experimental - absorption
Scientific Opinion on Dietary Reference Values for vitamin A EFSA Journal 2015;13(3):4028 11	Efficiency of storage	Mean retention was 82.2 ± 2.0 % in healthy children (n = 5) and 57.6 ± 6.0 % in a group of children (n = 8) with infection	Absorption of labelled vitamin A in children during infection [13]	1. The intestinal absorption of [11,12 3H2] retinyl acetate was studied in five apparently normal children, eight children with respiratory infection and three with gastroenteritis. 2. The absorption of vitamin A was significantly lower in children with respiratory infection or gastroenteritis than in normal children. 3. In the light of these observations, it is suggested that repeated attacks of infections may significantly contribute to the prevalence of vitamin A deficiency in children of poor communities.	3a	Experimental - absorption
Dietary Reference Intakes for Vitamin A, Vitamin K, Arsenic, Boron, Chromium, Copper, Iodine, Iron, Manganese, Molybdenum, Nickel, Silicon, Vanadium, and Zinc	Factors affecting the Vitamin A requirement	Sivakumar and Reddy (1972) demonstrated depressed absorption of labelled vitamin A in children with gastroenteritis and respiratory infections.				
Scientific Opinion on Dietary Reference Values for vitamin A EFSA Journal 2015;13(3):4028 11	Efficiency of storage	Retention in the range of 48–54 % was estimated in healthy Indian children (n = 17; 3–6 years)	Vitamin E supplements and the absorption of a massive dose of vitamin A [14]	The effect of vitamin E supplements on the absorption of a massive dose of vitamin A was studied in 17 normal children. They were divided into four groups. Each child was given 4 to 5 µCi of (11, 12-3 H2) retinyl acetate along with 200,000 IU of cold vitamin A. Group I received only vitamin A, whereas groups II, III, and IV received in addition to vitamin A, 40, 100, and 500 mg dl-alpha tocopherol acetate, respectively. The addition of 40 and 100 mg of vitamin E to the massive dose of vitamin A had no effect on the absorption or retention of vitamin A. When 500 mg of vitamin E was given, there was a significant increase in the intestinal absorption of vitamin A and also an increase in the urinary excretion. There was no significant difference in the retention of vitamin A in these children as compared with those who received vitamin A without vitamin E.	3a	Experimental - absorption

Scientific Opinion on Dietary Reference Values for vitamin A EFSA Journal 2015;13(3):4028 11	Absorption of preformed vitamin A	Absorption remains high even if the amount of ingested preformed vitamin A increases	The prevention of childhood blindness by the administration of massive doses of vitamin A [15]	No abstract available	3a	Experimental - absorption
Scientific Opinion on Dietary Reference Values for vitamin A EFSA Journal 2015;13(3):4028 11	Catabolic losses	estimated a fractional catabolic rate of 2.2 % per day (95 % CI = 1.4–3.0 % per day) in 107 Peruvian children (12–24 months of age)	Population-based plasma kinetics of an oral dose of [2H4]retinyl acetate among preschool-aged, Peruvian children [16]	Background: The deuterated-retinol-dilution technique provides a quantitative estimate of total-body vitamin A (TBVA) stores in adults. To apply the technique to children, information on plasma retinol kinetics in this age group is needed. Objectives: We described the plasma retinol kinetics of an oral dose of [(2)H(4)]retinyl acetate in a population of Peruvian children (12-24 mo of age) in order to examine the relation between TBVA stores and individual plasma isotopic ratios 3 d after the dose and to estimate 1) the time required for the isotope dose to mix with endogenous vitamin A, 2) the fractional catabolic rate for retinol, and 3) TBVA stores. Design: An oral dose of [(2)H(4)]retinyl acetate (14 micromol retinol equivalents) was administered to children (n = 107) to construct a population-level kinetic curve of the plasma ratio of [(2)H(4)]retinol to retinol to estimate equilibration time and the fractional catabolic rate. TBVA stores were estimated by using a modification of the isotope dilution equation for adults. Results: The dose of [(2)H(4)]retinyl acetate fully mixed with endogenous vitamin A 8 d after the dose. The fractional catabolic rate was 0.022/d (95% CI: 0.014, 0.030/d). Mean (+/- SD) TBVA stores were estimated as 0.097 +/- 0.081 mmol (range: 0.016-0.392 mmol). Plasma ratios of [(2)H(4)]retinol to retinol 3 d after the dose were correlated with the inverse of estimated TBVA stores (r = -0.74, P < 0.0001). Conclusions: Compared with previous results in adults, the equilibration time occurred earlier and the estimated system fractional catabolic rate was higher in this population of children. The modified isotope dilution equation provided estimates of hepatic vitamin A concentration that are similar to values reported in US children at autopsy.	3e	Experimental - metabolism
Scientific Opinion on Dietary Reference Values for vitamin A EFSA Journal 2015;13(3):4028 11	Elimination: Breast milk	Preformed vitamin A concentration is higher in colostrum and decreases as lactation progresses	Breast-milk vitamin A as an indicator of the vitamin A status of women and infants [17]	This article reviews the evidence for using breast-milk vitamin A as an indicator of vitamin A status and provides technical information for researchers who want to use this indicator to assess the vitamin A status of women and breast-fed children. Breast-milk vitamin A is a unique indicator for assessing the vitamin A status of lactating women and their breast-fed infants, and has recently been recommended by WHO for use in monitoring global elimination of vitamin A deficiency. Assessing breast-milk vitamin A is less invasive than alternative approaches for assessing a mother's vitamin A status and not at all invasive for	6	Non-systematic review

				her infant. Collection of milk samples in the field is generally feasible and acceptable. Breast-milk vitamin A appears to be an especially good indicator for measuring the impact of vitamin A interventions on women and infants, and for this purpose, it is more responsive than other indicators.		
Scientific Opinion on Dietary Reference Values for vitamin A EFSA Journal 2015;13(3):4028 11	Elimination: Breast milk	Preformed vitamin A concentration is not related to breast milk fat concentration during the first weeks of lactation	Changes in the concentration of carotenoids, vitamin A, alpha-tocopherol and total lipids in human milk throughout early lactation [18]	Background: In mammals the composition of milk changes during early lactation showing a rapid decline in fat-soluble vitamins and a continuous increase in total lipids. Changes in the concentrations of carotenoids, vitamin A, alpha-tocopherol and total lipids in human milk (colostrum, transitory and mature milk) were studied to understand this not well characterised phenomenon. Methods: Colostrum, transitory and mature milk was collected from 21 women and analysed for carotenoids, vitamin A and alpha-tocopherol by HPLC. Results: Total lipids increased from the lowest levels in colostrum (1.5 +/- 1.6 mg/ml) to the highest in transitory milk (3.6 +/- 2.5 mg/ml, p < 0.01). Contrary to this, levels of total carotenoids (236.7 +/- 121.9 ng/ml), vitamin A (1.02 +/- 0.56 microg/ml) and alpha-tocopherol (11.8 +/- 6.3 microg/ml) were highest in colostrum and declined significantly during the first weeks of lactation (63.2 +/- 23.3 ng/ml, 0.33 +/- 0.14 microg/ml, 2.7 +/- 1.1 microg/ml, respectively; p < 0.001). Conclusions: The magnitude of decrease was not the same for all carotenoids and was dependent on the polarity of the carotenoid with the smallest decrease in the polar carotenoids. This might be due to differences in the distribution of carotenoids among plasma lipoproteins and might point to possible selective mechanisms being involved in the transfer of these components in early human milk.	5	Observ ational
Scientific Opinion on Dietary Reference Values for vitamin A EFSA Journal 2015;13(3):4028 11	Elimination: Breast milk	Preformed vitamin A concentration is influenced by maternal vitamin A status	Maternal vitamin A status and its importance in infancy and early childhood [19]	Early fetal vitamin A supplies must be regulated to avoid teratogenic consequences from too little or too much. Late in gestation, adequate maternal vitamin A status is important for newborn reserves and for sustaining adequate breast-milk concentrations. Vitamin A supplements are not needed for most pregnant women in Western countries who consume the recommended dietary allowance during their reproductive years. Increased consumption of vitamin A-rich foods can meet increased needs during lactation. Women in developing countries whose habitual intakes are near basal needs should receive an additional 100 µg retinol equivalents (RE) during pregnancy and 300 µg RE during lactation. Supplements not above 3000 µg RE (10 000 IU) daily are safe for fertile women where circumstances preclude obtaining the needed increment through diet. The first postpartum month is the only safe period during which to provide deficient lactating women with a single high-dose supplement to benefit the mother and breast-feeding infant for several months.	6	Non-system atic review
Scientific Opinion on Dietary Reference Values	Elimination: Breast milk	Mean total retinol concentrations in mature breast milk	Multinational study of major breast milk	Background: Carotenoids in serum vary between countries and within populations with evidence suggesting a qualitative relationship to diet. Breast milk carotenoids furnish a source of vitamin A and potentially provide	5	Observ ational

for vitamin A EFSA Journal 2015;13(3):4028 11		of populations from Western countries range between 229 and 831 µg/L.	carotenoids of healthy mothers [20]	immunoprotection and other health benefits for infants. There have been numerous studies of milk carotenoid concentrations in undernourished populations; however, carotenoid concentrations have not previously been compared in populations of well-nourished mothers. Aim of study: To compare concentrations of five major carotenoid groups: alpha-carotene, beta-carotene, beta-cryptoxanthin, lutein/zeaxanthin, and lycopene in breast milk of healthy women from Australia, Canada, Chile, China, Japan, Mexico, the Philippines, the United Kingdom, and the United States, and to qualitatively compare patterns of dietary intake with milk carotenoid concentrations. Methods: Breast milk collected from healthy lactating women was analyzed for concentrations of five carotenoids and retinol and quantitated relative to total milk lipid. All determinations were performed in a single research laboratory using standardized methodology. Mothers consumed their usual diets and provided a single 24-h dietary recall. Results: Breast milk carotenoid concentrations varied greatly among countries, with the greatest differences in beta-cryptoxanthin (approximately 9-fold) and the least in alpha-carotene and lycopene (approximately 3-fold). Breast milk retinol concentrations varied approximately 2-fold across countries. The provitamin A carotenoids alpha-carotene, beta-carotene, and beta-cryptoxanthin as a group accounted for > 50 % of the carotenoids measured. Total breast milk carotenoids were highest in Japanese and lowest in Philippine mothers. Breast milk beta-carotene concentrations were highest in Chile and lowest in the Philippines. Conclusions: Patterns of breast milk carotenoids were unique to each country and qualitative patterns reflected the dietary carotenoid supply.		
Scientific Opinion on Dietary Reference Values for vitamin A EFSA Journal 2015;13(3):4028 11	Elimination: Breast milk	Mean total retinol concentrations in mature breast milk of populations from Western countries range between 229 and 831 µg/L.	Effect of the stage of lactation in humans on carotenoid levels in milk, blood plasma and plasma lipoprotein fractions [21]	In mammals the composition of milk changes during early lactation, with a rapid decline of fat-soluble vitamins and a continuous increase in total lipids. The mechanisms underlying this phenomenon are not well understood, but might involve selective mechanisms related to mammary uptake or secretion into the milk. Since carotenoids are specifically distributed among the lipoprotein fractions in plasma, the simultaneous determination of carotenoids in plasma, lipoprotein fractions and milk might offer an opportunity to gain insight into this phenomenon. In 21 healthy mothers carotenoids in plasma and lipoprotein fractions were investigated at day 2 and 19 and milk on day 4 and 19 after delivery. Plasma levels of α-tocopherol and cholesterol as well as lutein, zeaxanthin and cryptoxanthin were significantly lower later in lactation (day 19) than shortly after birth ($P < 0.01$). The stage of lactation had no effect on the distribution of carotenoids and α-tocopherol among the plasma lipoprotein fractions. In milk, triacylglycerol increased ($P < 0.01$). In contrast, levels of carotenoids, αtocopherol and vitamin A were highest in colostrum and declined ($P < 0.01$). Because the magnitude of decrease was not the same in all	5	Observ ational

				carotenoids, the carotenoid pattern changed substantially. In colostrum the carotenoid pattern resembled those of plasma and the low-density lipoprotein fraction. In mature milk it was similar to the pattern found in the high density lipoprotein fraction. Based on these observations a selective mechanism might be responsible for the transfer of these components in milk involving different lipoprotein fractions at specific times of lactation.		
Scientific Opinion on Dietary Reference Values for vitamin A EFSA Journal 2015;13(3):4028 11	Elimination: Breast milk	Mean total retinol concentrations in mature breast milk of populations from Western countries range between 229 and 831 µg/L.	Retinol and (alpha)-tocopherol concentrations in breast milk of Turkish lactating mothers under different socio-economic status [22]	Retinol and alpha-tocopherol levels in breast milk of Turkish mothers under different socioeconomic status were investigated. Mature milk samples were collected from 92 lactating mothers living in Izmir and in Manisa, cities of Turkey, who were at 60-90 days of the lactating period. Socio-economic, anthropometric, and dietary data were collected by means of a questionnaire. The body mass index was used to determine the nutritional status. The retinol and alpha-tocopherol contents of breast milk were determined by high-performance liquid chromatography; the concentration of alpha-tocopherol was 9.84 microg/ml whereas retinol levels were 81.5 microg/100 ml. The questionnaire survey was used to determine the level of these vitamins in the daily ration of the women. No significant differences were found in terms of milk retinol and alpha-tocopherol levels for the variables income, educational level and mothers' body mass index.	5	Observ ational
Scientific Opinion on Dietary Reference Values for vitamin A EFSA Journal 2015;13(3):4028 11	Elimination: Breast milk	Mean total retinol concentrations in mature breast milk of populations from Western countries range between 229 and 831 µg/L.	Influence of the lactating women diet on the concentration of the lipophilic vitamins in human milk [23]	The aim of the study was to assess the intake of vitamins A and E by Polish breast feeding mothers and the correlation between the intake of these vitamins and their concentration in the maternal milk. Dietary intake was assessed by triple 24 h diet recall questionnaire. Milk samples were collected and the content of vitamin A and E was determined. The mean intake of vitamin E (7.7±3.4 mg/day) covered 54.7% of the recommended value. The mean vitamin A content in daily food rations (DFR) -1012±735 µg- allowed to cover 63.2% of the requirement. The mean concentration of vitamin A in milk was 57.07±29.3 µg/100 mL. There was a correlation of the content of vitamin A in milk and the intake of this vitamin (r = 0.371) and the intake of essential fatty acids (r = 0.455) also. The mean concentration of the vitamin E in the milk (413.1±194.4 mg/100 mL), statistically significant correlated with its intake in DFR (r = 0.483). DFR of breast- feeding mothers, were characterized by insufficient supply of vitamins A and E. The dietary intake of these vitamins correlated with their concentration in maternal milk.	5	Observ ational
Scientific Opinion on Dietary Reference Values for vitamin A EFSA Journal 2015;13(3):4028 11	Elimination: Breast milk	Mean total retinol concentrations in mature breast milk of populations from Western countries	Rapid sample preparation procedure for determination of retinol and alpha-tocopherol in	The liposoluble vitamins (retinol and α-tocopherol) concentration in human breast milk is of a cardinal knowledge especially for nutrition of prematurely born. It enables the feeding optimization of these important micronutrients for preterm infants. The novel rapid liquid-liquid extraction procedure for human breast milk investigation was developed and validated according to FDA guidelines. The recovery of retinol was 82-90% measured at three concentration	5	Observ ational

		range between 229 and 831 µg/L.	human breast milk [24]	levels 1.0, 2.5 and 5.0 µmol/L, for α-tocopherol 92-109% at concentration levels 2.5, 5.0 and 10.0 µmol/L. The repeatability of extraction procedure expressed as relative standard deviation was 3.26% for retinol and 4.79% for α-tocopherol. Developed extraction procedure was applied on 120 human breast milk samples. The separation of vitamins was completed using advantages of a monolithic column which accomplished demands of acceleration made by modern bio-analytical HPLC methodology. The analytes of interest were detected by diode-array detector at wavelengths 325 nm for retinol and 290 nm for α-tocopherol.		
Scientific Opinion on Dietary Reference Values for vitamin A EFSA Journal 2015;13(3):4028 11	Elimination: Breast milk	Based on a mean milk transfer of 0.8 L/day and a concentration of retinol in breast milk of 530 µg/L taken as the midpoint of the range of means (229–831 µg/L), a secretion of 424 µg/day of retinol in breast milk is estimated during the first six months of lactation	Nutrient adequacy of exclusive breastfeeding for the term infant during the first six months of life [25]	This review, which was prepared as part of the background documentation for a WHO expert consultation, evaluates the nutrient adequacy of exclusive breastfeeding for term infants during the first 6 months of life. Nutrient intakes provided by human milk are compared with infant nutrient requirements. To avoid circular arguments, biochemical and physiological methods, independent of human milk, are used to define these requirements. The review focuses on human-milk nutrients, which may become growth limiting, and on nutrients for which there is a high prevalence of maternal dietary deficiency in some parts of the world; it assesses the adequacy of energy, protein, calcium, iron, zinc, and vitamins A, B6, and D. This task is confounded by the fact that the physiological needs for vitamins A and D, iron, zinc – and possibly other nutrients – are met by the combined availability of nutrients in human milk and endogenous nutrient stores. In evaluating the nutrient adequacy of exclusive breastfeeding, infant nutrient requirements are assessed in terms of relevant functional outcomes. Nutrient adequacy is most commonly evaluated in terms of growth, but other functional outcomes, e.g. immune response and neurodevelopment, are also considered to the extent that available data permit. This review is limited to the nutrient needs of infants. It does not evaluate functional outcomes that depend on other bioactive factors in human milk, or behaviours and practices that are inseparable from breastfeeding, nor does it consider consequences for mothers. In determining the optimal duration of exclusive breastfeeding in specific contexts, it is important that functional outcomes, e.g. infant morbidity and mortality, also are taken into consideration.	6	Non-systematic review
Scientific Opinion on Dietary Reference Values for vitamin A EFSA Journal 2015;13(3):4028 11	Interaction with other nutrients	Serum retinol concentration was positively associated with serum iron and ferritin concentrations in children	Iron metabolism and vitamin A deficiency in children in northeast Thailand [26]	To investigate the association between vitamin A and iron metabolism, two studies were carried out: a cross-sectional study and an intervention trial. The cross-sectional analysis was carried out in 1060 children aged 1-8 y. Multiple-regression analysis was used to adjust for effects of age, gender, indices of the protein nutritional status, and infections. Retinol was significantly associated with hematocrit, serum Fe, transferrin, ferritin, and saturation of transferrin (%ST). To obtain further evidence as to whether this observed association is a causal one, an intervention trial was carried out. After collection of the baseline data of 300 children, 166 children with a hemoglobin concentration less than 7.5	2	RCT

				mmol/L were selected. A random sub-sample of 78 children received vitamin A capsules; the other children served as control subjects. Two months after supplementation significant differences, adjusted for age, were found for retinol, retinol-binding protein, serum Fe, and %ST between the supplemented and the control group. After 4 mo none of the indices were found to be significantly different between the supplemented and the control group. Periodic massive doses of vitamin A may play a role in improving the Fe status as well.		
Scientific Opinion on Dietary Reference Values for vitamin A EFSA Journal 2015;13(3):4028 11	Interaction with other nutrients	Iron supplementation combined with vitamin A was more effective than iron alone in improving haemoglobin concentrations in anaemic children	Supplemental vitamin A improves anemia and growth in anemic school children in Tanzania [27]	We conducted a randomized controlled trial of the effects of dietary supplements on anemia, weight and height in 136 anemic school children from a low socioeconomic background in Bagamoyo District schools in Tanzania. The aim of the current study was to investigate the impact of dietary supplements on anemia and anthropometric indices of anemic school children. The supplements were vitamin A alone, iron and vitamin A, iron alone or placebo, administered in a double-blinded design for 3 mo. All supplements were provided with local corn meals. Hemoglobin concentration, body weight and height were measured at baseline and at follow-up after supplementation. Vitamin A supplementation increased the mean hemoglobin concentration by 13.5 g/L compared with 3.5 g/L for placebo [P < 0.0001, 95% confidence interval (CI) 6.19-13.57), the mean body weight by 0.6 kg compared with 0.2 kg for placebo (P < 0.0001, 95% CI 0.19-0.65) and the mean height by 0.4 cm compared with 0.1 cm for placebo (P = 0.0009, 95% CI 0.08-0.42). However, the group of children who received combined vitamin A and iron supplementation had the greatest improvements in all indicators compared with placebo (18.5 g/L, P < 0.0001, 95% CI 14.81-22.23; 0.7 kg, P < 0. 0001, 95% CI 0.43-0.88 and 0.4 cm, P < 0.0001, 95% CI 0.22-0.56 for hemoglobin, weight and height, respectively). It is likely that vitamin A supplementation may have a useful role in combating the problems of vitamin A deficiency and anemia, as well as in improving children's growth, in developing countries.	2	RCT
Scientific Opinion on Dietary Reference Values for vitamin A EFSA Journal 2015;13(3):4028 11	Interaction with other nutrients	correlations between anaemia and vitamin A deficiency and the amelioration of anaemia by improving vitamin A status in deficient populations	Effects of vitamin A supplementation on iron status indices and iron deficiency anaemia: a randomized controlled trial [28]	Iron deficiency anaemia (IDA) is the most common nutritional deficiency in the world including developed and developing countries. Despite intensive efforts to improve the quality of life of rural and aboriginal communities in Malaysia, anaemia and IDA are still major public health problems in these communities particularly among children. A randomized, double-blind, placebo-controlled trial was conducted on 250 Orang Asli (aboriginal) schoolchildren in Malaysia to investigate the effects of a single high-dose of vitamin A supplementation (200,000 IU) on iron status indices, anaemia and IDA status. The effect of the supplement was assessed after 3 months of receiving the supplements; after a complete 3-day deworming course of 400 mg/day of albendazole tablets. The prevalence of anaemia was found to be high: 48.5% (95% CI=42.3, 54.8). Moreover, 34% (95% CI=28.3, 40.2) of the children had IDA, which accounted for	2	RCT

				70.1% of the anaemic cases. The findings showed that the reduction in serum ferritin level and the increments in haemoglobin, serum iron and transferrin saturation were found to be significant among children allocated to the vitamin A group compared to those allocated to the placebo group ($p<0.01$). Moreover, a significant reduction in the prevalence of IDA by almost 22% than prevalence at baseline was reported among children in the vitamin A group compared with only 2.3% reduction among children in the placebo group. In conclusion, vitamin A supplementation showed a significant impact on iron status indices and IDA among Orang Asli children. Hence, providing vitamin A supplementation and imparting the knowledge related to nutritious food should be considered in the efforts to improve the nutritional and health status of these children as a part of efforts to improve the quality of life in rural and aboriginal communities.		
Scientific Opinion on Dietary Reference Values for vitamin A EFSA Journal 2015;13(3):4028 11	Retinol equivalents – conversion factors	The amount of β-carotene in oil required to form 1 µg retinol was 2.4 µg (95 % CI = 2.1–2.7 µg)	Bioefficacy of beta-carotene dissolved in oil studied in children in Indonesia [29]	Background: More information on the bioefficacy of carotenoids in foods ingested by humans is needed. Objective: We aimed to measure the time required for isotopic enrichment of beta-carotene and retinol in serum to reach a plateau, the extent of conversion of beta-carotene dissolved in oil with use of beta-carotene and retinol specifically labeled with 10 (13)C atoms, and the intraindividual variation in response. Design: Indonesian children aged 8--11 y (n = 35) consumed 2 capsules/d, 7 d/wk, for < or =10 wk. Each capsule contained 80 microg [12,13,14,15,20,12',13',14',15',20'-(13)C(10)]beta-carotene and 80 microg [8,9,10,11,12,13,14,15,19,20-(13)C(10)]retinyl palmitate. Three blood samples were drawn per child over a period of < or =10 wk. HPLC coupled with atmospheric pressure chemical ionization liquid chromatography-mass spectrometry was used to measure the isotopic enrichment in serum of retinol with [(13)C(5)]retinol and [(13)C(10)]retinol and of beta-carotene with [(13)C(10)]beta-carotene. The beta-carotene in the capsules used had a cis-trans ratio of 3:1. Results: Plateau isotopic enrichment was reached by day 21. The amount of beta-carotene in oil required to form 1 microg retinol was 2.4 microg (95% CI: 2.1, 2.7). The amount of all-trans-beta-carotene required to form 1 microg retinol may be lower. Conclusions: The efficiency of conversion of this beta-carotene in oil was 27% better than that estimated previously (1.0 microg retinol from 3.3 microg beta-carotene with an unknown cis-trans ratio). The method described can be extended to measure the bioefficacy of carotenoids in foods with high precision, requiring fewer subjects than other methods.	3a	Experimental - absorption
Scientific Opinion on Dietary Reference Values for vitamin A EFSA Journal 2015;13(3):4028 11	Retinol equivalents – conversion factors	conversion factors of pure β-carotene, β-carotene from Golden Rice and β-carotene from spinach to retinol	β-carotene in Golden Rice is as good as β-carotene in oil at providing vitamin A to children [30]	Background: Golden Rice (GR) has been genetically engineered to be rich in β-carotene for use as a source of vitamin A.<br>Objective: The objective was to compare the vitamin A value of β-carotene in GR and in spinach with that of pure β-carotene in oil when consumed by children.<br>Design: Children (n = 68; age 6-8 y) were randomly assigned to consume GR or spinach (both grown in a nutrient solution containing 23 atom% ^2H_2O) or $[^2H_8]$β-	3a	Experimental - absorption

		were 2.0 ± 0.9, 2.3 ± 0.8 and 7.5 ± 0.8 to 1, respectively		carotene in an oil capsule. The GR and spinach β-carotene were enriched with deuterium (^2H) with the highest abundance molecular mass (M) at M(β-C)+$^2H_{10}$. [$^{13}C_{10}$]Retinyl acetate in an oil capsule was administered as a reference dose. Serum samples collected from subjects were analyzed by using gas chromatography electron-capture negative chemical ionization mass spectrometry for the enrichments of labeled retinol: M(retinol)+4 (from [$^2H_8$]β-carotene in oil), M(retinol)+5 (from GR or spinach [$^2H_{10}$]β-carotene), and M(retinol)+10 (from [$^{13}C_{10}$]retinyl acetate). Results: Using the response to the dose of [$^{13}C_{10}$]retinyl acetate (0.5 mg) as a reference, our results (with the use of AUC of molar enrichment at days 1, 3, 7, 14, and 21 after the labeled doses) showed that the conversions of pure β-carotene (0.5 mg), GR β-carotene (0.6 mg), and spinach β-carotene (1.4 mg) to retinol were 2.0, 2.3, and 7.5 to 1 by weight, respectively. Conclusions: The β-carotene in GR is as effective as pure β-carotene in oil and better than that in spinach at providing vitamin A to children. A bowl of ~100 to 150 g cooked GR (50 g dry weight) can provide ~60% of the Chinese Recommended Nutrient Intake of vitamin A for 6-8-y-old children.		
Scientific Opinion on Dietary Reference Values for vitamin A EFSA Journal 2015;13(3):4028 11	Liver retinol concentration	Post-mortem liver analysis indicated concentrations of retinol ranging from 10 to 1 807 µg/g liver (0.03 to 6.3 µmol/g) in Western countries	Vitamin A reserves of Canadians [31]	A survey of vitamin A and carotene stores of Canadians at five major centres across Canada was completed. Vitamin A and carotene analyses were performed on approximately 100 human liver specimens obtained at necropsy from each location. Age influenced liver vitamin A stores. Children between 1 and 10 years of age had the highest vitamin A stores while a trend toward lower liver stores occurred between 20 and 40 years of age. Females had higher liver carotene stores than males but there was no such difference in vitamin A stores. SUBJECTS FROM ALL LOCATIONS WERE CLASSIFIED ACCORDING TO CAUSE OF DEATH: accidental, heart and coronary artery diseases, cancer, respiratory diseases and a miscellaneous disease group. The mean liver vitamin A and carotene stores of the accidental death group differed only from the cancer group. In contrast to the disease groups, no case with undetectable vitamin A was found in the accidental death group. The mean vitamin A and carotene stores of Vancouver subjects were generally higher than those for the other locations. Montreal showed more values in the 0-40 mug. per g. range than the other locations. Vancouver had the least number (15%), with Halifax, Ottawa and Winnipeg being intermediate (32%). These data suggest the need for improved nutrition, prophylactic treatment in disease states and the need for further research on the utilization and metabolism of vitamin A.	3d	Experi mental - stores
Scientific Opinion on Dietary Reference Values for vitamin A EFSA	Liver retinol concentration	Post-mortem liver analysis indicated concentrations of retinol ranging from 10 to 1 807	Liver Stores of Vitamin A in a Normal Population Dying Suddenly or	Data are presented on liver concentration of vitamin A in a normal population dying suddenly or rapidly from unnatural causes in New York City. Data were obtained from 101 specimens. A mean value of 126 µg/g wet tissue and a median of 66 µg/g was found. The range in values was from 7 to 668 µg/g. Thirty-five percent of the specimens contained 40 µg/g or less of vitamin A.	3d	Experi mental - stores

Journal 2015;13(3):4028 11		µg/g liver (0.03 to 6.3 µmol/g) in Western countries	Rapidly from Unnatural Causes in New York City [32]	Children under 10 years of age had the highest stores of vitamin A. The black population had significantly lower reserves than the white population. Reduced liver stores and an increased percentage of specimens with vitamin A levels under 40 µg/g were found in victims who died from an overdose of heroin or from bullet or stab wounds. Possible reasons for this relationship are discussed. Serum levels of vitamin A were of no assistance in predicting liver reserves. No serum level under 20 µg/100 ml was found even when the liver concentration was reduced to 11 µg/g wet tissue.		
Scientific Opinion on Dietary Reference Values for vitamin A EFSA Journal 2015;13(3):4028 11	Liver retinol concentration	Post-mortem liver analysis indicated concentrations of retinol ranging from 10 to 1 807 µg/g liver (0.03 to 6.3 µmol/g) in Western countries	Vitamin A and carotene levels of a selected population in metropolitan Washington, D. C [33]	Liver samples obtained from subjects who died acute traumatic deaths or who died from various diseases in metropolitan Washington, D.C. were analyzed for vitamin A and carotene. Children under 2 months old had the lowest mean liver concentrations of vitamin A and carotene; children from 2 months to 10 years old and adults over 70 years old had the highest mean concentrations. Of the samples analyzed, 24% had less than 50 µg vitamin A/g liver and 3.3% of the samples had over 1,000 µg/g. Mean values of 211 µg of vitamin A/g and 5.6 µg of carotene/g were found in the livers of accident victims. The black male had a considerably lower concentration of vitamin A and carotene than did other groups within certain age ranges. Diseases, especially hepatic disease, appear to present an additional burden on vitamin A reserves. The large percentage of low levels of vitamin A found can probably be ascribed to some nutritional inadequacies, whereas the high levels may be due to the wide use of vitamin supplements by infants, children, and adults over 70 years old.	3d	Experi mental - stores
Scientific Opinion on Dietary Reference Values for vitamin A EFSA Journal 2015;13(3):4028 11	Liver retinol concentration	Post-mortem liver analysis indicated concentrations of retinol ranging from 10 to 1 807 µg/g liver (0.03 to 6.3 µmol/g) in Western countries	Vitamin E, selenium, iron, and vitamin A content of livers from Sudden Infant Death Syndrome cases and control children: Interrelationships and possible significance [34]	No abstract available	3d	Experi mental - stores
Scientific Opinion on Dietary Reference Values for vitamin A EFSA Journal 2015;13(3):4028 11	Liver retinol concentration	Post-mortem liver analysis indicated concentrations of retinol ranging from 10 to 1 807 µg/g liver (0.03 to	A survey of human liver reserves of retinol in London [35]	1. The retinol content of human liver tissue, obtained at autopsy from mortuaries in the London area, was determined in a group of 364 subjects. 2. Tissue samples from the central portion of the right lobe of the liver were saponified, extracted with light petroleum (b.p. 40–60°) and retinol assayed colorimetrically using the trifluoroacetic acid procedure. 3. The frequency distribution of retinol reserves was skewed to the right (positive skewness). The	3d	Experi mental - stores

		6.3 µmol/g) in Western countries		mean retinol content for the entire group was 252 mg/kg, with a median of 198 mg/kg and a range of 0–1201 mg/kg. Of the subjects, 49% had reserves in the range 100–300 mg/kg (regarded as the'normal range' for liver retinol) while 5% had reserves below 40 mg/kg (the arbitrary 'cut-off' point below which individuals are considered to be at risk). Only one subject had no detectable retinol reserves. On the other hand, 11 % of the subjects had reserves exceeding 500 mg/kg. 4. No sex-related differences were apparent, the median retinol reserves of male and female subjects being 190 and 202 mg/kg respectively. 5. Median retinol reserves varied markedly with age. They were relatively low (121 mg/kg) in infancy. but were approximately three times greater in childhood, adolescence and young adulthood. Thereafter, reserves declined gradually with increasing age to a low of 113 mg/kg in subjects over the age of 90 years. 6. When subjects were classified according to primary cause of death, the median reserves were as follows (mg/kg): accidental deaths 270, cancer 207, cardiovascular diseases 204, intestinal diseases 178, nervous diseases 164, hepatic diseases 158, respiratory diseases 141, sudden infant death syndrome 127. 7. Retinol reserves in London are substantially higher than those reported from North America and are exceeded only by those reported from New Zealand and Ghana. It is therefore concluded, from the results of this survey and also from official estimates of the dietary intake of retinol and provitamin A carotenoids, that vitamin A status in London, and probably also in the rest of Britain, is quite satisfactory.		
Scientific Opinion on Dietary Reference Values for vitamin A EFSA Journal 2015;13(3):4028 11	Liver retinol concentration	Post-mortem liver analysis indicated concentrations of retinol ranging from 10 to 1 807 µg/g liver (0.03 to 6.3 µmol/g) in Western countries	Size and composition of liver vitamin A reserves of human beings who died of various causes [36]	Postmortem livers from 77 "normal" persons, 37 patients with neoplastic disease, 10 subjects with liver insufficiency, and 7 infants were analysed for free and esterified retinol. The average concentrations of total vitamin A for the members of each group were 597, 551, 289, and 162 micrograms/g wet liver, respectively. Compared with corresponding control values, both cancer victims and patients with liver disease had significantly lower hepatic vitamin A levels. With regard to the composition of the liver vitamin A reserves, our results show that approximately 97% of this vitamin was present as retinyl ester. Additionally, minute amounts of retinol were also found in most of the liver specimens analysed. In "normal" subjects the major ester fraction recovered was palmitate/oleate followed by stearate and myristate/linoleate. By contrast, the second most abundant fatty acid in the retinyl ester fraction of cancer victims was myristic/linoleic acid together with significantly smaller quantities of stearic acid. In tissue samples obtained from patients with liver disorders, however, the myristate/linoleate fraction was increased and therefore nearly equal amounts of both retinyl stearate and myristate/linoleate were present.	3d	Experi mental - stores

Scientific Opinion on Dietary Reference Values for vitamin A EFSA Journal 2015;13(3):4028 11	Liver retinol concentration	Analysis of liver biopsy samples performed in low-income countries showed hepatic concentrations of 17–141 µg/g (0.1–0.5 µmol/g)	Plasma and liver concentration of vitamin A in a normal population of urban Thai [37]	Vitamin A concentrations in the plasma and liver of 84 accident victims in Bangkok were determined. The group, all of whom were in apparent good health prior to the accidents, consisted of 68 males and 16 females, varied in age from 2 to 66 years, and represented a spectrum of occupations. Median and mean plasma values were 36.6 µg/100 ml and 36.2 ± 16.1 µg/100 ml, with a range from 6.8 to 82 µg/100 ml. The median, mean, and range of liver values were 89 µg/g wet tissue, 183 µg/g, and 7.5 to 3,200 µg/g, respectively. Plasma values above 10 µg/100 ml and liver stores were not at all correlated in our sample. Median and mean liver values were lowest in the 11- to 20-year age group, next lowest in the 2- to 10-year age group, and tended generally to increase with age. Median and mean plasma and liver values were also influenced by socioeconomic status, being highest in government officials and merchants and lowest in farmers and unskilled laborers. Women tended to have slightly higher plasma levels and slightly lower liver reserves than men. Median liver reserves in victims of violent confrontations (gunshot or knife wounds) tended to be higher than in persons dying in auto accidents or by electrocution, which, in turn, were higher than in victims of self-inflicted or solitary accidents. Only one of 79 persons older than 10 years of age had liver reserves less than 10 µg/g, and none of the 5 children less than 10 years of age had liver values less than 20 µg/g. We concluded that the vitamin A status of the urban Thai is quite satisfactory, although some attention should probably be given to improving the vitamin A nutriture of young people in the city under 20 years of age. Because vitamin A deficiency is still found in Thailand, we wish to stress that this survey is not representative of the country as a whole.	3d	Experi mental - stores
Scientific Opinion on Dietary Reference Values for vitamin A EFSA Journal 2015;13(3):4028 11	Liver retinol concentration	Analysis of liver biopsy samples performed in low-income countries showed hepatic concentrations of 17–141 µg/g (0.1–0.5 µmol/g)	Liver vitamin A reserves of neonates, preschool children and adults dying of various causes in Salvador, Brazil [38]	No abstract available	3d	Experi mental - stores
Scientific Opinion on Dietary Reference Values for vitamin A EFSA Journal 2015;13(3):4028 11	Liver retinol concentration	Analysis of liver biopsy samples performed in low-income countries showed hepatic concentrations of 17–141 µg/g (0.1–0.5 µmol/g)	Liver levels of retinol in unselected necropsy specimens: a prevalence survey of vitamin A	Total retinol was analyzed in 517 necropsy specimens from children 0 to 14 yr of age, deceased of various causes in Recife, Brazil, and from eight persons, 10 to 59 yr of age, who died violently. Age and nutritional status, in that order, were found to be the major determinants of the prevalence of low reserves, children less than 1 yr of age showing the highest prevalence of deficient values. These data were extrapolated to the live population less than 5 yr of age, categorized by age and nutritional status. By using the criteria that liver reserves of vitamin A less than 5 and 20 micrograms/g denote states of high risk and inadequacy,	3d	Experi mental - stores

			deficiency in Recife, Brazil [39]	respectively, approximately 3 and 17% of children 0 to 4 yr of age in the live population of Recife were estimated to fall in the high risk and inadequate status group, respectively. None of the persons who died violently was at high risk and only one had inadequate reserves. These estimates agree with previous prevalence figures from clinical and biochemical surveys. Thus, the direct assessment of concentrations of vitamin A in the liver at necropsy is a simple, inexpensive, and noninvasive procedure for estimating the vitamin A status of children.		
Scientific Opinion on Dietary Reference Values for vitamin A EFSA Journal 2015;13(3):4028 11	Plasma/serum retinol concentration	The prevalence of values below 200 µg/L (0.7 µmol/L) is a generally accepted population cut-off for preschool-age children to indicate risk of inadequate vitamin A status	Indicators for assessing vitamin A deficiency and their application in monitoring and evaluating intervention programmes [40]	No abstract available		Published guidelines
Vitamin and mineral requirements in human nutrition. 2004: World Health Organization	Indicators of vitamin A deficiency	The prevalence of values below 0.70mmol/l is a generally accepted population cutoff for preschool-age children to indicate risk of inadequate vitamin A status				
Scientific Opinion on Dietary Reference Values for vitamin A EFSA Journal 2015;13(3):4028 11	Plasma/serum retinol concentration	The prevalence of values below 200 µg/L (0.7 µmol/L) is a generally accepted population cut-off for preschool-age children to indicate risk of inadequate vitamin A status	Serum retinol concentrations for determining the prevalence of vitamin A deficiency in populations [41]	No abstract available		Published guidelines

Scientific Opinion on Dietary Reference Values for vitamin A EFSA Journal 2015;13(3):4028 11	Plasma/serum retinol concentration	values above 300 µg/L (1.05 µmol/L) indicate an adequate status related to the absence of clinical signs of deficiency	Analysis of vitamin A data from the health and nutrition examination surveys [42]	An Expert Panel on Vitamin A Nutriture assessed serum vitamin A values and related data collected during the first and second National Health and Nutrition Examination Surveys (NHANES I and NHANES II) and the southwest component of the Hispanic Health and Nutrition Examination Survey (SW HHANES). The appropriateness of the analytical methodologies and adequacy of the quality-control procedures used to obtain the serum vitamin A values were evaluated. Age-specific guidelines for the interpretation of serum vitamin A values in selected low ranges (less than 20, 20-24 and 25-29 micrograms/dl) were developed, and estimates of the prevalence of serum vitamin A values in these ranges were presented for each of the three surveys.	5	Observ ational
Scientific Opinion on Dietary Reference Values for vitamin A EFSA Journal 2015;13(3):4028 11	Plasma/serum retinol concentration	values above 300 µg/L (1.05 µmol/L) indicate an adequate status related to the absence of clinical signs of deficiency	Serum vitamin A distribution curve for children aged 2-6 y known to have adequate vitamin A status: a reference population [43]	Serum vitamin A was determined before and 30-45 d after the administration of 60.6 mg (212 mumol) vitamin A to 544 Brazilian children residing in slum areas of Recife. The frequency-distribution curves were compared in a subgroup of children whose vitamin A status was assessed initially by the relative-dose-response (RDR) test. The curves of children with negative (adequate status) and positive (inadequate status) RDR tests were different. The difference disappeared after supplementation. The shape of the distribution curve after supplementation was close to normal with a mean, median, and 95% confidence interval of 1.78 +/- 0.49, 1.68, and 1.02-2.90 mumol/L, respectively. The postsupplementation curve derived from this underprivileged child population may serve as a reference for diagnostic, surveillance, and program-evaluation purposes.	3d	Experi mental - stores
Vitamin and mineral requirements in human nutrition. 2004: World Health Organization	Indicators of vitamin A deficiency	The prevalence of values… above 1.05mmol/l to indicate an adequate status				
Dietary Reference Intakes for Vitamin A, Vitamin K, Arsenic, Boron, Chromium, Copper, Iodine, Iron, Manganese, Molybdenum, Nickel, Silicon, Vanadium, and Zinc	Selection of indicators for estimating the requirement for Vitamin A	In the United States, serum retinol concentration is rarely low (< 0.7 µmol/ L) in more than 5 percent of preschool children, although 20 to 60 percent may exhibit concentrations between 0.70 and 1.05 µmol/L				
Scientific Opinion on Dietary Reference Values	Plasma/serum retinol concentration	Infection can lower mean plasma/serum	Influence of morbidity on serum retinol of	Serum retinol concentrations decrease during illness and thus may not accurately reflect the vitamin A status of populations with a high prevalence of illness. To quantify the contribution of illness to low serum retinol in a field study	5	Observ ational

for vitamin A EFSA Journal 2015;13(3):4028 11		retinol concentration by as much as 25 %, independently of vitamin A intake	children in a community-based study in northern Ghana [44]	of children aged 6-59 mo in northern Ghana, serum retinol values were compared with two indicators of recent illness; symptoms reported by parents and acute-phase protein concentrations in serum. Serum retinol was not associated with symptoms of illness but showed a significant negative correlation with both alpha 1-acid glycoprotein (AGP) and serum amyloid A (SAA). Elevated AGP was associated with a 24% decrease in mean serum retinol. A large proportion of asymptomatic children had elevated AGP or SAA concentrations, suggesting that subclinical infections may have had important effects on serum retinol. A significant negative correlation between malaria parasite density and serum retinol indicated that malaria may have been one of the subclinical infections responsible. Measurement of AGP may improve interpretation of serum retinol data from populations with a high prevalence of morbidity.		
Vitamin and mineral requirements in human nutrition. 2004: World Health Organization	Indicators of vitamin A deficiency	clinical and subclinical infections can lower serum levels of vitamin A on average by as much as 25%, independently of vitamin A intake				
Scientific Opinion on Dietary Reference Values for vitamin A EFSA Journal 2015;13(3):4028 11	Dietary intake	Nutrient intakes presented.	Finravinto 2012 -tutkimus - The National FINDIET 2012 Survey [45]	The nutrition of the Finnish adult population has been monitored since 1982 as part of the FINRISKI study on chronic disease risk factors and general health. The latest Finravinto data was collected in spring 2012. The Finravinto 2012 study provides a cross-section of Finnish adults' food consumption and food intake. The study examines the meal rhythm of the Finnish adult population, the use of mass eating, food consumption, the intake of nutrients from food and foodstuffs, and the use of food supplements. The report contains baseline results that are related to the nutritional recommendations in force at the time of the study. Information on the diet of Finns is needed to assess and monitor the nutritional situation of the population and to identify risk groups. Thorough knowledge of the current situation is needed as a basis for planning nutrition policy measures. Data on food intake and nutrient intake are also widely used in nutritional and toxicological risk assessment in Finland and Europe.	5	Observational
Scientific Opinion on Dietary Reference Values for vitamin A EFSA Journal 2015;13(3):4028 11	Dietary intake	Nutrient intakes presented.	Ernährungsphysiologische Auswertung einer repräsentativen Verzehrsstudie bei Säuglingen und Kleinkindern VELS mit dem Instrumentarium der DONALD Studie [46]	No abstract available	5	Observational
Scientific Opinion on Dietary	Dietary intake	Nutrient intakes presented.	The third Italian national food	Background and aims Italian National Food Consumption Survey, INRAN-SCAI 2005–06, is the third national food consumption survey performed in Italy. This	5	Observational

Reference Values for vitamin A EFSA Journal 2015;13(3):4028 11			consumption survey, INRAN-SCAI 2005–06–part 1: nutrient intakes in Italy [47]	study describes energy and nutrient intakes in Italy. Methods and results A national cross-sectional food consumption survey was conducted using consecutive 3-day food records between October 2005 and December 2006. A sample of 3323 males and females aged 0.1–97.7 years living in private households was investigated. Individual food records were converted into energy and nutrient intakes with the use of recently updated national food composition databases. For each subject, intakes of energy and of 27 nutrients were calculated, including six minerals (i.e., iron, calcium, phosphorus, magnesium, potassium and zinc) and 10 vitamins (i.e., thiamine, riboflavin, vitamin C, vitamin B6, retinol, β-carotene, vitamin A as retinol equivalents (REs), vitamin E, vitamin D and vitamin B12). On average, 36% of calories appeared to derive from fat (11% from saturated fatty acids) and 45% from available carbohydrates (15% from soluble carbohydrates). Conclusions The results of the INRAN-SCAI 2005–06 survey in terms of nutrient intakes provide an important piece of information for nutrition surveillance of the population and may also be used to identify priorities for further research.		
Scientific Opinion on Dietary Reference Values for vitamin A EFSA Journal 2015;13(3):4028 11	Dietary intake	Nutrient intakes presented.	Headline results from Years 1,2 and 3 (combined) of the Rolling Programme (2008/2009-2010/11) [48]	No abstract available	5	Observ ational
Scientific Opinion on Dietary Reference Values for vitamin A EFSA Journal 2015;13(3):4028 11	Dietary intake	Nutrient intakes presented.	Ernährungsstudie als KiGGS-Modul (EsKiMo) [49]	No abstract available	5	Observ ational
Scientific Opinion on Dietary Reference Values for vitamin A EFSA Journal 2015;13(3):4028 11	Dietary intake	Nutrient intakes presented.	Étude Individuelle Nationale des Consommations Alimentaires 2 (INCA 2)(2006–2007) [50]	No abstract available	5	Observ ational
Scientific Opinion on Dietary Reference Values for vitamin A EFSA	Dietary intake	Nutrient intakes presented.	Dutch National Food Consumption Survey 2007-2010: Diet of	Dutch people still consume insufficient fruit, vegetables, fish and fibre: Improvement of the type of fat in the diet by the reduction of trans fatty acids in foods is a favourable development. However, the proportion of saturated fatty acids in the diet is still high and overweight is common. These are conclusions of a recent food consumption survey by the National Institute for Public Health and	5	Observ ational

Journal 2015;13(3):4028 11			children and adults aged 7 to 69 years [51]	the Environment (RIVM), carried out between 2007 and 2010 among children and adults in the Netherlands. The importance of a healthy dietary pattern: a healthy dietary pattern is important to prevent obesity and chronic diseases. The survey provides insight into food consumption which can be used to stimulate healthier dietary patterns. This can be achieved by changes in both the food supply and consumer behaviour. Intake of vitamins and minerals: the survey also shows that some people have intakes below the recommended amounts for some vitamins and minerals, such as vitamins A, B1, C, E, magnesium, potassium and zinc. More information on the possible health effects of these low intakes is desirable. Furthermore, age groups with specific higher intake requirements often do not meet these, i.e. concerning folate (for women with a pregnancy wish), vitamin D (people above 50), iron (women of childbearing age) and calcium (adolescents). This observation underlines the advice of the Health Council of the Netherlands to these specific groups with regard to the use of folate and vitamin D supplements. The effects on health of low iron and calcium intakes are unclear; more research is needed in this area. Use of food consumption data: The last survey that included information on food consumption in the Dutch population was conducted in 1997/1998. The recent survey (2007-2010) provides more detailed information. The data from this survey can be used to support policy regarding healthy and safe foods, for improving food supply and in food education and research.		
Scientific Opinion on Dietary Reference Values for vitamin A EFSA Journal 2015;13(3):4028 11	Vitamin A intake and health consequences	A number of intervention studies in children have assessed the effect of vitamin A supplementation on the risk of (premature) death, and the incidence and severity of diarrhoea, measles and lower respiratory tract infections	Vitamin A supplementation in malnourished Sudanese children [52]	No abstract available	2	RCT
Dietary Reference Intakes for Vitamin A, Vitamin K, Arsenic, Boron, Chromium, Copper,	Clinical Effects of Inadequate Intake	Meta-analyses of the results from these and other community-based trials are consistent				

Iodine, Iron, Manganese, Molybdenum, Nickel, Silicon, Vanadium, and Zinc		with a 23 to 30 percent reduction in mortality of young children beyond 6 months of age after vitamin A supplementation				
Scientific Opinion on Dietary Reference Values for vitamin A EFSA Journal 2015;13(3):4028 11	Vitamin A intake and health consequences	A number of intervention studies in children have assessed the effect of vitamin A supplementation on the risk of (premature) death, and the incidence and severity of diarrhoea, measles and lower respiratory tract infections	Vitamin A supplementation in northern Ghana: effects on clinic attendances, hospital admissions, and child mortality. Ghana VAST Study Team [53]	Although most studies on the effect of vitamin A supplementation have reported reductions in childhood mortality, the effects on morbidity are less clear. We have carried out two double-blind, randomised, placebo-controlled trials of vitamin A supplementation in adjacent populations in northern Ghana to assess the impact on childhood morbidity and mortality. The Survival Study included 21,906 children aged 6-90 months in 185 geographical clusters, who were followed for up to 26 months. The Health Study included 1455 children aged 6-59 months, who were monitored weekly for a year. Children were randomly assigned either 200,000 IU retinol equivalent (100,000 IU under 12 months) or placebo every 4 months; randomisation was by individual in the Health Study and by cluster in the Survival Study. There were no significant differences in the Health Study between the vitamin A and placebo groups in the prevalence of diarrhoea or acute respiratory infections; of the symptoms and conditions specifically asked about, only vomiting and anorexia were significantly less frequent in the supplemented children. Vitamin-A-supplemented children had significantly fewer attendances at clinics (rate ratio 0.88 [95% CI 0.81-0.95], p = 0.001), hospital admissions (0.62 [0.42-0.93], p = 0.02), and deaths (0.81 [0.68-0.98], p = 0.03) than children who received placebo. The extent of the effect on morbidity and mortality did not vary significantly with age or sex. However, the mortality rate due to acute gastroenteritis was lower in vitamin-A-supplemented than in placebo clusters (0.66 [0.47-0.92], p = 0.02); mortality rates for all other causes except acute lower respiratory infections and malaria were also lower in vitamin A clusters, but not significantly so. Improving the vitamin A intake of young children in populations where xerophthalmia exists, even at relatively low prevalence, should be a high priority for health and agricultural services in Africa and elsewhere.	2	RCT
Dietary Reference Intakes for Vitamin A, Vitamin K, Arsenic, Boron, Chromium, Copper, Iodine, Iron, Manganese, Molybdenum, Nickel, Silicon, Vanadium, and Zinc	Clinical Effects of Inadequate Intake	In developing countries, vitamin A supplementation has been shown to reduce the risk of mortality among young children				
Scientific Opinion on Dietary Reference Values for vitamin A EFSA Journal 2015;13(3):4028 11	Vitamin A intake and health consequences	A number of intervention studies in children have assessed the effect of vitamin A supplementation on the risk of	Effectiveness of Vitamin A Supplementation in the Control of Young Child Morbidity and Mortality in	No abstract available	1	Systematic review

		(premature) death, and the incidence and severity of diarrhoea, measles and lower respiratory tract infections	Developing Countries. United Nations Administrative Committee on Coordination, Sub-committee on Nutrition State of-the-art Series [54]			
Vitamin and mineral requirements in human nutrition. 2004: World Health Organization	Populations at risk for, and consequences of, vitamin A deficiency	Meta-analyses conducted by three independent groups using data from several randomized trials provide convincing evidence that community-based improvement of the vitamin A status of deficient children aged 6 months to 6 years reduces their risk of dying by 20–30% on average				
Dietary Reference Intakes for Vitamin A, Vitamin K, Arsenic, Boron, Chromium, Copper, Iodine, Iron, Manganese, Molybdenum, Nickel, Silicon, Vanadium, and Zinc	Clinical Effects of Inadequate Intake	Meta-analyses of the results from these and other community-based trials are consistent with a 23 to 30 percent reduction in mortality of young children beyond 6 months of age after vitamin A supplementation				
Scientific Opinion on Dietary Reference Values for vitamin A EFSA	Vitamin A intake and health consequences	A number of intervention studies in children have assessed the	Vitamin A supplementation in infectious	**Objective:** To study the effect of vitamin A supplementation on morbidity and mortality from infectious disease. **Design:** A meta-analysis aimed at identifying and combining mortality and morbidity data from all randomised controlled trials of vitamin A. **Results:** Of 20 controlled trials identified, 12 trials were	1	Systematic review

Journal 2015;13(3):4028 11		effect of vitamin A supplementation on the risk of (premature) death, and the incidence and severity of diarrhoea, measles and lower respiratory tract infections	diseases: a meta-analysis [55]	randomised trials and provided "intention to treat" data: six community trials in developing countries, three in children admitted to hospital with measles, and three in very low birth weight infants. Combined results for community studies suggest a reduction of 30% (95% confidence interval 21% to 38%; two tailed $p < 0.0000001$) in all cause mortality. Analysis of cause specific mortality showed a reduction in deaths from diarrhoeal disease (in community studies) by 39% (24% to 50%; two tailed $p < 0.00001$); from respiratory disease (in measles studies) by 70% (15% to 90%; two tailed $p = 0.02$); and from other causes of death (in community studies) by 34% (15% to 48%; two tailed $p = 0.001$). Reductions in morbidity were consistent with the findings for mortality, but fewer data were available. **Conclusions:** Adequate supply of vitamin A, either through supplementation or adequate diet, has a major role in preventing morbidity and mortality in children in developing countries. In developed countries vitamin A may also have a role in those with life threatening infections such as measles and those who may have a relative deficiency, such as premature infants.		
Vitamin and mineral requirements in human nutrition. 2004: World Health Organization	Populations at risk for, and consequences of, vitamin A deficiency	Meta-analyses conducted by three independent groups using data from several randomized trials provide convincing evidence that community-based improvement of the vitamin A status of deficient children aged 6 months to 6 years reduces their risk of dying by 20–30% on average				
Dietary Reference Intakes for Vitamin A, Vitamin K, Arsenic, Boron, Chromium, Copper, Iodine, Iron, Manganese, Molybdenum, Nickel, Silicon, Vanadium, and Zinc	Clinical Effects of Inadequate Intake	Meta-analyses of the results from these and other community-based trials are consistent with a 23 to 30 percent reduction in mortality of young children beyond 6 months of age after vitamin A supplementation				

Scientific Opinion on Dietary Reference Values for vitamin A EFSA Journal 2015;13(3):4028 11	Vitamin A intake and health consequences	A number of intervention studies in children have assessed the effect of vitamin A supplementation on the risk of (premature) death, and the incidence and severity of diarrhoea, measles and lower respiratory tract infections	Vitamin A for acute respiratory infection in developing countries: a meta-analysis [56]	Aim: To determine the efficacy of intervention with high-dose vitamin A as an adjunct to standard treatment on outcome in acute lower respiratory tract infection in children in developing countries. Methods: A systematic review of double-blinded, randomized, controlled intervention studies of high-dose vitamin A or placebo in children aged between 1 mo and 6 y presenting with acute non-measles lower respiratory tract infection. Results: Five studies fulfilling the criteria were identified and included a total of 2177 children (1067 intervention, 1110 control). The main outcome measures were time to normalization of fever, respiratory rate and oxygen dependence, time to discharge, and mortality. On meta-analysis, there were no significant differences in any of the recovery measures or mortality between the intervention and control groups. Pooled results for recovery times are given showing difference in days to recovery days and 95% confidence intervals. Positive summary measures indicate faster recovery in the vitamin A group and negative in the placebo: fever: 0.03 (-0.10 to 0.17); oxygen requirement: -0.08 (-0.31 to 0.16); raised respiratory rate: -0.09 (-0.38 to 0.19); hospital stay: -0.06 (-0.52 to 0.40). Mortality was below 2% in both groups, with a non-significantly higher risk in the intervention group (odds ratio 1.16, 95% CI: 0.61-2.21). Conclusion: There is no evidence from this meta-analysis that intervention with high-dose vitamin A improves recovery from pneumonia in children in developing countries aged from 1 mo to 6 y.	1	Systematic review
Scientific Opinion on Dietary Reference Values for vitamin A EFSA Journal 2015;13(3):4028 11	Vitamin A intake and health consequences	A number of intervention studies in children have assessed the effect of vitamin A supplementation on the risk of (premature) death, and the incidence and severity of diarrhoea, measles and lower respiratory tract infections	Vitamin A for non-measles pneumonia in children [57]	**Background:** Acute respiratory infections, mostly in the form of pneumonia, are the leading causes of death in children under five years of age in developing countries. Some clinical trials have demonstrated that vitamin A supplementation reduces the severity of respiratory infection and mortality in children with measles. **Objectives:** To determine whether adjunctive vitamin A is effective in infants and children diagnosed with non-measles pneumonia. **Search strategy:** We searched the Cochrane Central Register of Controlled Trials (CENTRAL) (The Cochrane Library Issue 4, 2004); MEDLINE (1996 to November Week 3, 2004); EMBASE (1990 to September 2004); LILACS (9 January 2004); CINAHL (1990 to November 2004); Biological Abstracts (1990 to November 2004) and Current Contents (1990 to September 2004); and the Chinese Biomedicine Database (CBM) (1994 to November 2004). **Selection criteria:** Only parallel-arm, randomised and quasi-randomised controlled trials in which children (younger than 15 years old) with non-measles pneumonia were treated with adjunctive vitamin A were included. **Data collection and analysis:** Two authors independently extracted data and assessed trial quality. Study authors were contacted for additional information. **Main results:** Five trials involving 1453 infants and children were included. There was no significant reduction in the mortality associated with pneumonia in children treated with vitamin A	1	Systematic review

				compared to those who were not (pooled odds ratio (OR) 1.49; 95% confidence interval (CI) 0.66 to 3.35). In addition, there was a lack of a statistically significant effect on duration of stay in hospital (weighted mean difference (WMD) 0.08; 95% CI -0.43 to 0.59). Vitamin A was associated with a 39% reduction in antibiotic firstline failure (OR 0.65; 95% CI 0.42 to 1.01). Children receiving vitamin A were no more likely to experience vomiting (OR 0.77; 95% CI 0.45 to 1.33), diarrhoea (OR 0.57; 95% CI 0.31 to 1.05), bulging of the fontanelles (OR 8.25; 95% CI 0.44 to 155.37) or irritability (OR 0.93, 95% CI 0.56 to 1.57) than those not receiving vitamin A. There was no statistical significance between vitamin A and placebo groups (OR 0.90; 95% CI -1.10 to 2.90) in chest x-ray results. Disease severity after supplementary high-dose vitamin A was significantly worse in children who received vitamin A compared with placebo. Low-dose vitamin A was associated with a significant reduction in the recurrent rate of bronchopneumonia (OR 0.12; 95% CI 0.03 to 0.46). **Authors' conclusions:** The evidence did not suggest a significant reduction with vitamin A adjunctive treatment in mortality, measures of morbidity, nor an effect on the clinical course of pneumonia in children with non-measles pneumonia. However, not all studies measured all outcomes, limiting the number of studies that could be incorporated into the meta-analyses, so that there may have been a lack of statistical power to detect statistically significant differences.		
Scientific Opinion on Dietary Reference Values for vitamin A EFSA Journal 2015;13(3):4028 11	Vitamin A intake and health consequences	A number of intervention studies in children have assessed the effect of vitamin A supplementation on the risk of (premature) death, and the incidence and severity of diarrhoea, measles and lower respiratory tract infections	Impact of vitamin A supplementation on infant and childhood mortality [58]	Introduction: Vitamin A is important for the integrity and regeneration of respiratory and gastrointestinal epithelia and is involved in regulating human immune function. It has been shown previously that vitamin A has a preventive effect on all-cause and disease specific mortality in children under five. The purpose of this paper was to get a point estimate of efficacy of vitamin A supplementation in reducing cause specific mortality by using Child Health Epidemiology Reference Group (CHERG) guidelines. Methods: A literature search was done on PubMed, Cochrane Library and WHO regional data bases using various free and Mesh terms for vitamin A and mortality. Data were abstracted into standardized forms and quality of studies was assessed according to standardized guidelines. Pooled estimates were generated for preventive effect of vitamin A supplementation on all-cause and disease specific mortality of diarrhea, measles, pneumonia, meningitis and sepsis. We did a subgroup analysis for vitamin A supplementation in neonates, infants 1-6 months and children aged 6-59 months. In this paper we have focused on estimation of efficacy of vitamin A supplementation in children 6-59 months of age. Results for neonatal vitamin A supplementation have been presented, however no recommendations are made as more evidence on it would be available soon. Results: There were 21 studies evaluating preventive effect of vitamin A supplementation in community settings which reported all-cause mortality.	1	Systematic review

				Twelve of these also reported cause specific mortality for diarrhea and pneumonia and six reported measles specific mortality. Combined results from six studies showed that neonatal vitamin A supplementation reduced all-cause mortality by 12 % [Relative risk (RR) 0.88; 95 % confidence interval (CI) 0.79-0.98]. There was no effect of vitamin A supplementation in reducing all-cause mortality in infants 1-6 months of age [RR 1.05; 95 % CI 0.88-1.26]. Pooled results for preventive vitamin A supplementation showed that it reduced all-cause mortality by 25% [RR 0.75; 95 % CI 0.64-0.88] in children 6-59 months of age. Vitamin A supplementation also reduced diarrhea specific mortality by 30% [RR 0.70; 95 % CI 0.58-0.86] in children 6-59 months. This effect has been recommended for inclusion in the Lives Saved Tool. Vitamin A supplementation had no effect on measles [RR 0.71, 95% CI: 0.43-1.16], meningitis [RR 0.73, 95% CI: 0.22-2.48] and pneumonia [RR 0.94, 95% CI: 0.67-1.30] specific mortality. Conclusion: Preventive vitamin A supplementation reduces all-cause and diarrhea specific mortality in children 6-59 months of age in community settings in developing countries.		
Scientific Opinion on Dietary Reference Values for vitamin A EFSA Journal 2015;13(3):4028 11	Vitamin A intake and health consequences	A number of intervention studies in children have assessed the effect of vitamin A supplementation on the risk of (premature) death, and the incidence and severity of diarrhoea, measles and lower respiratory tract infections	Mortality and morbidity, especially in relation to infections [59]	*No abstract available*		Non-systematic review
Scientific Opinion on Dietary Reference Values for vitamin A EFSA Journal 2015;13(3):4028 11	Vitamin A intake and health consequences	Some observational studies have assessed the association between "vitamin A" or retinol intake and asthma, wheeze or other measures of lung	Nutrients and foods for the primary prevention of asthma and allergy: systematic review and meta-analysis [60]	Background: Epidemiologic studies suggest that deficiencies of the nutrients selenium; zinc; vitamins A, C, D, and E; and low fruit and vegetable intake may be associated with the development of asthma and allergic disorders. Objectives: To investigate the evidence that nutrient and food intake modifies the risk of children developing allergy. Methods: We systematically searched 11 databases. Studies were critically appraised, and meta-analyses were undertaken. Results: We identified 62 eligible reports. There were no randomized controlled trials. Studies used cohort (n = 21), case-control (n = 15), or cross-sectional (n = 26) designs. All studies were judged to be at moderate to substantial risk of bias.	1	Systematic review

		function with inconsistent results		Meta-analysis revealed that serum vitamin A was lower in children with asthma compared with controls (odds ratio [OR], 0.25; 95% CI, 0.10-0.40). Meta-analyses also showed that high maternal dietary vitamin D and E intakes during pregnancy were protective for the development of wheezing outcomes (OR, 0.56, 95% CI, 0.42-0.73; and OR, 0.68, 95% CI, 0.52-0.88, respectively). Adherence to a Mediterranean diet was protective for persistent wheeze (OR, 0.22; 95% CI, 0.08-0.58) and atopy (OR, 0.55; 95% CI, 0.31-0.97). Seventeen of 22 fruit and vegetable studies reported beneficial associations with asthma and allergic outcomes. Results were not supportive for other allergic outcomes for these vitamins or nutrients, or for any outcomes in relation to vitamin C and selenium. Conclusion: The available epidemiologic evidence is weak but nonetheless supportive with respect to vitamins A, D, and E; zinc; fruits and vegetables; and a Mediterranean diet for the prevention of asthma. Experimental studies of these exposures are now warranted.		
Scientific Opinion on Dietary Reference Values for vitamin A EFSA Journal 2015;13(3):4028 11	Retinol equivalents – conversion factors	Ribaya-Mercado et al. showed a significant improvement in vitamin A status, as assessed by the deuterated retinol dilution method, in Filipino schoolchildren receiving controlled diets rich in provitamin A carotenoids from fruit and vegetable sources, but these studies do not allow the estimation of provitamin A carotenoid/retinol equivalency ratios	Bioconversion of plant carotenoids to vitamin A in Filipino school-aged children varies inversely with vitamin A status [61]	Background: It is important to understand the factors affecting strategies to improve the vitamin A status of populations. We reported previously that a 3-d deuterated-retinol-dilution (DRD) procedure might be used to indicate total body stores of vitamin A. Objective: We studied the ability of 3-d DRD to detect changes in the body pool size of vitamin A and the effect of vitamin A status on the bioconversion of plant carotenoids to vitamin A. Design: Two separate, unrelated studies were conducted in 7–13-y-old children with poor or marginal serum retinol concentrations (0.32–0.93 µmol/L) by feeding them controlled diets daily for 5 d/wk for 12 wk, after treatment with an anthelmintic drug. In school 1 (n = 27), lunch and 2 snacks that were provided at school contained 2258 retinol equivalents/d (mostly from orange fruit and vegetables) and 5.3 MJ/d from 33 g fat, 37 g protein, and 209 g carbohydrates; in school 2 (n = 25), 2 snacks provided 2.5 MJ/d from 9.4 g fat, 9.6 g protein, and 119 g carbohydrates, but no carotenes. Results: In school 1, mean serum β-carotene increased from 0.12 to 0.62 µmol/L (P = 0.0001) and serum retinol increased from 0.68 to 1.06 µmol/L (P = 0.0001). In school 2, serum β-carotene increased from 0.06 to 0.11 µmol/L (P = 0.0001) and serum retinol increased from 0.66 to 0.86 µmol/L (P = 0.0001). In school 1, but not school 2, improvement in serum retinol varied inversely with baseline retinol (r = −0.38, P = 0.048). In both schools, 3-d DRD showed reductions in the ratio of serum deuterated to nondeuterated retinol (D:H retinol) postintervention, denoting improvements in vitamin A status; the higher D:H retinol (ie, the poorer the status) at baseline, the greater the reduction in D:H retinol postintervention (school 1: r = −0.99, P = 0.0001; school 2: r = −0.89, P = 0.0001). Conclusions: Three-day DRD can detect changes in the body pool size of vitamin A, although a predictive equation to quantitate total body stores of vitamin A with the use of 3-d data needs to be developed.	4	Experi mental - other

				Bioconversion of plant carotenoids to vitamin A varies inversely with vitamin A status; improvement in status after dietary interventions is strongly influenced by total body stores of vitamin A and is influenced little or not at all by serum retinol.		
Scientific Opinion on Dietary Reference Values for vitamin A EFSA Journal 2015;13(3):4028 11	Retinol equivalents – conversion factors	Ribaya-Mercado et al. showed a significant improvement in vitamin A status, as assessed by the deuterated retinol dilution method, in Filipino schoolchildren receiving controlled diets rich in provitamin A carotenoids from fruit and vegetable sources, but these studies do not allow the estimation of provitamin A carotenoid/retinol equivalency ratios	Carotene-rich plant foods ingested with minimal dietary fat enhance the total-body vitamin A pool size in Filipino schoolchildren as assessed by stable-isotope-dilution methodology [62]	**Background:** Strategies for improving the vitamin A status of vulnerable populations are needed. **Objective:** We studied the influence of the amounts of dietary fat on the effectiveness of carotene-rich plant foods in improving vitamin A status. **Design:** Schoolchildren aged 9-12 y were fed standardized meals 3 times/d, 5 d/wk, for 9 wk. The meals provided 4.2 mg provitamin A carotenoids/d (mainly beta-carotene) from yellow and green leafy vegetables [carrots, pechay (bok choy), squash, and kangkong (swamp cabbage)] and 7, 15, or 29 g fat/d (2.4, 5, or 10 g fat/meal) in groups A, B, and C (n = 39, 39, and 38, respectively). Other self-selected foods eaten were recorded daily. Before and after the intervention, total-body vitamin A pool sizes and liver vitamin A concentrations were measured with the deuterated-retinol-dilution method; serum retinol and carotenoid concentrations were measured by HPLC. **Results:** Similar increases in mean serum beta-carotene (5-fold), alpha-carotene (19-fold), and beta-cryptoxanthin (2-fold) concentrations; total-body vitamin A pool size (2-fold); and liver vitamin A (2-fold) concentrations were observed after 9 wk in the 3 study groups; mean serum retinol concentrations did not change significantly. The total daily beta-carotene intake from study meals plus self-selected foods was similar between the 3 groups and was 14 times the usual intake; total fat intake was 0.9, 1.4, or 2.0 times the usual intake in groups A, B, and C, respectively. The overall prevalence of low liver vitamin A (<0.07 mumol/g) decreased from 35% to 7%. **Conclusions:** Carotene-rich yellow and green leafy vegetables, when ingested with minimal fat, enhance serum carotenoids and the total-body vitamin A pool size and can restore low liver vitamin A concentrations to normal concentrations.	2	RCT
Scientific Opinion on Dietary Reference Values for vitamin A EFSA Journal 2015;13(3):4028 11	Retinol equivalents – conversion factors	Few results are available on the rate of absorption of β-carotene and its bioequivalence to retinol in children	A review of vitamin A equivalency of beta carotene in various food matrices for human consumption [63]	Vitamin A equivalency of β-carotene (VEB) is defined as the amount of ingested β-carotene in µg that is absorbed and converted into 1 µg retinol (vitamin A) in the human body. The objective of the present review was to discuss the different estimates for VEB in various types of dietary food matrices. Different methods are discussed such as mass balance, dose–response and isotopic labelling. The VEB is currently estimated by the US Institute of Medicine (IOM) as 12:1 in a mixed diet and 2:1 in oil. For humans consuming β-carotene dissolved in oil, a VEB between 2:1 and 4:1 is feasible. A VEB of approximately 4:1 is applicable for biofortified cassava, yellow maize and Golden Rice, which are specially bred for human consumption in developing countries. We propose a	6	Non-systematic review

				range of 9:1–16:1 for VEB in a mixed diet that encompasses the IOM VEB of 12:1 and is realistic for a Western diet under Western conditions. For a 'prudent' (i.e. non-Western) diet including a variety of commonly consumed vegetables, a VEB could range from 9:1 to 28:1 in a mixed diet.		
Vitamin and mineral requirements in human nutrition. 2004: World Health Organization	Populations at risk for, and consequences of, vitamin A deficiency	VAD related blindness is most prevalent in children under 3 years of age	Vitamin A deficiency and its consequences: a field guide to detection and control [64]	No abstract available.	6	Non-systematic review
Vitamin and mineral requirements in human nutrition. 2004: World Health Organization	Populations at risk for, and consequences of, vitamin A deficiency	There is little information regarding the health consequences of VAD in school-age children. The prevalence of Bitot's spots (i.e. white foamy patches on the conjunctiva) may be highest in this age group but their occurrence may reflect past more than current history of VAD	Clinical characteristics of vitamin A responsive and nonresponsive Bitot's spots [65]	No abstract available.	5	Observational
Vitamin and mineral requirements in human nutrition. 2004: World Health Organization	Populations at risk for, and consequences of, vitamin A deficiency	..breast milk is frequently low in vitamin A	Vitamin A and breast-feeding: a comparison of data from developed and developing countries [66]	The vitamin A status of lactating women, its effect on the vitamin A content of human milk, and the adequacy of human milk as a source of vitamin A for the infant were assessed, comparing data from developing countries with those from developed countries. The vitamin A concentration in breast milk during the first two weeks of lactation is nearly double that at one month. It is even higher in preterm milk than in term milk during the first several months. Human milk alone provides sufficient vitamin A to prevent clinical deficiency throughout the first 12 months of life, even in presumably more poorly nourished populations in developing countries. However, it is not sufficient to allow liver storage after about six months of lactation.	6	Non-systematic review

Vitamin and mineral requirements in human nutrition. 2004: World Health Organization	Populations at risk for, and consequences of, vitamin A deficiency	Growth rates, and presumably the need for vitamin A, from birth to 10 years for boys are consistently higher than those for girls	Physical status: the use and interpretation of anthropometry. Report of a WHO Expert Committee [67]	No abstract available		Published guidelines
Vitamin and mineral requirements in human nutrition. 2004: World Health Organization	Populations at risk for, and consequences of, vitamin A deficiency	Seasonal growth spurts in children, which frequently follow seasonal post-harvest increases in energy and macronutrient intakes, can also affect the balance. These increases are usually obtained from staple grains (e.g. rice) and tubers (e.g. lightcoloured yams) that are not, however, good sources of some micronutrients (e.g. vitamin A) to support the growth spurt	Seasonal variation in signs of vitamin A deficiency in rural West Bengal children [68]	Marked variation in the seasonal prevalence of the signs of vitamin-A deficiency was apparent in a village of West Bengal over a period of a year and a half. This was demonstrated in a group of 312 children (aged 0-4 years) who were continuously studied by weekly morbidity history and monthly physical examinations. They showed two definite peaks of vitamin-A deficiency—one in November-December and the other in May-June. The two sexes did not differ significantly. The rise and fall in the prevalence does not seem to be fully explained by variation in intake of vitamin A. Possibly this variation is also associated with seasonal variation in growth, related to total food intake.	5	Observational
Vitamin and mineral requirements in human nutrition. 2004: World Health Organization	Populations at risk for, and consequences of, vitamin A deficiency	Food habits and taboos often restrict consumption of potentially good food sources of vitamin A (e.g. mangoes and green leafy vegetables). Culture specific	Nutritional and household risk factors for xerophthalmia in Aceh, Indonesia: a case–control study [69]	Risk factors for xerophthalmia were assessed in 466 subjects [38% with night blindness (XN), 60% with Bitot's spots (X1B), 2% with corneal xerophthalmia (X2 or X3)] under age 6 y and their village-age-sex-matched control subjects during a community trial. Socioeconomic status and hygiene standards were lowest for households of xerophthalmic children and highest for nonstudy households in the trial population, with values for control households lying in between (P less than 0.001 by linear trend). Risk of xerophthalmia increased with less frequent consumption of dark green leaves, yellow fruits, or egg during weaning, adjusted for current intake and present age [odds ratio (OR) = approximately 3.5]. Exclusion of these same foods from the current diet (except for mango and	5	Observational

		factors for feeding children, adolescents, and pregnant and lactating women are common		papaya in older children) was associated with a two- to ninefold excess risk of xerophthalmia, adjusted for weaning influences. Xerophthalmic children aged less than 3 y were generally at higher risk of dietary imbalance than were older children. Xerophthalmia is associated with a chronic, infrequent consumption of key vitamin A foods from weaning through early childhood.		
Vitamin and mineral requirements in human nutrition. 2004: World Health Organization	Populations at risk for, and consequences of, vitamin A deficiency	Food habits and taboos often restrict consumption of potentially good food sources of vitamin A (e.g. mangoes and green leafy vegetables). Culture specific factors for feeding children, adolescents, and pregnant and lactating women are common	Dietary practices and xerophthalmia among Indonesian Children [70]	The stated frequency with which 30 Indonesian children with corneal xerophthalmia and age/sex/neighborhood matched controls ordinarily consumed vitamin- and provitamin A-rich foods was compared. Controls were more frequent consumers of eggs (p less than 0.05), fish (p less than 0.05), dark green leafy vegetables (p less than 0.05), carrots (p less than 0.01), and carotene-containing fruits (p less than 0.1). Similar data were collected on 358 children with Bitot's spots and on normal preschool age children in a countrywide survey. Breast-feeding was more common among normals than among cases (p less than 0.001). Normals were also more frequent consumers of mango and papaya during the 2nd and 3rd yr of life (p less than 0.05); and of dark green leafy vegetables and eggs during the 3rd through 6th yr of life (p less than 0.01). In two separate studies, differences in carotene consumption by normals and abnormals were confirmed by differences in their serum carotene levels. Approximately 80% of Indonesian families, with an without xerophthalmic children, consumed dark-green leafy vegetables at least once a day, and 99% at least once a week. Diet therefore appears to be an important factor in the genesis of xerophthalmia in Indonesia despite the availability of suitable provitamin A-rich foods.	5	Observ ational
Vitamin and mineral requirements in human nutrition. 2004: World Health Organization	Populations at risk for, and consequences of, vitamin A deficiency	Food habits and taboos often restrict consumption of potentially good food sources of vitamin A (e.g. mangoes and green leafy vegetables). Culture specific factors for feeding children, adolescents, and pregnant and lactating	Mothers' and children's intakes of vitamin A in rural Bangladesh [71]	The vitamin A intake of 370 mothers and 183 children 3-27 mo of age in rural Bangladesh was ascertained monthly from January to July in 1986. For mothers, dark green leafy vegetables and fruits were the main sources of vitamin A. Vitamin A from vegetables, the single most important source, did not show consistent associations with wealth or with the other socioeconomic indicators. In May and June, fruits provided wealthier (and more educated) mothers with significantly higher vitamin A intakes than poor mothers, whereas in January the poorer mothers had higher intakes. When breast milk was included, average intakes for children came close to 100% of the recommended dietary allowance; the only other significant source of vitamin A for children was seasonally available mangoes. Fourteen children who had stopped breast-feeding by the end of the study were at very high risk of vitamin A deficiency when fruits were not plentiful.	5	Observ ational

		women are common				
Vitamin and mineral requirements in human nutrition. 2004: World Health Organization	Populations at risk for, and consequences of, vitamin A deficiency	In young children, subclinical deficiency, like clinical deficiency, increases the severity of some infections, particularly diarrhoea and measles, and increases the risk of death	Vitamin A supplementation in northern Ghana: effects on clinic attendance, hospital admissions, and child mortality [72]	Although most studies on the effect of vitamin A supplementation have reported reductions in childhood mortality, the effects on morbidity are less clear. We have carried out two double-blind, randomised, placebo-controlled trials of vitamin A supplementation in adjacent populations in northern Ghana to assess the impact on childhood morbidity and mortality. The Survival Study included 21,906 children aged 6-90 months in 185 geographical clusters, who were followed for up to 26 months. The Health Study included 1455 children aged 6-59 months, who were monitored weekly for a year. Children were randomly assigned either 200,000 IU retinol equivalent (100,000 IU under 12 months) or placebo every 4 months; randomisation was by individual in the Health Study and by cluster in the Survival Study. There were no significant differences in the Health Study between the vitamin A and placebo groups in the prevalence of diarrhoea or acute respiratory infections; of the symptoms and conditions specifically asked about, only vomiting and anorexia were significantly less frequent in the supplemented children. Vitamin-A-supplemented children had significantly fewer attendances at clinics (rate ratio 0.88 [95% CI 0.81-0.95], p = 0.001), hospital admissions (0.62 [0.42-0.93], p = 0.02), and deaths (0.81 [0.68-0.98], p = 0.03) than children who received placebo. The extent of the effect on morbidity and mortality did not vary significantly with age or sex. However, the mortality rate due to acute gastroenteritis was lower in vitamin-A-supplemented than in placebo clusters (0.66 [0.47-0.92], p = 0.02); mortality rates for all other causes except acute lower respiratory infections and malaria were also lower in vitamin A clusters, but not significantly so. Improving the vitamin A intake of young children in populations where xerophthalmia exists, even at relatively low prevalence, should be a high priority for health and agricultural services in Africa and elsewhere.	2	RCT
Vitamin and mineral requirements in human nutrition. 2004: World Health Organization	Populations at risk for, and consequences of, vitamin A deficiency	The incidence and prevalence of diarrhoea may also increase with subclinical VAD.	Effect of vitamin A supplementation on diarrhoea and acute lower-respiratory-tract infections in young children in Brazil [73]	A beneficial effect of periodic vitamin A supplementation on childhood mortality has been demonstrated, but the effect on morbidity is less clear. We investigated the effect of vitamin A supplementation on diarrhoea and acute lower-respiratory-tract infections (ALRI) in children from northeastern Brazil in a randomised, double-blind, placebo-controlled community trial. 1240 children aged 6-48 months were assigned vitamin A or placebo every 4 months for 1 year. They were followed up at home three times a week, and data about the occurrence and severity of diarrhoea and ALRI were collected. Any child with cough and respiratory rate above 40 breaths per min was visited by a paediatrician. The overall incidence of diarrhoea episodes was significantly lower in the vitamin-A-supplemented group than in the placebo group (18.42 vs 19.58	2	RCT
Dietary Reference Intakes for Vitamin A, Vitamin K, Arsenic, Boron, Chromium, Copper,	Clinical Effects of Inadequate Intake	In some studies, vitamin A supplementation (30 to 60 mg) has been shown to				

Iodine, Iron, Manganese, Molybdenum, Nickel, Silicon, Vanadium, and Zinc		reduce the severity of diarrhea		x 10(-3) child-days; rate ratio 0.94 [95% Cl 0.90-0.98]). The benefit of supplementation was greater as regards severe episodes of diarrhoea; the incidence was 20% lower in the vitamin A group than in the placebo group (rate ratio 0.80 [0.65-0.98]). With the standard definition of diarrhoea (> or = 3 liquid or semi-liquid stools in 24 h) the effect of vitamin A on mean daily prevalence did not reach significance, but as the definition of diarrhoea was made more stringent (increasing number of stools per day), a significant benefit became apparent, reaching for diarrhoea with 6 or more liquid or semi-liquid stools in 24 h a 23% lower prevalence. We found no effect of vitamin A supplementation on the incidence of ALRI. The reduction in severity of diarrhoea may be the most important factor in the lowering of mortality by vitamin A supplementation.		
Vitamin and mineral requirements in human nutrition. 2004: World Health Organization	Populations at risk for, and consequences of, vitamin A deficiency	The incidence and prevalence of diarrhoea may also increase with subclinical VAD.	Impact of massive dose of vitamin A given to preschool children with acute-diarrhoea on subsequent respiratory and diarrhoeal morbidity [74]	Objective: To assess the impact of vitamin A supplementation on morbidity from acute respiratory tract infections and diarrhoea.<br>Design: Double blind randomised placebo controlled field trial.<br>Setting: An urban slum area in New Delhi, India.<br>Subjects: 900 children aged 12-60 months attending a local health facility for acute diarrhoea of less than seven days' duration randomly allocated to receive vitamin A 200,000 IU or placebo.<br>Main outcome measures: Incidence and prevalence of acute lower respiratory tract infections and diarrhoea during the 90 days after termination of the enrolment diarrhoeal episode measured by twice weekly household surveillance.<br>Results: The incidence (relative risk 1.07; 95% confidence interval 0.92 to 1.26) and average number of days spent with acute lower respiratory tract infections were similar in the vitamin A supplementation and placebo groups. Among children aged 23 months or less there was a significant reduction in the incidence of measles (relative risk 0.06; 95% confidence interval 0.01 to 0.48). The incidence of diarrhoea was also similar (relative risk 0.95; 0.86 to 1.05) in the two groups. There was a 36% reduction in the mean daily prevalence of diarrhoea associated with fever in the vitamin A supplemented children older than 23 months.<br>Conclusions: Results were consistent with a lack of impact on acute lower respiratory tract related mortality after vitamin A supplementation noted in other trials and a possible reduction in the severity of diarrhoea.	2	RCT
Vitamin and mineral requirements in human nutrition. 2004: World Health Organization	Populations at risk for, and consequences of, vitamin A deficiency	Meta-analyses conducted by three independent groups using data from several	Vitamin A supplementation and child mortality [75]	Objective: A two-part meta-analysis of studies examining the relationship of vitamin A supplementation and child mortality.<br>Data sources: We identified studies by searching the MEDLARS database from 1966 through 1992 and by scanning Current Contents and bibliographies of pertinent articles.	1	System atic review

		randomized trials provide convincing evidence that community-based improvement of the vitamin A status of deficient children aged 6 months to 6 years reduces their risk of dying by 20–30% on average		Study selection: All 12 vitamin A controlled trials with data on mortality identified in the search were used in the analysis.<br>Data extraction: Data were independently extracted by two investigators who also assessed the quality of each study using a previously described method.<br>Data synthesis: We formally tested for heterogeneity across studies. We pooled studies using the Mantel-Haenszel and the DerSimonian and Laird methods and adjusted for the effect of cluster assignment of treatment groups in community-based studies. Vitamin A supplementation to hospitalized measles patients was highly protective against mortality (DerSimonian and Laird odds ratio, 0.39; 95% confidence interval, 0.22 to 0.66; P = .0004) (part 1 of the meta-analysis). Supplementation was also protective against overall mortality in community-based studies (DerSimonian and Laird odds ratio, 0.70; clustering-adjusted 95% confidence interval, 0.56 to 0.87; P = .001) (part 2 of the meta-analysis).<br>Conclusions: Vitamin A supplements are associated with a significant reduction in mortality when given periodically to children at the community level. Factors that affect the bioavailability of large doses of Vitamin A need to be studied further. Vitamin A supplements should be given to all measles patients in developing countries whether or not they have symptoms of vitamin A deficiency.		
Vitamin and mineral requirements in human nutrition. 2004: World Health Organization	Populations at risk for, and consequences of, vitamin A deficiency	Mortality in children who are blind from keratomalacia or who have corneal disease is reported to be from 50% to 90%	Sequelae of severe xerophthalmia: a follow-up study [76]	Two-hundred sixteen children with vitamin A deficiency were hospitalized in Hyderabad, India, between 1970 and 1977. Out of these, 22 died during hospitalization. Fifty-six children could be followed-up, of whom 32 had keratomalacia and 24 corneal xerosis. In the keratomalacia group, nine died of reasons attributable to severe protein-energy malnutrition within 3 to 4 months after discharge from the hospital, five became totally blind, nine monocularly blind, and nine had adequate vision, although four of these had leucomas. Of the blind children, no change in condition was noted in the 11 located 1 year after the initial 3 to 4 month follow-up period. Of the 24 children with corneal zerosis, two died within 3 months, 21 retained normal vision, and one had adequate vision with leucoma. The high death rate, i.e., 30% of the keratomalacia cases in this study helps to explain the low prevalence of keratomalacia observed in past and current community surveys. The high return to normalcy of vision in the corneal xerosis cases after proper treatment support the recommendation of treating all children with eye involvement before the keratomalacia state whenever possible, for greater assurance of subsequent eye improvement.	5	Observational
Vitamin and mineral requirements in human nutrition.	Populations at risk for, and consequences of, vitamin A deficiency	measles mortality associated with VAD is increased by up to 50%	A randomized controlled trial of vitamin A in children with severe measles [77]	Background: Measles kills about 2 million children annually, and there is no specific therapy for the disease. It has been suggested that vitamin A may be of benefit in the treatment of measles.<br>Methods: We conducted a randomized, double-blind trial involving 189 children who were hospitalized at a regional center in South Africa because of measles	2	RCT

2004: World Health Organization<br>Dietary Reference Intakes for Vitamin A, Vitamin K, Arsenic, Boron, Chromium, Copper, Iodine, Iron, Manganese, Molybdenum, Nickel, Silicon, Vanadium, and Zinc	Clinical Effects of Inadequate Intake	In children hospitalized with measles, case fatality… were reduced when they received high doses (60 to 120 mg) of vitamin A.		complicated by pneumonia, diarrhea, or croup. The children (median age, 10 months) were assigned to receive either vitamin A (total dose, 400,000 IU of retinyl palmitate, given orally; n = 92) or placebo (n = 97), beginning within five days of the onset of the rash. At base line, the characteristics of the two groups were similar.<br>Results: Although clinically apparent vitamin A deficiency is rare in this population, the children's serum retinol levels were markedly depressed (mean [+/- SEM], 0.405 +/- 0.021 mumols per liter [11.6 +/- 0.6 micrograms per deciliter]), and 92 percent of them had hyporetinemia (serum retinol level less than 0.7 mumols per liter [20 micrograms per deciliter]). Serum concentrations of retinol-binding protein (mean, 30.1 +/- 2.0 mg per liter) and albumin (mean, 33.4 +/- 0.5 g per liter) were also low. As compared with the placebo group, the children who received vitamin A recovered more rapidly from pneumonia (mean, 6.3 vs. 12.4 days, respectively; P less than 0.001) and diarrhea (mean, 5.6 vs. 8.5 days; P less than 0.001), had less croup (13 vs. 27 cases; P = 0.03), and spent fewer days in the hospital (mean, 10.6 vs. 14.8 days; P = 0.01). Of the 12 children who died, 10 were among those given placebo (P = 0.05). For the group treated with vitamin A, the risk of death or a major complication during the hospital stay was half that of the control group (relative risk, 0.51; 95 percent confidence interval, 0.35 to 0.74).<br>Conclusions: Treatment with vitamin A reduces morbidity and mortality in measles, and all children with severe measles should be given vitamin A supplements, whether or not they are thought to have a nutritional deficiency.		
Vitamin and mineral requirements in human nutrition. 2004: World Health Organization	Populations at risk for, and consequences of, vitamin A deficiency	A great deal of evidence supports an association of VAD with severity of an infection once acquired, except for respiratory diseases, which are non-responsive to treatment	Potential interventions for the prevention of childhood pneumonia in developing countries: a metaanalysis of data from field trials to assess the impact of vitamin A supplementation on pneumonia morbidity and mortality [78]	Reported are the results of a meta-analysis (12 large-scale field trials in seven countries) of the impact of vitamin A supplementation on pneumonia morbidity and mortality, undertaken as part of a wider review process of a range of possible potential interventions for the prevention of childhood pneumonia. The summary estimate of the relative risk for the impact of vitamin A supplementation on pneumonia incidence was 0.95 (95% confidence interval (CI) = 0.89, 1.01), and for pneumonia mortality, 0.98 (95% CI = 0.75, 1.28). This is in marked contrast to the substantial impact of vitamin A supplementation on all-cause mortality (combined rate ratio (RR) = 0.77, 95% CI = 0.71, 0.84), and on diarrhoea-specific and measles-specific mortality. There was no evidence for a differential impact on pneumonia mortality by age. Since the majority of pneumonia deaths occur in the first year of life, we complemented the paucity of data on pneumonia-specific mortality among this age group with a detailed examination of all-cause mortality among infants. The mortality reduction in the 6-11 month age group was consistent with that observed for older age groups (RR = 0.69; 95% CI = 0.54, 0.90), but there was no reduction for 0-5 month-olds (RR = 0.97; 95% CI = 0.73, 1.29).	1	Systematic review

Vitamin and mineral requirements in human nutrition. 2004: World Health Organization	Populations at risk for, and consequences of, vitamin A deficiency	The severity of pneumonia associated with measles, however, is an exception because it decreases with the treatment of vitamin A supplementation	Vitamin A supplementation reduces measles morbidity in young African children: a randomized, placebo-controlled, double blind trial [79]	The effects of vitamin A supplementation on measles morbidity are unclear. Sixty hospitalized children aged 4-24 mo with complicated measles received a World Health Organization--(WHO) recommended dose of vitamin A or placebo. The two groups were comparable in known covariants of measles severity: weight-for-age percentiles, overcrowding, rash, total lymphocytes, and serum concentrations of zinc, albumin, prealbumin, retinol-binding protein, and vitamins A and E. Ninety percent of the patients had hyporetinemia. Integrated morbidity scores, determined by severity of condition (eg, diarrhoea, herpes, and respiratory-tract infection) were assigned on day 8 and 6 wk and 6 mo; these were reduced by 82%, 61%, and 85%, respectively, in the supplemented group, which was mainly due to reduced respiratory-tract infection. There was one death in the placebo group. At 6 wk weight gain was significant in the supplemented group. Despite the selected sample, attention to multiple covariates enhances the validity of the data obtained and supports the current WHO recommendations for vitamin A supplementation during measles.	2	RCT
Dietary Reference Intakes for Vitamin A, Vitamin K, Arsenic, Boron, Chromium, Copper, Iodine, Iron, Manganese, Molybdenum, Nickel, Silicon, Vanadium, and Zinc	Clinical Effects of Inadequate Intake	In children hospitalized with measles, case fatality… were reduced when they received high doses (60 to 120 mg) of vitamin A.				
Vitamin and mineral requirements in human nutrition. 2004: World Health Organization	Populations at risk for, and consequences of, vitamin A deficiency	Infectious diseases depress circulating retinol and contribute to vitamin A depletion. Enteric infections may alter the absorptive surface area, compete for absorption-binding sites, and increase urinary loss	Vitamin A deficiency and diarrhoea: a review of interrelationships and their implications for the control of xerophthalmia and diarrhoea [80]	No abstract available	6	Non-systematic review
Vitamin and mineral requirements in human nutrition. 2004: World Health Organization	Populations at risk for, and consequences of, vitamin A deficiency	metabolic utilization rates and may reduce apparent retinol stores if fever occurs frequently	Effect of an infection on vitamin A status of children as measured by the	The effect of an infective episode of chickenpox on the vitamin A status of preschool-aged children was evaluated by use of the relative dose response (RDR) test. Status was determined before and 30, 120, and 180 d after administration of a single oral high-dosage (200,000 IU) supplement of vitamin A. No differences in mean blood levels of retinol or percentage of children showing a positive RDR were apparent until after the infective episode that	5	Observational

			relative dose response (RDR) [81]	occurred approximately 90 d after dosing. At 180 d postsupplementation, 74% of children who had been infected tested positive by the RDR, indicative of an inadequate liver reserve of vitamin A, in contrast to only 10% who had not been infected. Paired RDR observations at 0 and 180 d postsupplementation confirmed that the infective episode caused an accelerated depletion of liver reserves of vitamin A.		
Vitamin and mineral requirements in human nutrition. 2004: World Health Organization	Populations at risk for, and consequences of, vitamin A deficiency	Measles virus infection is especially devastating to vitamin A metabolism, adversely interfering with both efficiencies of utilization and conservation	Corneal ulceration in Tanzanian children: relationship between measles and vitamin A deficiency [82]	Two 3-year prospective studies from 1982-1984 and 1986-1988 in Tanzania identified 189 children with corneal ulceration, of whom 31 (16.4%) were due to vitamin A deficiency. In 1982-1984, vitamin A deficiency was responsible for 23.4% of corneal ulcers (25 of 107) compared with 7.3% (6 of 82) from 1986-1988 (P = 0.006). It is postulated that the decrease in corneal ulceration due to vitamin A deficiency can be attributed to improved measles immunization coverage.	5	Observational
Vitamin and mineral requirements in human nutrition. 2004: World Health Organization	Populations at risk for, and consequences of, vitamin A deficiency	Severe protein–energy malnutrition affects many aspects of vitamin A metabolism, and even when some retinyl ester stores are still present, malnutrition—often coupled with infection—can prevent transport-protein synthesis, resulting in immobilization of existing vitamin A stores	Serum and liver vitamin A and lipids in children with severe protein malnutrition [83]	The serum levels of total lipids, phospholipids, cholesterol, vitamin A and carotene were measured in children with kwashiorkor on admission to the hospital, and at regular intervals during the first weeks of recovery. Marked increases in all these serum constituents were observed, except for vitamin A in some of the children. Liver biopsy specimens were also studied for the same lipid constituents, except cholesterol. The data indicate that total lipids and vitamin A decrease in liver tissue simultaneously with an increase in these serum components. The findings are suggestive of an initial impairment in lipid and vitamin A blood transport possibly associated with decreases in the plasma protein fractions to which these lipid compounds are normally bound. Phospholipids differ in that they are apparently synthesized in the liver at a rate sufficient to prevent their decrease in hepatic tissue despite increased removal in the circulation.	5	Observational
Vitamin and mineral requirements in human nutrition. 2004: World Health Organization	Units of expression	Recently there has been renewed interest in re-examining conventional	Green and yellow vegetables can maintain body stores of vitamin A in Chinese children [84]	Background: Vitamin A activity of plant provitamin A carotenoids is uncertain.<br>Objective: The objective was to determine whether plant carotenoids can sustain or improve vitamin A nutrition during the fall season in kindergarten children in the Shandong province of China.<br>Design: The serum vitamin A concentration of 39% of the children was <1.05 micromol/L and of 61% of the children was > or = 1.05 micromol/L. For 5 d/wk	3d	Experimental - stores

		conversion factors by using more quantitative stable isotope techniques for measuring whole-body stores in response to controlled intakes		for 10 wk, 22 children were provided approximately 238 g green-yellow vegetables/d and 34 g light-colored vegetables/d. Nineteen children maintained their customary dietary intake, which included 56 g green-yellow vegetables/d and 224 g light-colored vegetables/d. Octadeuterated and tetradeuterated vitamin A were given before and after the interventions, respectively, and their enrichments in the plasma were determined by gas chromatography-mass spectrometry. Serum retinol and carotenoid concentrations were measured by HPLC.<br>Results: Carotenoid nutrition improved after consumption of green-yellow vegetables. Serum concentrations of retinol were sustained in the group fed green-yellow vegetables but decreased in the group fed light-colored vegetables ($P < 0.01$). The isotope-dilution tests confirmed that total-body vitamin A stores were sustained in the group fed green-yellow vegetables, but decreased 27 micromol (7700 microg retinol) per child, on average, in the group fed light-colored vegetables ($P < 0.06$).<br>Conclusion: Green-yellow vegetables can provide adequate vitamin A nutrition in the diet of kindergarten children and protect them from becoming vitamin A deficient during seasons when the provitamin A food source is limited.		
Vitamin and mineral requirements in human nutrition. 2004: World Health Organization	Sources and supply patterns of vitamin A	Nutrient intakes presented.	Food and nutrient intakes by individuals in the United States, by sex and age 1994–96 [85]	No abstract available		Published guidelines
Vitamin and mineral requirements in human nutrition. 2004: World Health Organization	Sources and supply patterns of vitamin A	Nutrient intakes presented.	National Health and Nutrition Examination Survey III, 1988–1994 [86]	No abstract available		Published guidelines
Vitamin and mineral requirements in human nutrition. 2004: World Health Organization	Sources and supply patterns of vitamin A	Most studies report a positive response when vegetable sources of provitamin A are given under controlled conditions to deficient subjects freed of	Green and yellow vegetables rich in provitamin A carotenoids can sustain vitamin A status in children [87]	Background: Vitamin A activity of plant provitamin A carotenoids is uncertain.<br>Objective: The objective was to determine whether plant carotenoids can sustain or improve vitamin A nutrition during the fall season in kindergarten children in the Shandong province of China.<br>Design: The serum vitamin A concentration of 39% of the children was <1.05 µmol/L and of 61% of the children was ≥1.05 µmol/L. For 5 d/wk for 10 wk, 22 children were provided ≈238 g green-yellow vegetables/d and 34 g light-colored vegetables/d. Nineteen children maintained their customary dietary intake, which included 56 g green-yellow vegetables/d and 224 g light-colored vegetables/d. Octadeuterated and tetradeuterated vitamin A were given before	3d	Experimental - stores

		confounding parasite loads and provided with sufficient dietary fat		and after the interventions, respectively, and their enrichments in the plasma were determined by gas chromatography–mass spectrometry. Serum retinol and carotenoid concentrations were measured by HPLC.<br>Results: Carotenoid nutrition improved after consumption of green-yellow vegetables. Serum concentrations of retinol were sustained in the group fed green-yellow vegetables but decreased in the group fed light-colored vegetables ($P < 0.01$). The isotope-dilution tests confirmed that total-body vitamin A stores were sustained in the group fed green-yellow vegetables, but decreased 27 µmol (7700 µg retinol) per child, on average, in the group fed light-colored vegetables ($P < 0.06$).<br>Conclusion: Green-yellow vegetables can provide adequate vitamin A nutrition in the diet of kindergarten children and protect them from becoming vitamin A deficient during seasons when the provitamin A food source is limited.		
Vitamin and mineral requirements in human nutrition. 2004: World Health Organization	Sources and supply patterns of vitamin A	Most studies report a positive response when vegetable sources of provitamin A are given under controlled conditions to deficient subjects freed of confounding parasite loads and provided with sufficient dietary fat	Serum retinol concentrations in children are affected by food sources of b carotene, fat intake, and anthelmintic drug treatment [88]	The provision of vitamin A in food sources of beta-carotene is an alternative to the distribution of high-dose capsules. To examine factors that may influence the success of food-based programs, a study was carried out in Sumatra, Indonesia, of the effect of food sources of beta-carotene, extra dietary fat, and Ascaris lumbricoides infection on serum retinol concentrations in children. Meals and snacks with various amounts of beta-carotene and fat were fed at midday to children 3-6 y of age for 3 wk. Some groups of children were dewormed with the anthelmintic levamisole before the feeding period, whereas others remained infected. Results showed that the incorporation of beta-carotene sources (mainly in the form of red sweet potatoes) into the meal significantly increased serum retinol concentrations. The greatest rise in serum retinol occurred when meals contained added beta-carotene sources and added fat and the children were dewormed. Adding more fat to the meal and deworming the children caused a rise in serum retinol similar to that seen when feeding additional beta-carotene sources. Moreover, the effects of fat and deworming together were additive to the effects of additional beta-carotene sources. When the meal contained additional beta-carotene sources, added fat caused a further improvement in serum retinol concentrations but only if A. lumbricoides infection was low. These studies indicated that food-based interventions in vitamin A-deficient areas might be successful and that other interventions such as increasing dietary fat concentrations and anthelmintic treatment should be considered along with increasing consumption of beta-carotene-rich food.	2	RCT
Dietary Reference Intakes for Vitamin A, Vitamin K, Arsenic, Boron, Chromium, Copper, Iodine, Iron, Manganese, Molybdenum, Nickel, Silicon, Vanadium, and Zinc	Factors affecting the Vitamin A requirement	In some studies, increasing the level of fat in a low fat diet has been shown to improve … Vitamin A nutriture				

Vitamin and mineral requirements in human nutrition. 2004: World Health Organization	Indicators of vitamin A deficiency	subclinical deficiency may still be present at levels between 0.70 and 1.05mmol/l and occasionally above 1.05mmol/l	Assessment of marginal vitamin A deficiency in Brazilian children using the relative dose response procedure [89]	Vitamin A status was determined using fasting plasma levels and the relative dose response (RDR) procedure before and 30, 120, and 180 days after administration of an oral massive (200,000 IU) dose of vitamin A. The study was carried out in Recife, Brazil among 93 children of 7 yr or less from low income families who attended two day-care programs. The RDR procedure is conducted by obtaining a fasting blood (A0), feeding 450 retinol equivalents and obtaining a second blood specimen after 5 h (A5). The RDR-(A5-A0)/A5 X 100. A single massive oral dose of vitamin A was given after conducting the base-line RDR test. The RDR procedure was repeated at 30, 120, and 180 days. Weight and height measurements were obtained at each observation and the bloods were analyzed for Hb, total protein, and serum iron, as well as vitamin A. Serum albumin was determined in 120- and 180-day bloods. Serum iron levels were improved 30 days after supplementation with the massive dose of vitamin A. The RDR procedure was found practical to apply in mildly undernourished children under nonclinical condition. In this group of low income children presumed to be habitually ingesting minimally adequate diets, a serum vitamin A level of 20 micrograms/dl or less invariably was associated with an elevated RDR test. Blood levels between 20 to 40 micrograms/dl were not consistently predictive of the RDR response. All elevated RDR tests reverted to normal after supplementation with vitamin A, presumably indirectly indicating a presupplementation inadequate vitamin A status. Hence, the RDR was a more sensitive indicator of inadequate vitamin A status than was only a serum level of vitamin A when blood levels were above 20 micrograms/dl.	3d	Experimental - stores
Vitamin and mineral requirements in human nutrition. 2004: World Health Organization	Evidence used for making recommendations	During at least the first 6 months of life, exclusive breastfeeding can provide sufficient vitamin A to maintain health, permit normal growth, and maintain sufficient stores in the liver	Complementary feeding of young children in developing countries: a review of current scientific knowledge [90]	No abstract available		Published guidelines
Vitamin and mineral requirements in human nutrition. 2004: World Health Organization	Evidence used for making recommendations	In poor communities where children 1–6 years old are reported to have intakes	Reduced mortality among children in Southern India receiving a small	BACKGROUND. Clinical vitamin A deficiency affects millions of children worldwide, and subclinical deficiency is even more common. Supplemental vitamin A has been reported to reduce mortality among these children, but the results have been questioned. METHODS. We conducted a randomized, controlled, masked clinical trial for one year in southern India involving 15,419 preschool-age children who received either 8.7 µmol (8333 IU) of vitamin A and	2	RCT

		of about 100–200mg RE/day, signs of VAD do occur; in southern India these signs were relieved and risk of mortality was reduced when the equivalent of 350–400mg RE/day was given to children weekly	weekly dose of vitamin A [91]	46 µmol (20 mg) of vitamin E (the treated group) or vitamin E alone (the control group). Vitamin supplements were delivered weekly by community health volunteers who also recorded mortality and morbidity. Weekly contact was made with at least 88 percent of the children in both study groups. The base-line characteristics of the children were similar and documented a high prevalence of vitamin A deficiency and undernutrition. RESULTS. One hundred twenty-five deaths occurred, of which 117 were not accidental. The risk of death in the group treated with vitamin A was less than half that in the control group (relative risk, 0.46; 95 percent confidence interval, 0.30 to 0.71). The risk was most reduced among children under 3 years of age (6 to 11 months — relative risk, 0.28; 95 percent confidence interval, 0.09 to 0.85; 12 to 35 months — relative risk, 0.46; 95 percent confidence interval, 0.26 to 0.81) and among those who were chronically undernourished, as manifested by stunting (relative risk, 0.11; 95 percent confidence interval, 0.03 to 0.36). The symptom-specific risk of mortality was significantly associated with diarrhea, convulsions, and other infection-related symptoms. CONCLUSIONS.The regular provision of a supplement of vitamin A to children, at a level potentially obtainable from foods, in an area where vitamin A deficiency and under-nutrition are documented public health problems contributed substantially to children's survival; mortality was reduced on average by 54 percent		
Dietary Reference Intakes for Vitamin A, Vitamin K, Arsenic, Boron, Chromium, Copper, Iodine, Iron, Manganese, Molybdenum, Nickel, Silicon, Vanadium, and Zinc	Clinical Effects of Inadequate Intake	In developing countries, vitamin A supplementation has been shown to reduce the risk of mortality among young children				
Vitamin and mineral requirements in human nutrition. 2004: World Health Organization	Evidence used for making recommendations	The safe intake for children was compared with the distribution of intakes and comparable serum vitamin A levels reported for children 0–6 years of age from the United States and with distributions of serum levels of vitamin A of children	Age-specific reference intervals for plasma vitamins A, E and beta-carotene and for serum zinc, retinol-binding protein and prealbumin for Sydney children aged 9-62 months [92]	Paediatric reference intervals for blood concentrations of certain nutrients are often based on either adult data or are derived from small samples of young children. Biochemical data were obtained from 467 randomly selected, healthy preschool children aged 9-62 months in Sydney, Australia. Data were obtained for plasma vitamins A, E and beta-carotene and for serum zinc, retinol-binding protein and prealbumin. Reference intervals based on the 2.5 and 97.5 centiles for age groups 9-23, 24-35, 36-47, 48-62 months and for the total group (9-62 months) were calculated. The 2.5-97.5 centiles for the whole group were: vitamin A, 0.7-1.8 mumol/l (20.05-51.56 micrograms/dl); vitamin E, 8-30 mumol/l (0.34-1.29 mg/dl); beta-carotene, 0.1-1.1 mumol/l (5.4-59.0 micrograms/dl); zinc, 9-19 mumol/l (58.8-124.2 micrograms/dl); retinol-binding protein, 14-36 mg/l; prealbumin, 104-264 mg/l. The reference intervals reported are consistent with the findings of a number of smaller studies and are likely to be an accurate reflection of the true intervals for healthy preschool children in western developed countries.	5	Observational

		aged 9–62 months in Australia, where evidence of VAD is rare.				
Dietary Reference Intakes for Vitamin A, Vitamin K, Arsenic, Boron, Chromium, Copper, Iodine, Iron, Manganese, Molybdenum, Nickel, Silicon, Vanadium, and Zinc	Absorption and bioconversion	Relative Absorption of Supplemental and Dietary β-Carotene	Lack of improvement in vitamin A status with increased consumption of dark-green leafy vegetables [93]	There is little evidence to support the general assumption that dietary carotenoids can improve vitamin A status. We investigated in Bogor District, West Java, Indonesia, the effect of an additional daily portion of dark-green leafy vegetables on vitamin A and iron status in women with low haemoglobin concentrations (< 130 g/L) who were breastfeeding a child of 3-17 months. Every day for 12 weeks one group (n = 57) received stir-fried vegetables, a second (n = 62) received a wafer enriched with beta-carotene, iron, vitamin C, and folic acid, and a third (n = 56) received a non-enriched wafer to control for additional energy intake. The vegetable supplement and the enriched wafer contained 3.5 mg beta-carotene, 5.2 mg and 4.8 mg iron, and 7.8 g and 4.4 g fat, respectively. Assignment to vegetable or wafer groups was by village. Wafers were distributed double-masked. In the enriched-wafer group there were increases in serum retinol (mean increase 0.32 [95% CI 0.23-0.40] mumol/L), breastmilk retinol (0.59 [0.35-0.84] mumol/L), and serum beta-carotene (0.73 [0.59-0.88] mumol/L). These changes differed significantly from those in the other two groups, in which the only significant changes were small increases in breastmilk retinol in the control-wafer group (0.16 [0.02-0.30] mumol/L) and in serum beta-carotene in the vegetable group (0.03 [0-0.06] mumol/L). Changes in iron status were similar in all three groups. An additional daily portion of dark-green leafy vegetables did not improve vitamin A status, whereas a similar amount of beta-carotene from a simpler matrix produced a strong improvement. These results suggest that the approach to combating vitamin A deficiency by increases in the consumption of provitamin A carotenoids from vegetables should be re-examined.	3a	Experimental – absorption
Dietary Reference Intakes for Vitamin A, Vitamin K, Arsenic, Boron, Chromium, Copper, Iodine, Iron, Manganese, Molybdenum, Nickel, Silicon, Vanadium, and Zinc	Absorption and bioconversion	Relative Absorption of Supplemental and Dietary β-Carotene	Orange fruit is more effective than dark-green, leafy vegetables in increasing serum concentrations of retinol and beta-carotene in schoolchildren in Indonesia [94]	The objectives of this study were to quantify the effectiveness of dietary retinol sources, orange fruit, and dark-green, leafy vegetables in improving vitamin A status, and to test whether orange fruit is a better source of vitamin A and carotenoids than are leafy vegetables. Anemic schoolchildren aged 7-11 y (n = 238) in West Java, Indonesia, were randomly allocated to 1 of 4 groups to consume 2 complete meals/d, 6 d/wk, for 9 wk: 1) 556 retinol equivalents (RE)/d from retinol-rich food (n = 48); 2) 509 RE/d from fruit (n = 49); 3) 684 RE/d from dark-green, leafy vegetables and carrots (n = 45); and 4) 44 RE/d from low-retinol, low-carotene food (n = 46). Mean changes in serum retinol concentrations of the retinol-rich, fruit, vegetable, and low-retinol, low-carotene groups were 0.23 (95% CI: 0.18, 0.28), 0.12 (0.06, 0.18), 0.07 (0.03,0.11), and 0.00 (-0.06, 0.05) micromol/L, respectively. Mean changes in serum beta-	3a	Experimental – absorption

				carotene concentrations in the vegetable and fruit groups were 0.14 (0.12, 0.17) and 0.52 (0.43, 0.60) micromol/L, respectively. Until now, it has been assumed that 6 microg dietary beta-carotene is equivalent to 1 RE. On the basis of this study, however, the equivalent of 1 RE would be 12 microg beta-carotene (95% CI: 6 microg, 29 microg) for fruit and 26 microg beta-carotene (95% CI: 13 microg, 76 microg) for leafy vegetables and carrots. Thus, the apparent mean vitamin A activity of carotenoids in fruit and in leafy vegetables and carrots was 50% (95% CI: 21%, 100%) and 23% (95% CI: 8%, 46%) of that assumed, respectively. This has important implications for choosing strategies for controlling vitamin A deficiency. Research should be directed toward ways of improving bioavailability and bioconversion of dietary carotenoids, focusing on factors such as intestinal parasites, absorption inhibitors, and food matrixes.		
Dietary Reference Intakes for Vitamin A, Vitamin K, Arsenic, Boron, Chromium, Copper, Iodine, Iron, Manganese, Molybdenum, Nickel, Silicon, Vanadium, and Zinc	Clinical Effects of Inadequate Intake	It is estimated that 3 to 10 million children, mostly in developing countries, become xerophthalmic, and 250,000 to 500,000 go blind annually	Vitamin A Deficiency: Health, Survival, and Vision [95]	No abstract available.	6	Non-systematic review
Dietary Reference Intakes for Vitamin A, Vitamin K, Arsenic, Boron, Chromium, Copper, Iodine, Iron, Manganese, Molybdenum, Nickel, Silicon, Vanadium, and Zinc	Clinical Effects of Inadequate Intake	Night blindness is the first ocular symptom to be observed with vitamin A deficiency, and it responds rapidly to treatment with vitamin A	*Nutritional Blindness. Xerophthalmia and Keratomalacia* [96]	No abstract available.	6	Non-systematic review
Dietary Reference Intakes for Vitamin A, Vitamin K, Arsenic, Boron, Chromium, Copper, Iodine, Iron, Manganese, Molybdenum,	Clinical Effects of Inadequate Intake	A higher risk of respiratory infection and diarrhea has been reported among children with mild to moderate	Increased risk of respiratory disease and diarrhea in children with pre-existing mild vitamin A deficiency [97]	Preschool-age rural Indonesian children were reexamined every 3 months for 18 months. An average of 3135 children were free of respiratory disease and or diarrhea at the examination initiating each of the six, 3-month follow-up intervals. Children with mild xerophthalmia (night blindness and/or Bitot's spots) at the start and end of an interval developed respiratory disease and diarrhea at twice ($p < 0.001$) and three times ($p < 0.001$) the rate, respectively, of children with normal eyes during the same interval, independent of age and anthropometric status (weight for length). The risk of respiratory disease and	5	Observational

Nickel, Silicon, Vanadium, and Zinc		vitamin A deficiency		diarrhea were more closely associated with vitamin A status than with general nutritional status. These results may explain much of the excess mortality recently reported for mildly vitamin A-deficient children.		
Dietary Reference Intakes for Vitamin A, Vitamin K, Arsenic, Boron, Chromium, Copper, Iodine, Iron, Manganese, Molybdenum, Nickel, Silicon, Vanadium, and Zinc	Clinical Effects of Inadequate Intake	Mortality rates were about four times greater among children with mild xerophthalmia than those without it	Increased mortality in children with mild vitamin A deficiency [98]	An average of 3481 preschool-age rural Indonesian children were re-examined every 3 months for 18 months. The mortality rate among children with mild xerophthalmia (night blindness and/or Bitot's spots) was on average 4 times the rate, and in some age groups 8 to 12 times the rate, among children without xerophthalmia. Mortality increased, almost linearly, with the severity of mild xerophthalmia (night blindness, Bitot's spots, and the two combined). These relations persisted after stratification for respiratory disease, wasting, gastroenteritis, pedal oedema, and childhood exanthems. Mild vitamin A deficiency was directly associated with at least 16% of all deaths in children aged from 1 to 6 years. These results suggest that mild xerophthalmia justifies vigorous community-wide intervention, as much to reduce childhood mortality as to prevent blindness, and that night blindness and Bitot's spots are as important as anthropometric indices in screening children to determine which of them need medical and nutritional attention.	5	Observational
Dietary Reference Intakes for Vitamin A, Vitamin K, Arsenic, Boron, Chromium, Copper, Iodine, Iron, Manganese, Molybdenum, Nickel, Silicon, Vanadium, and Zinc	Clinical Effects of Inadequate Intake	In children hospitalized with measles, case fatality (Barclay et al., 1987; Hussey and Klein, 1990) and the severity of complications on admission were reduced when they received high doses (60 to 120 mg) of vitamin A (Coutsoudis et al., 1991; Hussey and Klein, 1990).	Vitamin A supplements and mortality related to measles: A randomised clinical trial [99]	One hundred and eighty children admitted with measles were randomly allocated to receive routine treatment alone or with additional large doses of vitamin A (200,000 IU orally immediately and again the next day). Baseline characteristics of the two groups were virtually identical for age, severity of measles, and vitamin A and general nutritional states. In 91% of the children serum vitamin A concentrations were less than 0.56 mumol/l. Of the 88 subjects given vitamin A supplements, six (7%) died; of the 92 controls, 12 (13%) died (p = 0.13). This difference in mortality was most obvious for children aged under 2 years (one death out of 46 children receiving supplements versus seven deaths out of 42 controls; p less than 0.05) and for cases complicated by croup or laryngotracheobronchitis. Mortality was several times higher in marasmic than in better nourished children, regardless of study allocation (p less than 0.01).	2	RCT
Dietary Reference Intakes for Vitamin A, Vitamin K, Arsenic, Boron, Chromium, Copper, Iodine, Iron,	Clinical Effects of Inadequate Intake	In some studies, vitamin A supplementation (30 to 60 mg) has been shown to	Randomized placebo-controlled clinical trial of the effect of a single high dose or daily low	The effect of high-dose vitamin A supplementation on recovery from morbidity and on recovery from nosocomial morbidity of hospitalized children has been poorly studied and results are conflicting. The effect of daily, low doses has never been assessed. We investigated the effect of a single high dose and daily, low doses of vitamin A on diarrhea, acute lower respiratory tract infections (ALRIs), and all-cause fevers in 900 hospitalized preschool-age children in the	2	RCT

Manganese, Molybdenum, Nickel, Silicon, Vanadium, and Zinc		reduce the severity of diarrhea	doses of vitamin A on the morbidity of hospitalized, malnourished children [100]	Democratic Republic of Congo in a randomized, double-blind, placebo-controlled clinical trial. The high-dose treatment group received 200,000 IU vitamin A (100,000 IU if aged <12 mo) orally on the day of admission, the low-dose treatment group received 5000 IU vitamin A/d until discharge. Data on all-cause morbidity were collected daily. Mortality rates were not significantly different among the 3 groups. High-dose vitamin A supplementation had no significant effect on the duration of moderate or severe diarrhea nor on the duration and incidence of ALRIs and all-cause fevers. Children in the high-dose group with no edema had an increased risk of severe nosocomial diarrhea (relative risk: 2.42; 95% CI: 1.15, 5.11). Low-dose vitamin A supplementation significantly reduced the incidence of severe diarrhea in severely malnourished children (relative risk: 0.21; 95% CI: 0.07, 0.62) but showed no significant effect on the duration of moderate or severe diarrhea or on the duration and incidence of ALRIs and all-cause fevers. Supplementation with high doses of vitamin A did not reduce morbidity in this population of malnourished and subclinically vitamin A-deficient children; daily, low doses appeared more beneficial for severely malnourished children.		
Dietary Reference Intakes for Vitamin A, Vitamin K, Arsenic, Boron, Chromium, Copper, Iodine, Iron, Manganese, Molybdenum, Nickel, Silicon, Vanadium, and Zinc	Clinical Effects of Inadequate Intake	In some studies, vitamin A supplementation (30 to 60 mg) has been shown to reduce the severity of diarrhea and Plasmodium falciparum malaria	Effect of vitamin A supplementation on morbidity due to Plasmodium falciparum in young children in Papua New Guinea: a randomised trial [101]	**Background:** Many individuals at risk of malaria also have micronutrient deficiencies that may hamper protective immunity. Vitamin A is central to normal immune function, and supplementation has been shown to lower the morbidity of some infectious diseases. We investigated the effect of vitamin A supplementation on malaria morbidity. **Methods:** This randomised double-blind placebo-controlled trial of vitamin A supplementation took place in a P. falciparum endemic area of Papua New Guinea. Of 520 potentially eligible children aged 6-60 months, 480 were randomly assigned high-dose vitamin A (n=239) or placebo (n=241), every 3 months for 13 months. Malaria morbidity was assessed through weekly community-based case detection and surveillance of patients who self-reported to the health centre. Cross-sectional surveys were also done at the beginning, middle, and end of the study to assess malariometric indicators. Analyses were by intention to treat. **Findings:** The number of P. falciparum febrile episodes (temperature > or = 37.5 degrees C with a parasite count of at least 8000/microL) was 30% lower in the vitamin A group than in the placebo group (178 vs 249 episodes; relative risk 0.70 [95% CI 0.57-0.87], p=0.0013). At the end of the study P. falciparum geometric mean density was lower in the vitamin A than the placebo group (1300 [907-1863] vs 2039 [1408-2951]) as was the proportion with spleen enlargement (125/196 [64%] vs 148/207 [71%]); neither difference was significant (p=0.093 and p=0.075). Children aged 12-36 months benefited most, having 35% fewer febrile episodes (89 vs 141; relative risk 0.65 [14-50], p=0.0023), 26% fewer enlarged spleens (46/79 [58%] vs 67/90 [74%], p=0.0045), and a 68% lower parasite density (1160	2	RCT

				[95% CI 665-2022] vs 3569 [2080-6124], p=0.0054). Vitamin A had no consistent effect on cross-sectional indices of proportion infected or with anaemia. **Interpretation:** Vitamin A supplementation may be an effective low-cost strategy to lower morbidity due to P. falciparum in young children. The findings suggest that clinical episodes, spleen enlargement, and parasite density are influenced by different immunological mechanisms from infection and anaemia.		
Dietary Reference Intakes for Vitamin A, Vitamin K, Arsenic, Boron, Chromium, Copper, Iodine, Iron, Manganese, Molybdenum, Nickel, Silicon, Vanadium, and Zinc	Clinical Effects of Inadequate Intake	vitamin A supplementation has had little effect on the risk or severity of respiratory infections, except when associated with measles	Impact of neonatal vitamin A supplementation on infant morbidity and mortality [102]	**Objective:** To determine whether vitamin A supplementation at birth could reduce infant morbidity and mortality. **Study design:** We conducted a placebo-controlled trial among 2067 Indonesian neonates who received either 52 micromol (50,000 IU) orally administered vitamin A or placebo on the first day of life. Infants were followed up at 1 year to determine the impact of this intervention on infant mortality. A subgroup (n = 470) was also examined at 4 and 6 months of age to examine the impact on morbidity. **Results:** Vital status was confirmed in 89% of infants in both groups at 1 year. There were 19 deaths in the control group and 7 in the vitamin A group (relative risk = 0.36; 95% confidence interval = 0.16, 0.87). The impact was stronger among boys, infants of normal compared with low birth weight, and those of greater ponderal index. Among infants examined at 4 months of age, the 1-week period prevalence of common morbidities was similar for vitamin A and control infants. However, during this same 4-month period, 73% and 51% more control infants were brought for medical treatment for cough (p = 0.008) and fever (p = 0.063), respectively. **Conclusions:** Neonatal vitamin A supplementation may reduce the infant mortality rate and the prevalence of severe respiratory infection among young infants.	2	RCT
Dietary Reference Intakes for Vitamin A, Vitamin K, Arsenic, Boron, Chromium, Copper, Iodine, Iron, Manganese, Molybdenum, Nickel, Silicon, Vanadium, and Zinc	Clinical Effects of Inadequate Intake	In developing countries, vitamin A supplementation has been shown to reduce the risk of mortality among young children	Vitamin A-fortified monosodium glutamate and health, growth, and survival of children: A controlled field trial [103]	In a controlled trial, fortification of commercially marketed monosodium glutamate (MSG) with vitamin A improved serum vitamin A levels of young children and the vitamin A content of breast milk of lactating women. These improvements in vitamin A indices were accompanied by dramatic changes in health and anthropometric status. During the course of the study, the prevalence of Bitot's spots among children in program villages fell progressively from 1.2% at base line to 0.2% 11 mo after introduction of the fortified product (p less than 0.001); xerophthalmia rates in control villages remained essentially unchanged. Linear growth was greater among program than among control children at every age. Hemoglobin levels among program children rose by approximately 10 g, from 113 +/- 16 g/L at base line to 123 +/- 16 by 5 mo (p less than 0.001); they remained essentially unchanged among children of control villages. Preschool children in control villages died at 1.8 times the rate of children in program villages.	2	RCT

Dietary Reference Intakes for Vitamin A, Vitamin K, Arsenic, Boron, Chromium, Copper, Iodine, Iron, Manganese, Molybdenum, Nickel, Silicon, Vanadium, and Zinc	Clinical Effects of Inadequate Intake	In developing countries, vitamin A supplementation has been shown to reduce the risk of mortality among young children	Impact of vitamin A supplementation on childhood mortality: A randomized controlled community trial [104]	450 villages in northern Sumatra were randomly assigned to either participate in a vitamin A supplementation scheme (n = 229) or serve for 1 year as a control (n = 221). 25 939 preschool children were examined at baseline and again 11 to 13 months later. Capsules containing 200 000 IU vitamin A were distributed to preschool children aged over 1 year by local volunteers 1 to 3 months after baseline enumeration and again 6 months later. Among children aged 12-71 months at baseline, mortality in control villages (75/10 231, 7.3 per 1000) was 49% greater than in those where supplements were given (53/10 919, 4.9 per 1000) (p less than 0.05). The impact of vitamin A supplementation seemed to be greater in boys than in girls. These results support earlier observations linking mild vitamin A deficiency to increased mortality and suggest that supplements given to vitamin A deficient populations may decrease mortality by as much as 34%.	2	RCT
Dietary Reference Intakes for Vitamin A, Vitamin K, Arsenic, Boron, Chromium, Copper, Iodine, Iron, Manganese, Molybdenum, Nickel, Silicon, Vanadium, and Zinc	Clinical Effects of Inadequate Intake	In developing countries, vitamin A supplementation has been shown to reduce the risk of mortality among young children	Efficacy of vitamin A in reducing preschool child mortality in Nepal [105]	Community trials of the efficacy of vitamin A supplementation in reducing preschool childhood mortality have produced conflicting results. To resolve the question, a randomised, double-masked, placebo-controlled community trial of 28 630 children aged 6-72 months was carried out in rural Nepal, an area representative of the Gangetic flood plain of South Asia. Randomisation was carried out by administrative ward; the vitamin-A-supplemented children received 60 000 retinol equivalents every 4 months and placebo-treated children received identical capsules containing 300 retinol equivalents. After 12 months, the relative risk of death in the vitamin-A-supplemented compared with the control group was 0·70 (95% confidence interval 0·56-0·88), equivalent to a 30% reduction in mortality. The trial, which had been planned to last 2 years, was discontinued. The reduction in mortality was present in both sexes (relative risk for boys 0·77; for girls 0·65), at all ages (range of relative risks 0·83-0·50), and throughout the year (0·76-0·67). The reduction in mortality risk was not affected by acute nutritional status, as measured by arm circumference. Thus, periodic vitamin A delivery in the community can greatly reduce child mortality in developing countries.	2	RCT
Dietary Reference Intakes for Vitamin A, Vitamin K, Arsenic, Boron, Chromium, Copper, Iodine, Iron, Manganese, Molybdenum, Nickel, Silicon, Vanadium, and Zinc	Clinical Effects of Inadequate Intake	WHO recommends broad-based prophylaxis in vitamin A-deficient populations. It also recommends treating children who suffer from xerophthalmia, measles, prolonged	*Vitamin A Supplements: A Guide to Their Use in the Treatment of Vitamin A Deficiency and Xerophthalmia* [106]	**No abstract available**		Published guidelines

		diarrhea, wasting malnutrition, and other acute infections with vitamin A				
Dietary Reference Intakes for Vitamin A, Vitamin K, Arsenic, Boron, Chromium, Copper, Iodine, Iron, Manganese, Molybdenum, Nickel, Silicon, Vanadium, and Zinc	Clinical Effects of Inadequate Intake	the American Academy of Pediatrics recommends vitamin A supplementation for children in the United States who are hospitalized with measles.	Vitamin A treatment of measles [107]	In spite of the availability of effective vaccines, measles continues to be a public health problem throughout the world. In 1990, the Centers for Disease Control received more than 27 672 reports of measles in the United States. Complications were reported in one third of infected children younger than 5 years of age.[1] Of the 89 measles-related deaths in 1990, 55% occurred in children younger than 5 years old. Several recent investigations have indicated that vitamin A treatment of children with measles in developing countries has been associated with reductions in morbidity and mortality. The World Health Organization (WHO) and the United Nations International Children's Emergency Fund (UNICEF) issued a joint statement recommending that vitamin A be administered to all children diagnosed with measles in communities where vitamin A deficiency (serum vitamin A <10 µg/dL) is a recognized problem and where mortality related to measles is ≥1%. The recommended regimen is 100 000 IU by mouth at the time of diagnosis for infants younger than 12 months of age, and 200 000 IU for older children. In the presence of ophthalmologic signs of vitamin A deficiency, such as night blindness, Bitot's spots (grayish white deposits on the bulbar conjunctiva adjacent to the cornea) or xerophthalmia, the WHO recommends the dose be repeated in 24 hours and again 4 weeks later.[2] Vitamin A is available in low-cost liquid formulations and is supplemented in infant formulas (2000 µ/L).		Published guidelines
Dietary Reference Intakes for Vitamin A, Vitamin K, Arsenic, Boron, Chromium, Copper, Iodine, Iron, Manganese, Molybdenum, Nickel, Silicon, Vanadium, and Zinc	Selection of indicators for estimating the requirement for Vitamin A	Epidemiological evidence suggests that host resistance to infection is impaired at lesser stages of vitamin A deficiency, prior to clinical onset of night blindness	Impact of vitamin A supplementation on childhood morbidity in northern Ghana [108]	No abstract available	2	RCT
Dietary Reference Intakes for Vitamin A, Vitamin K, Arsenic, Boron, Chromium, Copper,	Selection of indicators for estimating the requirement for Vitamin A	Epidemiological evidence suggests that host resistance to infection is impaired at lesser	Mild vitamin A deficiency and risk of respiratory tract diseases and diarrhea in	A cross-sectional study, a follow-up study, and an intervention trial were carried out to investigate the association between mild vitamin A deficiency and the occurrence of diarrhea and respiratory diseases. Cross-sectional analysis was performed among 1,772 children, aged 1-8 years, in the Sakon Nakhon province of northeastern Thailand. Children with a history of diarrhea or respiratory	5	Observational

Iodine, Iron, Manganese, Molybdenum, Nickel, Silicon, Vanadium, and Zinc		stages of vitamin A deficiency, prior to clinical onset of night blindness	preschool and school children in northeastern Thailand [109]	disease had lower levels of serum retinol and retinol-binding protein. Adjusted for age, sex, nutritional status, and level of urbanization, logistic regression using data for 877 children showed a negative association between serum retinol and both diarrhea and respiratory diseases. A follow-up three months later (n = 146 children) showed that children with deficient serum retinol (less than 0.35 mumol/liter) had a fourfold greater risk of respiratory disease (p less than 0.01). No relation was found for diarrhea. An intervention trial (n = 166 children aged 1-5 years) showed that, during 2 months of follow-up after administration of oral vitamin A (200,000 IU), the control group (aged 3-5 years) had a higher incidence of respiratory disease (2.9 times) as well as diarrhea (3.1 times). Between 2 and 4 months, a significantly (p less than 0.025) higher incidence of respiratory diseases (2.5 times) could be observed in children aged 1-2 years. This study supports earlier reports on a greater risk of respiratory diseases and of diarrhea in mild vitamin A deficiency. Supplementation reduced the incidence of both diarrhea and respiratory disease for a period of at least 2 months.		
Dietary Reference Intakes for Vitamin A, Vitamin K, Arsenic, Boron, Chromium, Copper, Iodine, Iron, Manganese, Molybdenum, Nickel, Silicon, Vanadium, and Zinc	Selection of indicators for estimating the requirement for Vitamin A	Epidemiological evidence suggests that host resistance to infection is impaired at lesser stages of vitamin A deficiency, prior to clinical onset of night blindness	Vitamin A deficiency in Micronesia: A statewide survey in Chuuk [110]	Vitamin A status was assessed by clinical signs and conjunctival impression cytology (CIC-A) in 455 children 36–83 months of age in 16 at randomly selected villages in Chuuk State. Fourteen percent of the children had a history of night blindness and 6% had Bitot's spots on examination. Nearly half of the children (46%) had abnormal CIC-A status reflecting subclinical vitamin A deficiency. Children abnormal by CIC-A had lower hemoglobin levels (−0.4 µg/dl), were more stunted in their linear growth, and were twice as likely to have had a recent respiratory infection than children with normal CIC-A. Vitamin A deficiency appears to be a serious public health problem in Micronesia.	5	Observational
Dietary Reference Intakes for Vitamin A, Vitamin K, Arsenic, Boron, Chromium, Copper, Iodine, Iron, Manganese, Molybdenum, Nickel, Silicon, Vanadium, and Zinc	Selection of indicators for estimating the requirement for Vitamin A	Epidemiological evidence suggests that host resistance to infection is impaired at lesser stages of vitamin A deficiency, prior to clinical onset of night blindness	Association of diarrhea and low serum retinol in Peruvian children [111]	To examine the relationship between acute diarrhea and vitamin A status, a study was conducted in 137 children (72 with diarrhea and 65 illness-free control subjects) in the city of Lima, Peru. Serum retinol was measured spectrophotometrically in samples collected in 1987 and kept frozen until they were analyzed simultaneously in 1989. Serum retinol was significantly lower in the children with diarrhea (mean +/- SD: 0.51 +/- 0.48 mumol/L) than in those without diarrhea (1.00 +/- 0.32 mumol/L; 1 mumol/L retinol = 28.6 micrograms/dL). The multivariate estimate of the effect of diarrhea (-0.464 mumol/L) in a model that incorporated age, sex, and acute malnutrition (ie, weight-for-height) as confounding variables was essentially the same as the unadjusted difference (-0.492 mumol/L). Thus, this model showed that the retinol concentration in the serum depends greatly on the presence of diarrhea. These findings suggest that diarrhea, as has been shown for other infections, may lead to lower circulating retinol concentrations and perhaps to its depletion.	5	Observational

Dietary Reference Intakes for Vitamin A, Vitamin K, Arsenic, Boron, Chromium, Copper, Iodine, Iron, Manganese, Molybdenum, Nickel, Silicon, Vanadium, and Zinc	Selection of indicators for estimating the requirement for Vitamin A	An early report of pupillary nonresponse to candlelight among night blind Confederate soldiers in the Civil War (Hicks, 1867) led to the development and validation of instrumentation for this test as a reliable, functional measure of vitamin A deficiency in Indonesian and Indian children.	Pupillary and visual thresholds in young children as an index of population vitamin A status [112]	A prototype scotopic sensitivity machine was used to evaluate pupillary and visual thresholds for 295 Indonesian children aged 1-5 y, most of whom were initially vitamin A-deficient. Subjects were tested 6 and 9 mo after receiving a high dose of vitamin A. A group of 136 older children was tested at 6 mo after dosing; all subjects underwent testing at 9 mo. After testing at 9 mo, children randomly received either a second high dose of vitamin A or placebo and were tested a final time 2 wk later. Children with abnormal pupillary thresholds had significantly higher relative dose responses (RDRs) (P < 0.01) and significantly lower serum retinol values (P = 0.05) than did normal children. The mean pupillary threshold rose (eg, retinal sensitivity fell) as vitamin A status deteriorated between 6 and 9 mo after initial dosing, and was significantly different from a group of normal American children tested previously (P < 0.001). After placebo-controlled dosing, the decline in pupillary and visual thresholds (rise in retinal sensitivity) was significant for children receiving vitamin A but not for children receiving placebo.	2	RCT
Dietary Reference Intakes for Vitamin A, Vitamin K, Arsenic, Boron, Chromium, Copper, Iodine, Iron, Manganese, Molybdenum, Nickel, Silicon, Vanadium, and Zinc	Selection of indicators for estimating the requirement for Vitamin A	An early report of pupillary nonresponse to candlelight among night blind Confederate soldiers in the Civil War (Hicks, 1867) led to the development and validation of instrumentation for this test as a reliable, functional measure of vitamin A deficiency in Indonesian and Indian children.	Pupillary threshold as an index of population vitamin A status among children in India [113]	Two hundred seven vitamin A-deficient southern Indian children aged 1-7 y (mean age: 56.9 mo) underwent testing of dark-adapted visual and pupillary thresholds in their village setting according to a previously reported protocol. One hundred thirty (62.8%) of the children also underwent serum retinol testing, and 178 (86.0%) participated in a randomized, placebo-controlled vitamin A dosing trial with pre- and postdose testing of dark-adaptation threshold. Most subjects (184 of 207, 88.9%) were able to complete pupillary testing, an objective sign requiring minimal cooperation, including a high proportion of the youngest children (72.2% of subjects aged 2 y). The proportion of children completing visual threshold testing, which requires greater understanding and cooperation, was significantly smaller than that able to complete pupillary testing (131 of 207, 63.3%; P < 0.0001, chi square). At baseline (predosing), the mean serum retinol concentration declined in linear fashion with a higher pupillary threshold (0.73 mumol/L with a score < or = 4; 0.47 mumol/L with a score > or = 8; P < 0.01). The mean pupillary threshold for these highly vitamin A-deficient Indian children (-0.622 log cd/m2) was significantly higher than that for 136 more moderately deficient Indonesian children (-0.985 log cd/m2; P < 0.001, two-sample t test) and 56 normal American children (-1.335 log cd/m2; P < 0.0001, two-sample t test). The improvement in pupillary dark-adaptation testing was not significant for children receiving vitamin A or placebo, though there was a nonsignificant trend toward greater improvement in children	2	RCT

				receiving vitamin A (P = 0.2, two-sample t test). Pupillary threshold testing represents a new, noninvasive, practical, and seemingly valid approach to assessing the vitamin A status of a moderately to severely deficient preschool population.		
Dietary Reference Intakes for Vitamin A, Vitamin K, Arsenic, Boron, Chromium, Copper, Iodine, Iron, Manganese, Molybdenum, Nickel, Silicon, Vanadium, and Zinc	Selection of indicators for estimating the requirement for Vitamin A	When dietary vitamin A is provided to vitamin A-deficient children, plasma retinol concentration increases rapidly, even before liver stores are restored	Biological availability of β-carotene from fresh and dried green leafy vegetables on preschool children [114]	The biological availability of Beta carotene from fresh and dried forms of fenugreek and drumstick leaves to rural preschool children in India was studied over two and a half months. Supplementation resulted in an increase in the concentration of serum vitamin A levels irrespective of the source of supplement. The Beta carotene absorption obtained for children receiving fresh fenugreek leaf preparations was greater than in groups receiving dried preparations. Absorption from fresh fenugreek supplementation was greater than that from fresh drumstick leaves.	2	RCT
Dietary Reference Intakes for Vitamin A, Vitamin K, Arsenic, Boron, Chromium, Copper, Iodine, Iron, Manganese, Molybdenum, Nickel, Silicon, Vanadium, and Zinc	Selection of indicators for estimating the requirement for Vitamin A	Plasma retinol concentration may also be low during infection as a result of transient decreases in the concentrations of the negative acute phase proteins, RBP, and transthyretin, even when liver retinol is adequate	Vitamin A supplementation, morbidity, and serum acute-phase proteins in young Ghanaian children [115]	The association of vitamin A supplementation with concentrations of positive acute-phase proteins in the serum was investigated in the Child Health Study of the Ghana Vitamin A Supplementation Trials, a randomized, controlled trial of the effect of vitamin A on morbidity in children aged < 5 y. Mean serum concentrations of alpha 1-acid glycoprotein, serum amyloid A, and C-reactive protein did not differ overall between the vitamin A-supplemented and placebo-treated groups. Treatment groups were then subdivided according to what symptoms children had experienced in the week before blood sampling. Acute-phase-protein responses to fever and cough were not affected by vitamin A supplementation. There was a tendency for vitamin A-supplemented children, but not placebo children, to have elevated acute-phase proteins in association with reported vomiting or severe diarrhea. The failure of unsupplemented children to mount an acute-phase response may have contributed to their increased morbidity from gastrointestinal symptoms.	2	RCT
Dietary Reference Intakes for Vitamin A, Vitamin K, Arsenic, Boron, Chromium, Copper, Iodine, Iron, Manganese, Molybdenum, Nickel, Silicon, Vanadium, and Zinc	Selection of indicators for estimating the requirement for Vitamin A	In the United States, serum retinol concentration is rarely low (< 0.7 µmol/ L) in more than 5 percent of preschool children, although 20 to 60 percent may exhibit concentrations	Ethnic and racial differences in serum vitamin A levels of children aged 4–11 years [116]	Interpretation of differences in serum vitamin A levels observed between Hispanic and non-Hispanic children may be complicated by confounding environmental factors. Data from the Mexican-American portion of the Hispanic Health and Nutrition Examination Survey and the second National Health and Nutrition Examination Survey were used to explore these differences in 4-11-y-old Mexican Americans and non-Hispanic blacks and whites before and after accounting for vitamin-mineral supplement use and poverty status. Initial differences in mean serum vitamin A levels and prevalences less than 20 micrograms/dL (0.70 mumol/L) or less than 25 micrograms/dL (0.87 mumol/L) among the three ethnic or racial groups were reduced or eliminated after accounting for the two descriptive variables. These results support the hypothesis that differences in serum vitamin A levels between Mexican-	5	Observational

		between 0.70 and 1.05 µmol/L		American and non-Hispanic children in the United States are due more to environmental factors than to ethnicity.		
Dietary Reference Intakes for Vitamin A, Vitamin K, Arsenic, Boron, Chromium, Copper, Iodine, Iron, Manganese, Molybdenum, Nickel, Silicon, Vanadium, and Zinc	Selection of indicators for estimating the requirement for Vitamin A	Before the clinical onset of xerophthalmia, mild vitamin A deficiency leads to early keratinizing metaplasia and losses of mucinsecreting goblet cells on the bulbar surface of the conjunctiva of the eye. These functional changes on the ocular surface can be detected by microscopic examination of PAS-hematoxylin stained epithelial cells obtained by briefly applying a cellulose acetate filter paper strip.	Impression cytology for detection of vitamin A deficiency [117]	Vitamin A (retinol) deficiency causes blindness, increased morbidity, and mortality among preschool children in many developing nations. Previous studies suggest that impression cytology may represent the first simple, reliable test to detect mild xerophthalmia in young children. We used impression cytology toevaluate and follow up 75 Indonesian preschool children with mild xerophthalmia and an equal number of age-matched, clinically normal neighborhood controls. Results of impression cytology, which were closely correlated with baseline serum vitamin A levels, documented histologic improvement following treatment with vitamin A. Furthermore, results of impression cytology, where abnormal, improved to normal following vitamin A treatment in a significant percentage (23%) of otherwise clinically normal children. Impression cytology appears to detect clinical and physiologically significant preclinical vitamin A deficiency.	5	Observ ational
Dietary Reference Intakes for Vitamin A, Vitamin K, Arsenic, Boron, Chromium, Copper, Iodine, Iron, Manganese, Molybdenum, Nickel, Silicon, Vanadium, and Zinc	Selection of indicators for estimating the requirement for Vitamin A	Before the clinical onset of xerophthalmia, mild vitamin A deficiency leads to early keratinizing metaplasia and losses of mucinsecreting goblet cells on the bulbar surface of the conjunctiva of the eye. These	Detection of early xerophthalmia by impression cytology [118]	Vitamin A deficiency causes blindness, increased systemic morbidity, and increased mortality among preschool children in many developing nations. Presently, there is no simple, reliable test to detect early, physiologically significant vitamin A deficiency. We used conjunctival impression cytology results to evaluate children with early xerophthalmia before treatment and again three to eight weeks later. Subsequently, we modified our technique. We then compared children with early xerophthalmia to normal children. Conjunctival impressions from children with xerophthalmia all showed complete loss of goblet cells and the appearance of enlarged, partially keratinized epithelial cells. Conjunctival impressions from treated and normal children showed normal goblet cells and sheets of small epithelial cells. These results suggest impression cytology may represent the first simple, objective, diagnostic test for the detection of early vitamin A deficiency.	5	Observ ational

		functional changes on the ocular surface can be detected by microscopic examination of PAS-hematoxylin stained epithelial cells obtained by briefly applying a cellulose acetate filter paper strip.				
Dietary Reference Intakes for Vitamin A, Vitamin K, Arsenic, Boron, Chromium, Copper, Iodine, Iron, Manganese, Molybdenum, Nickel, Silicon, Vanadium, and Zinc	Selection of indicators for estimating the requirement for Vitamin A	Before the clinical onset of xerophthalmia, mild vitamin A deficiency leads to early keratinizing metaplasia and losses of mucinsecreting goblet cells on the bulbar surface of the conjunctiva of the eye. These functional changes on the ocular surface can be detected by microscopic examination of PAS-hematoxylin stained epithelial cells obtained by briefly applying a cellulose acetate filter paper strip.	Assessment of vitamin A status by a disk applicator for conjunctival impression cytology [119]	Conjunctival impression cytology was performed on 236 Indonesian preschool children, half of whom had mild xerophthalmia and half of whom were age-matched controls. We devised an applicator that applies a paper disk of fixed area to the conjunctiva with even pressure. The disk applicator was used to collect impression cytology specimens from the temporal bulbar conjunctiva of one eye while the original strip technique was used on the other eye. Mean (±SD) serum retinol values for children with normal and abnormal discs were 22.0 ±8.6 µg/dL and 18.0 ±7.2 µg/dL *P*<.0001). Mean serum values for normal and abnormal strips were 21.7 ±8.6 µg/dL and 19.0 ±7.7 µg/dL (*P*<.03). Specimens obtained with the new disk applicator corresponded more closely with serum vitamin A levels and therefore vitamin A status than those obtained with the traditional strip technique.	5	Observational
Dietary Reference Intakes for Vitamin A, Vitamin K,	Selection of indicators for estimating the	An alternative approach involves transferring cell	Prevalence of malnutrition and vitamin A	The evaluation of the prevalence of ophthalmological diseases and vitamin A deficiency in Senegalese preschool-aged children enabled us to confirm the method of impression cytology with transfer (ICT) and to assess the impact of	5	Oberva tional

Arsenic, Boron, Chromium, Copper, Iodine, Iron, Manganese, Molybdenum, Nickel, Silicon, Vanadium, and Zinc	requirement for Vitamin A	specimens from the filter paper to a glass slide before staining and examination	deficiency in the Diourbel, Fatick and Kaolack regions of Senegal: Feasibility of the method of impression cytology with transfer [120]	ophthalmological diseases on the cytological appearance of conjunctival cells. A simplification of the ICT method consisted of transferring cells present on a strip of paper to a glass slide by finger pressure. Harris-Schorr staining of the sample papers confirmed the results obtained by the transfer. Inflammatory trachoma influenced the results of the test. This simple method will prove very helpful for detection of vitamin A deficiency in children in underdeveloped areas.		
Dietary Reference Intakes for Vitamin A, Vitamin K, Arsenic, Boron, Chromium, Copper, Iodine, Iron, Manganese, Molybdenum, Nickel, Silicon, Vanadium, and Zinc	Selection of indicators for estimating the requirement for Vitamin A	Several human studies have linked impairment in immunity to low plasma or serum vitamin A concentrations	Depressed immune response to tetanus in children with vitamin A deficiency [121]	A randomized, double-masked, placebo-controlled clinical trial was conducted with 236 preschool children, age 3-6 y, in Indonesia to assess immune status in mild vitamin A deficiency. The immune response to tetanus immunization was used as a measure of immune competence. Clinically normal children (n = 118) and children with mild xerophthalmia (n = 118) were randomly assigned to receive oral vitamin A (60,000 micrograms retinol equivalent) or placebo treatment for a total of four study groups. Two weeks after treatment, children were immunized with diphtheria-pertussis-tetanus vaccine. The immunoglobulin G (IgG) responses to tetanus at baseline and 3 wk following immunization were measured by ELISA. After adjusting for previous tetanus immunization, clinically normal and xerophthalmic children receiving vitamin A had a significantly greater IgG response to tetanus than clinically normal and xerophthalmic children receiving placebo (P less than 0.05). These results suggest that children with mild vitamin A deficiency have a relative immune depression compared with children who have been supplemented to normal vitamin A levels.	2	RCT
Dietary Reference Intakes for Vitamin A, Vitamin K, Arsenic, Boron, Chromium, Copper, Iodine, Iron, Manganese, Molybdenum, Nickel, Silicon, Vanadium, and Zinc	Selection of indicators for estimating the requirement for Vitamin A	Several human studies have linked impairment in immunity to low plasma or serum vitamin A concentrations	Vitamin A deficiency and T-cell subpopulations in children with meningococcal disease [122]	Although group A meningococcal disease is a major cause of child morbidity and mortality in sub-Saharan Africa, little is known about vitamin A status and T-cell subpopulations in affected children. A prospective study of vitamin A levels and T-cell subpopulations was conducted in 41 children hospitalized for meningococcal meningitis in Butare, Rwanda, during an epidemic from September through November, 1992. The mean age of cases was 3.6 +/- 2.7 years (range 0.5-16 years). The case-fatality rate was 20 per cent; 73 per cent of the children had serum vitamin A levels consistent with subclinical deficiency (< 0.7 mumol/l), and 27 per cent had levels consistent with severe deficiency (< 0.35 mumol/l). Mean CD4 per cent was higher and CD8 per cent was lower among children with meningitis compared with known reference populations. These results suggest that meningococcal disease is characterized by T-cell subpopulation alterations and vitamin A deficiency.	5	Observ ational
Dietary Reference Intakes for Vitamin	Factors affecting the	In some studies, increasing the level	Serum vitamin A in kwashiorkor [123]	No abstract available	2	RCT

A, Vitamin K, Arsenic, Boron, Chromium, Copper, Iodine, Iron, Manganese, Molybdenum, Nickel, Silicon, Vanadium, and Zinc	Vitamin A requirement	of fat in a low fat diet has been shown to improve retinol and carotene absorption				
Dietary Reference Intakes for Vitamin A, Vitamin K, Arsenic, Boron, Chromium, Copper, Iodine, Iron, Manganese, Molybdenum, Nickel, Silicon, Vanadium, and Zinc	Factors affecting the Vitamin A requirement	In some studies, increasing the level of fat in a low fat diet has been shown to improve … Vitamin A nutriture	The effect of protein and fat supplements on vitamin A deficient children [124]	No abstract available	2	RCT
Dietary Reference Intakes for Vitamin A, Vitamin K, Arsenic, Boron, Chromium, Copper, Iodine, Iron, Manganese, Molybdenum, Nickel, Silicon, Vanadium, and Zinc	Factors affecting the Vitamin A requirement	Other studies, however, have not demonstrated a beneficial effect of fat on vitamin A absorption	Absorption of vitamin A by infants receiving fat-free or fat-containing dried skim milk formulas [125]	No abstract available	3a	Experimental - absorption
Dietary Reference Intakes for Vitamin A, Vitamin K, Arsenic, Boron, Chromium, Copper, Iodine, Iron, Manganese, Molybdenum, Nickel, Silicon, Vanadium, and Zinc	Factors affecting the Vitamin A requirement	For optimal carotenoid absorption, a number of research groups have demonstrated that dietary fat must be consumed along with carotenoids. Roels and coworkers (1958) reported that the	Carotene balances on boys in Ruanda where vitamin A deficiency is prevalent [126]	No abstract available	3a	Experimental - absorption

		addition of 18 g/day of olive oil improved carotene absorption from 5 to 25 percent.				
Dietary Reference Intakes for Vitamin A, Vitamin K, Arsenic, Boron, Chromium, Copper, Iodine, Iron, Manganese, Molybdenum, Nickel, Silicon, Vanadium, and Zinc	Factors affecting the Vitamin A requirement	Jayarajan and coworkers (1980) reported that the addition 5 g of fat to the diet significantly improved serum vitamin A concentrations among children after the consumption of a low fat vegetable diet. The addition of 10 g of fat did not improve serum vitamin A concentrations any more than did 5 g of fat.	Effect of dietary fat on absorption of β-carotene from green leafy vegetables in children [127]	No abstract available	3a	Experimental - absorption
Dietary Reference Intakes for Vitamin A, Vitamin K, Arsenic, Boron, Chromium, Copper, Iodine, Iron, Manganese, Molybdenum, Nickel, Silicon, Vanadium, and Zinc	Factors affecting the Vitamin A requirement	Malabsorption of vitamin A is also associated with intestinal parasitism	Malabsorption of water miscible vitamin A in children with giardiasis and ascariasis [128]	Vitamin A absorption was studied using a water-miscible oral preparation of vitamin A in 19 children ages 1 1/2 to 9 years old with giardiasis and/or ascariasis, both before and after their eradication with appropriate therapy, and in three children without parasites. Marked impairment of vitamin A absorption was noted when administered in a water miscible form in children with 1) combined infection with Giardia lamblia and Ascaris lumbricoides, 2) giardiasis alone, and 3) in a proportion of children with ascariasis alone. In children with both giardiasis and ascarasis eradication of the infections promptly lead to a significant improvement in vitamin A absorption and restored it to normal. Children with giardiasis alone also showed improved vitamin A absorption after therapy. In children with ascariasis alone successful therpay did not lead to a statistically significant improvement.	3a	Experimental - absorption
Dietary Reference Intakes for Vitamin A, Vitamin K,	Factors affecting the	Malabsorption of vitamin A is also associated with	Absorption of vitamin A in	Absorption of labelled vitamin A was studied in normal children and in children with round-worm infestation. Absorption of the vitamin was significantly low in children with ascariasis. In two subjects, in whom the studies were repeated,	3a	Experimental -

Arsenic, Boron, Chromium, Copper, Iodine, Iron, Manganese, Molybdenum, Nickel, Silicon, Vanadium, and Zinc	Vitamin A requirement	intestinal parasitism	children with ascariasis [129]	absorption of vitamin A was significantly increased after antihelminthic treatment. These results suggest that ascariasis may aggravate vitamin A deficiency by interfering with the absorption of the vitamin.		absorption
Dietary Reference Intakes for Vitamin A, Vitamin K, Arsenic, Boron, Chromium, Copper, Iodine, Iron, Manganese, Molybdenum, Nickel, Silicon, Vanadium, and Zinc	Factors affecting the Vitamin A requirement	The malabsorption of vitamin A that is observed in children with *Ascaris lumbricoides* infection was associated with an altered mucosal morphology that was reversed with deworming	Ascaris lumbricoides infectation associated with jejunal mucosal abnormalities [130]	No abstract available	5	Observational
Dietary Reference Intakes for Vitamin A, Vitamin K, Arsenic, Boron, Chromium, Copper, Iodine, Iron, Manganese, Molybdenum, Nickel, Silicon, Vanadium, and Zinc	Factors affecting the Vitamin A requirement	A direct correlation between hemoglobin and serum retinol concentrations has been observed	Interrelationship between vitamin A, iodine and iron status in schoolchildren in Shoa Region, central Ethiopia [131]	A total of 14,740 schoolchildren in seven provinces of Shoa Administrative Region in Central Ethiopia were surveyed for the prevalence of goitre, xerophthalmia and anaemia. Haemoglobin and packed cell volume were assessed in 966 children in one province while an in-depth study was conducted on 344 children in the same province and two others. Goitre, xerophthalmia (Bitot's spots) and clinical anaemia were observed in 34.2, 0.91 and 18.6% respectively of the children. Most biochemical variables were within the normal range while those of haemoglobin (Hb), mean corpuscular Hb concentration (MCHC) and urinary I excretion were lower, and mean corpuscular volume, mean corpuscular Hb (MCH), and immunoglobulins G and M were higher. Hb was strongly correlated with retinol, ferritin, MCHC, MCH, packed cell volume and erythrocyte count while retinol formed a triad with transthyretin (TTR) and retinol-binding protein (RBP) which were all correlated with one another. Total and free thyroxin and total and free triiodothyronine were positively correlated as were the concentrations of the total and free hormones. Thyrotropin (TSH) was negatively correlated with total and free thyroxin and positively correlated with free triiodothyronine. Thyroxin and triiodothyronine in both free and combined forms were all correlated with thyroxin-binding globulin which in turn was negatively correlated with the triad retinol, RBP and TTR. The triad was also negatively correlated with C-reactive protein. Urinary I excretion was positively associated with total thyroxin and negatively associated with TSH. The anaemia	5	Observational

				found was not nutritional in origin but due to the effect of infestation with intestinal parasites and malaria.		
Dietary Reference Intakes for Vitamin A, Vitamin K, Arsenic, Boron, Chromium, Copper, Iodine, Iron, Manganese, Molybdenum, Nickel, Silicon, Vanadium, and Zinc	Factors affecting the Vitamin A requirement	it has been suggested that zinc intake may positively affect vitamin A status only when individuals are moderately to severely protein-energy deficient	Effect of zinc supplementation on plasma levels of vitamin A and retinol-binding protein in malnourished children [132]	The relationship between plasma levels of vitamin A and zinc was studied in 45 children suffering from vitamin A deficiency and 20 children with protein-energy malnutrition. Thirty apparently normal children of the same age group were also studied for comparison. The mean levels of plasma vitamin A, retinol-binding protein and zinc were significantly lower in vitamin A-deficient children and in children with PEM, as compared to controls. Supplementation with 40 mg zinc daily for 5--10 days resulted in a significant increase in plasma vitamin A and RBP levels in children with PEM but not in the vitamin A-deficient group. There was, however, no correlation between plasma levels of vitamin A and zinc. The data suggest that in children with PEM, apart from deficiencies of protein and vitamin A, zinc deficiency may also contribute to the lowering of plasma vitamin A levels. They also suggest that in vitamin A-deficient children, without protein-energy malnutrition, zinc deficiency does not seem to have a role.	2	RCT
Dietary Reference Intakes for Vitamin A, Vitamin K, Arsenic, Boron, Chromium, Copper, Iodine, Iron, Manganese, Molybdenum, Nickel, Silicon, Vanadium, and Zinc	Method Used to Set the Adequate Intake	average concentration of vitamin A in human milk of 1.70 µmol/L (485 µg/L) during the first 6 months of lactation	Beta-carotene in breast milk and serum is increased after a single beta-carotene dose [133]	Normal lactating mothers were administered a single dose of 60 or 210 mg beta-carotene and changes in serum and milk retinol, alpha-tocopherol, and carotenoids were monitored for 8 d. Average serum beta-carotene concentrations increased 4.1- and 4.0-fold after the 60- and 210-mg doses, respectively. Milk beta-carotene concentrations increased 4.1- and 3.0-fold after the 60- and 210-mg doses, respectively. Maximum serum concentrations were reached 24 h after both supplements, although concentrations of milk beta-carotene continued to rise for 2-3 d. After 8 d, both serum and milk beta-carotene continued to rise for 2-3 d. After 8 d, both serum and milk beta-carotene concentrations remained about twofold higher than baseline concentrations. Increases in serum or milk beta-carotene concentrations were not dose-dependent. Initial serum and milk concentrations of beta-carotene predicted increases after supplementation, and increases in serum beta-carotene concentrations predicted those in milk. Concentrations of milk carotenoids were less than one-tenth their respective concentrations in serum. Lutein, beta-cryptoxanthin, lycopene, alpha-carotene, retinol, and alpha-tocopherol concentrations in serum or milk did not change significantly after beta-carotene supplementation. Retinol esters account for most of the retinol equivalents in the milk of well-nourished mothers. Initial and maximum concentrations of beta-carotene in serum and milk were strongly correlated for individual mothers. Collectively, the data showed that a single 60-mg supplement of beta-carotene sustained elevated beta-carotene concentrations in serum and milk for > 1 wk in normal mothers but did not affect concentrations of other major carotenoids, retinol, or alpha-tocopherol.	3a	Experimental - absorption

Dietary Reference Intakes for Vitamin A, Vitamin K, Arsenic, Boron, Chromium, Copper, Iodine, Iron, Manganese, Molybdenum, Nickel, Silicon, Vanadium, and Zinc	Method Used to Set the Adequate Intake	average concentration of vitamin A in human milk of 1.70 µmol/L (485 µg/L) during the first 6 months of lactation	Kinetics of the response of milk and serum beta-carotene to daily beta-carotene supplementation in healthy, lactating women [134]	Changes in concentrations of milk and serum carotenoids, retinol, and alpha-tocopherol of five healthy, well-nourished, lactating women were measured over a 28-d supplementation trial with 30 mg beta-carotene and for 4 wk thereafter. Beta-carotene supplementation increased mean beta-carotene concentrations in milk and serum 6.4- and 7.4-fold, respectively. Concentrations of other major carotenoids, retinol, and alpha-tocopherol did not change substantially in either milk or serum. Uptake of beta-carotene into both serum and milk followed apparent first-order kinetics, occurring more rapidly into serum (t(1/2) = 5.5 d) than into milk (t(1/2) = 9 d). After supplementation, milk and serum beta-carotene concentrations decayed slowly, reaching approximately twofold initial concentrations by 4 wk. Kinetics of uptake and decay in milk beta-carotene concentrations paralleled those in serum. The data show that short-term supplementation of healthy, lactating mothers with purified beta-carotene at approximately fivefold the average daily dietary intake substantially increased milk and serum beta-carotene concentrations while not interfering with concentrations of other carotenoids, retinol, or alpha-tocopherol in milk or serum. Thus, an increased intake of beta-carotene by healthy, lactating women increases the supply of milk beta-carotene available to their breast-feeding infants.	3a	Experimental - absorption
Dietary Reference Intakes for Vitamin A, Vitamin K, Arsenic, Boron, Chromium, Copper, Iodine, Iron, Manganese, Molybdenum, Nickel, Silicon, Vanadium, and Zinc	Method Used to Set the Adequate Intake	Furthermore, the urinary excretion of vitamin A is increased with infection, and especially with fever	Urinary excretion of retinol in children with acute diarrhea [135]	Acute infections of childhood are associated with an increased of xerophthalmia, apparently due to depletion of vitamin A stores. The mechanism responsible for this is not known. Recently, it has been reported that severe infections in adult patients (ie, sepsis and pneumonia) result in excretion of large quantities of retinol in the urine. In 44 children hospitalized for treatment of acute diarrhea we found mean urinary excretions of 1.44 mumol retinol/24 h on day 1 of hospitalization, 0.62 mumol retinol/24 h on day 2, and 0.23 mumol/24 h on day 3. Healthy control subjects matched for age did not excrete measurable amounts of retinol in the urine. Retinol excretion was associated strongly with rotavirus diarrhea and presence of fever. Furthermore, serum retinol concentration was negatively associated with duration of diarrhea before hospitalization, suggesting that urinary excretion of retinol may be an important contributor to vitamin A depletion.	3b	Experimental – excretion
Dietary Reference Intakes for Vitamin A, Vitamin K, Arsenic, Boron, Chromium, Copper, Iodine, Iron, Manganese, Molybdenum,	Method Used to Set the Adequate Intake	Protein synthesis generally, and specifically retinol binding protein synthesis, is reduced with severe protein energy malnutrition	The early changes in retinol-binding protein and prealbumin concentrations in plasma of protein-energy malnourished	1. Changes in total retinol-binding protein (RBP), the holoprotein (holoRBP) and prealbumin (PA) concentrations have been monitored in plasma of thirty protein- and vitamin A-deficient preschool children from within a few hours up to 7 weeks after treatment with retinol and a good-quality protein diet. 2. The children were classified into groups according to nutritional status as having either kwashiorkor, marasmus-kwashiorkor or marasmus, and given formula diets whose protein and energy contents increased stepwise from 1 g and 105 kJ/kg body-weight respectively up to 4 g and 733 kJ/kg body-weight after 4	2	RCT

Nickel, Silicon, Vanadium, and Zinc		(PEM) (marasmus and kwashiorkor), and therefore release of retinol from the liver (assuming stores are present) is also reduced	children after treatment with retinol and an improved diet [136]	weeks. Retinol was administered in the forms of retinyl palmitate either orally or intramuscularly. 3. PA and total RBP were determined by electroimmunoassay procedures and the holoRBP by its fluorescence after separation from other plasma proteins. 4. RBP in plasma of the vitamin A-deficient child is largely denatured and incapable of binding administered retinol, which must first be taken up by the liver before native holoRBP is released. An increased pool of native apoprotein accumulates in the liver during vitamin A deficiency which is released into plasma quickly after retinol uptake to form peak concentrations of total and holoRBP approximately 3 h after dosing intramuscularly and 6 h orally. 5. The accumulated pool of RBP was highest in livers from the marasmus group and lowest in those from the kwashiorkor group, reflecting their relative capacities to synthesize plasma proteins. 6. The mean plasma concentrations of total and holoRBP for the various groups were minimal 24-48 h after dosing with retinol and then improved almost linearly over the following week. 7. Mean plasma PA concentrations of the various groups on admission were also in order of the severity of their malnutrition. There was little or no change in this protein concentration over the first 24 h after dosing with retinol, but thereafter the mean values rose almost linearly over 2 weeks. Albumin on the other hand changed little during the first week. The results show that PA is the more sensitive measurement of protein nutritional status.		
Dietary Reference Intakes for Vitamin A, Vitamin K, Arsenic, Boron, Chromium, Copper, Iodine, Iron, Manganese, Molybdenum, Nickel, Silicon, Vanadium, and Zinc	Method Used to Set the Adequate Intake					
Dietary Reference Intakes for Vitamin A, Vitamin K, Arsenic, Boron, Chromium, Copper, Iodine, Iron, Manganese, Molybdenum, Nickel, Silicon, Vanadium, and Zinc	Method Used to Set the Adequate Intake	Three major intervention trials have been conducted in developing countries to evaluate the efficacy of provitamin A carotenoids in	Children's consumption of dark green, leafy vegetables with added fat enhances serum retinol [137]	A randomized, double-blind, controlled study was conducted to determine whether the consumption of leafy vegetables by preschool children would enhance their serum vitamin A concentration to acceptable levels. Preschool children (n = 519; 2.5–6 y) in Saboba, northern Ghana, were randomly assigned to five feeding groups, differing essentially in the amount of fat and β-carotene, fed once per d, 7 d per wk, for 3 mo. Serum retinol levels, anthropometric measurements, hemoglobin, rapid turnover proteins (pre-albumin and retinol-binding protein), worm infestation (stool examinations) and level of acute and chronic infection (serum C-reactive protein and acid glycoprotein) were determined before and after study. Relative to the baseline serum retinol values,	2	RCT

		maintaining or improving vitamin A status		consumption of dark green, leafy vegetables (Manihot sp. and Ceiba sp.) with fat (10 g/100 g) significantly ($P < 0.05$) enhanced serum retinol; consequently, the percentage of children with adequate retinol status increased from 28.2–48.2% after feeding ($P < 0.05$). There were no significant differences among groups, ages or pre- versus post-anthropometric measurements, hemoglobin concentration, or levels of worm infestation. The importance of these findings in alleviating and/or controlling vitamin A deficiency in developing countries is discussed.		
Dietary Reference Intakes for Vitamin A, Vitamin K, Arsenic, Boron, Chromium, Copper, Iodine, Iron, Manganese, Molybdenum, Nickel, Silicon, Vanadium, and Zinc	Method Used to Set the Adequate Intake	iron supplementation improved vitamin A status in humans	Iron and zinc supplementation improves indicators of vitamin A status of Mexican preschoolers [138]	Background: The coexistence of multiple micronutrient deficiencies is a widespread public health problem in many regions of the world. Interactions between zinc deficiency and vitamin A metabolism have been reported but no longitudinal studies have evaluated the effect of iron deficiency on vitamin A. Objective: The objective of this study was to investigate the effect of supplementation with iron, zinc, or both on vitamin A and its metabolically related proteins retinol binding protein (RBP) and transthyretin. Design: The study was a longitudinal, double-blind, placebo-controlled trial in which 219 rural Mexican children aged 18-36 mo were randomly assigned to receive 20 mg Zn/d, 20 mg Fe/d, 20 mg Zn/d plus 20 mg Fe/d, or placebo. Results: Six months after supplementation, plasma retinol increased in all supplemented groups. Compared with placebo, zinc supplementation was associated with significantly higher plasma retinol and transthyretin but the increase in RBP was not significant. Iron supplementation significantly increased plasma retinol, RBP, and transthyretin. Supplementation with zinc plus iron significantly increased plasma retinol but not RBP or transthyretin. Children deficient in zinc, iron, or vitamin A (as indicated by nutrient plasma concentration) at the beginning of the study had a significantly greater increase in retinol than did children with adequate nutrient status. Conclusions: Supplementation with zinc, iron, or both improved indicators of vitamin A status. The results of this study agree with previous observations of a metabolic interaction between zinc and vitamin A and suggest an interaction between iron and vitamin A metabolism.	2	RCT
Nordic Council of Ministers. Nordic Nutrition Recommendations 2012.	Physiology and metabolism	A series of epidemiological and intervention studies in children living under poor socioeconomic conditions have documented a relationship between poor	Vitamin A for preventing secondary infections in children with measles--a systematic review [139]	The objective of the present study was to determine whether vitamin A prevents pneumonia, diarrhoea and other infections in children with measles. A meta-analysis was carried out of randomized controlled trials identified through a systematic search of the medical literature for studies that used vitamin A to treat measles. A total of 492 children, aged from 6 months to 13 years, were supplemented with vitamin A, and 536 children were given placebo in six trials, five of which were conducted in hospitals and one in a community setting. The main outcome measures were: incidence of pneumonia, diarrhoea, croup, and otitis media; and duration of pneumonia, diarrhoea, fever and hospitalization. There was no significant reduction in the incidence of pneumonia or diarrhoea	1	Systematic review

		vitamin A supply and increased rates and severity of infections as well as mortality related to infectious diseases such as measles		but there was a 47 per cent reduction in the incidence of croup (RR = 0.53; 95 per cent CI = 0.29-0.89) in children who were treated with 200 000 IU of vitamin A on 2 consecutive days. Only one study reported a 74 per cent reduction in the incidence of otitis media (RR = 0.26 95 per cent CI = 0.05-0.92). There was a statistically significant decrease in the duration of diarrhoea, pneumonia, hospital stay and fever in individual studies. It was concluded that vitamin A does have a beneficial effect on morbidity associated with measles and should be used as a treatment for hospitalized measles cases.		
Apports nutritionnels conseillés pour la population française, 3e éd. (retirage 2018)<br><br>Recommended nutritional intake for the French population, 3rd ed. (reprint 2018)	Second part. 9.1.2.2 hypovitamin A	Vitamin A deficiency is one of the major public health problems in developing countries. It results in characteristic attacks of the ocular sphere: hemeralopia *, then xerophthalmia * (alterations of the cornea) and irreversible blindness. It is accompanied by a significant drop in immune defenses, especially in young children, for whom complications from infectious diseases (diarrhea, measles, respiratory infections) can be extremely serious (Humphrey et al., 1992)	Vitamin A deficiency and attributable mortality among under-5-year-olds [140]	Reported are estimates of the prevalence in developing countries of physiologically significant vitamin A deficiency and the number of attributable deaths. The WHO classification of countries by the severity and extent of xerophthalmia was used to categorize developing countries by likely risk of subclinical vitamin A deficiency. Using vital statistics compiled by UNICEF, we derived population figures and mortality rates for under-5-year-olds. The findings of vitamin A supplementation trials were applied to populations at-risk of endemic vitamin A deficiency to estimate the potential impact of improved vitamin A nutriture in reducing mortality during preschool years. Worldwide, over 124 million children are estimated to be vitamin A deficient. Improved vitamin A nutriture would be expected to prevent approximately 1-2 million deaths annually among children aged 1-4 years. An additional 0.25-0.5 million deaths may be averted if improved vitamin A nutriture can be achieved during the latter half of infancy. Improved vitamin A nutriture alone could prevent 1.3-2.5 million of the nearly 8 million late infancy and preschool-age child deaths that occur each year in the highest-risk developing countries.	6	non-system atic review
Apports nutritionnels conseillés pour la population	Third part. Recommended nutritional intake for	The Directive of 14 May 1991 of the Commission of the European	Commission Directive 91/321/EEC of 14 May 1991 on	No abstract available.		Legal directiv e

française, 3e éd. (retirage 2018)<br><br>Recommended nutritional intake for the French population, 3rd ed. (reprint 2018)	different population groups: Infants, children and adolescents.<br>7.1 Vitamin A	Communities (91/321/EEC) fixed the composition of infant formulas and follow-on formulas at a minimum of 60 and a maximum of 180 retinol equivalents per 100 kcal (Directive, 1991).	infant formulae and follow-on formulae [141]			
Apports nutritionnels conseillés pour la population française, 3e éd. (retirage 2018)<br><br>Recommended nutritional intake for the French population, 3rd ed. (reprint 2018)	Third part. Recommended nutritional intake for different population groups: Infants, children and adolescents.<br>7.1 Vitamin A	Above one year, the recommendations are extrapolated from adult data: 400 to 500 retinol equivalents/day (ER/d) up to 10 years, 550 to 800 ER/d thereafter (table 15) (FNB , 1989; CSAH, 1994).	Recommended dietary allowances [142]	RDAs are defined in Chapter 2 as the levels of intake of essential nutrients that, on the basis of scientific knowledge, are judged by the Food and Nutrition Board to be adequate to meet the known nutrient needs of practically all health persons. This definition has remained virtually unchanged since 1974...	6	non-system atic review
Apports nutritionnels conseillés pour la population française, 3e éd. (retirage 2018)<br><br>Recommended nutritional intake for the French population, 3rd ed. (reprint 2018)	Third part. Recommended nutritional intake for different population groups: Infants, children and adolescents.<br>7.1 Vitamin A	Above one year, the recommendations are extrapolated from adult data: 400 to 500 ER/d up to 10 years, 550 to 800 ER/d thereafter (table 15) (FNB , 1989; CSAH, 1994).	Reports of the scientific committee for food [143]	No summary I could copy over quickly, but says this in Introduction:<br><br>"The Committee considers that information is inadequate to give Average Requirements or Lowest Threshold Intakes for children and is limiting itself to giving Population Reference Intakes"<br><br>https://op.europa.eu/fr/publication-detail/-/publication/b0c0ebc9-8642-4bad-bb3a-30d952ecbb86/language-en	6	non-system atic review
Dietary Reference Values for Food Energy and Nutrients for the	Requirements	The recent FAO/ WHO Expert Group considered that a daily intake of 350 ttg retinol	Requirements of Vitamin A, Iron, Folate and Vitamin B12 [144]	No abstract available.		Publish ed guideli nes

United Kingdom (1991)		equivalents would meet the needs of all healthy infants and allow the building and maintaining of sufficient liver stores.				
Nutrient and energy intakes for the European Community. Reports of the Scientific Committee for Food, 31st Series. 1993.	Children	Most recommendations for formula-fed infants are based on the amounts in breast milk, e.g. the most recent FAO/WHO value of 350 µg retinol equivalents/d				
Dietary Reference Values for Food Energy and Nutrients for the United Kingdom (1991)	Requirements	Intakes lower than those suggested here maintained satisfactory plasma retinol concentrations in preschool children in India.	Observations on vitamin A requirement [145]	No abstract available.	6	Non-systematic review
Nutrient and energy intakes for the European Community. Reports of the Scientific Committee for Food, 31st Series. 1993.	Children	Reddy has reported that a daily intake of about 300 µg will meet the requirements of pre-school children.				
Nutrient and energy intakes for the European Community. Reports of the Scientific	Children	This is likely to be an overestimate as no breast-fed infants ever show signs of A deficiency, even on	Chemical composition of human milk in poor Indian women [146]	No abstract available.	5	Observational

Committee for Food, 31st Series. 1993.		intakes of 100-200 µg/d				
Nutrient and energy intakes for the European Community. Reports of the Scientific Committee for Food, 31st Series. 1993.	Children	This is likely to be an overestimate as no breast-fed infants ever show signs of A deficiency, even on intakes of 100-200 µg/d	Evaluation of lactational performance of Navajo Women [147]	The effect of suboptimal maternal nutrition on lactational performance of 23 Navajo women was studied in terms of milk volume, milk composition, and infant growth. The mean milk volume produced by 10 Navajo women was 634 +/- 113 mg/24 h after approximately 1 month of lactation. The content of protein, lactose, and lipid were within normal limits. Retinol and carotene content were 32.9 +/- 15.7 and 19.7 +/- 6.3 microgram/dl, respectively. Milk folacin averaged 56.4 +/- 23.9 mg/ml. The mean contents of zinc, iron, and copper were 2.8 +/- 1.1, 0.8 +/- 0.6, and 0.3 +/- 0.2 mg/l, respectively. Despite evidence of suboptimal nutriture among these Navajo women, lactational performance was adequate in terms of infant growth, milk volume, and milk composition with the exception of vitamin A which was lower than normal.	5	Observ ational

D-A-CH. Referenzwerte für die Nährstoffzufuhr. 2015.[148] References from the D-A-CH guidelines relating to vitamin A requirements for infants and children include the following: [4, 22, 24, 25, 133, 149-162]

Supplementary Table S7. Dietary Reference Values and recommendations cited in existing guidelines.

Scientific Opinion on Dietary Reference Values for Iron EFSA Journal 2015;13(10):4254

- D-A-CH (Deutsche Gesellschaft für Ernährung, Österreichische Gesellschaft für Ernährung, Schweizerische Gesellschaft für Ernährung), 2015. Referenzwerte für die Nährstoffzufuhr. 2. Auflage, 1. Ausgabe. DGE, Bonn, Germany.
- Nordic Council of Ministers, 2014. Nordic Nutrition Recommendations 2012. Integrating nutrition and physical activity. Copenhagen, Denmark, 627 pp.
- WHO/FAO (World Health Organization/Food and Agriculture Organization of the United Nations), 2004. Vitamin and mineral requirements in human nutrition: report of a Joint FAO/WHO Expert Consultation. Bangkok, Thailand, 21-30 September 1998. 341 pp.
- SCF (Scientific Committee for Food), 1993. Nutrient and energy intakes for the European Community. Reports of the Scientific Committee for Food, 31st Series. Food - Science and Technique, European Commission, Luxembourg, 248 pp.
- Afssa (Agence française de sécurité sanitaire des aliments), 2001. Apports nutritionnels conseillés pour la population française. Editions Tec&Doc, Paris, France, 605 pp.
- IOM (Institute of Medicine), 2001. Dietary Reference Intakes for vitamin A, vitamin K, arsenic, boron, chromium, copper, iodine, iron, manganese, molybdenum, nickel, silicon, vanadium, and zinc. Food and Nutrition Board. National Academy Press, Washington, DC, USA, 797 pp.
- Netherlands Food and Nutrition Council, 1992. Recommended Dietary Allowances 1989 in the Netherlands. 115 pp.
- DH (Department of Health), 1991. Dietary Reference Values for food energy and nutrients for the United Kingdom. Report of the Panel on Dietary Reference Values of the Committee on Medical Aspects of Food Policy. HMSO, London, UK, 212 pp.

Vitamin and mineral requirements in human nutrition. 2004: World Health Organization

- Food and Nutrition Board. Dietary reference intakes for calcium, phosphorus, magnesium, vitamin D, and fluoride. Washington, DC, National Academy Press, 1997.
- Scientific Review Committee. Nutrition recommendations: Health and Welfare, Canada. Report of the Scientific Review Committee. Ottawa, Supply and Services, 1992.
- Department of Health. Dietary reference values for food energy and nutrients for the United Kingdom. London, Her Majesty's Stationery Office, 1991 (Report on Health and Social Subjects, No. 41).
- Subcommittee on the Tenth Edition of the Recommended Dietary Allowances, Food and Nutrition Board. Recommended dietary allowances, 10th ed. Washington, DC, National Academy Press, 1989.
- Reference nutrient intakes for the European Community: a report of the Scientific Committee for Food. Brussels, Commission of the European Communities, 1993.

Supplementary Table S8. Methodologies used in setting previous upper limits for vitamin A in infants and children

The text below is taken from the methodology sections of the earlier guidelines that developed upper limits for vitamin A, giving some insight to how previous ULs were set for infants and children. Please note that these sections (text and tables) are largely “cut and paste” – for information only.

Dietary Reference Intakes for Vitamin A, Vitamin K, Arsenic, Boron, Chromium, Copper, Iodine, Iron, Manganese, Molybdenum, Nickel, Silicon, Vanadium, and Zinc[4]

Tolerable upper intake limits

Data Selection. Case reports of hypervitaminosis A in infants were used to identify a LOAEL and derive a UL. Data were not available to identify a NOAEL.

Identification of a LOAEL. A LOAEL of 6,460 µg/day of vitamin A (which was rounded to 6,000 µg/day) was identified by averaging the lowest doses of four case reports (Persson et al., 1965). Four cases of hypervitaminosis A occurred after doses of 5,500 to 6,750 µg/day of vitamin A for 1 to 3 months (Table 4-10). The age of onset of symptoms ranged from 2.5 to 5.5 months and included anorexia, hyperirritability, occipital edema, pronounced craniotabes, bulging fontanels, increased intracranial pressure, and skin lesions and desquamation. The lowest dose associated with a bulging fontanel involved a 4-month-old girl given a daily dose of 24 drops of AD-vimin (about 5,500 µg of vitamin A) for 3 months. Her fontanels bulged 0.5 centimeters above the plane of the skull. The other three cases involved a dose of 6,750 µg/day of vitamin A for 1 to 2.5 months. Increased intracranial pressure and bulging fontanels were observed in these cases as well. Other effects observed at the higher dose included anorexia, hyperirritability, occipital edema, pronounced craniotabes, skin lesions, skin desquamation, epiphyseal line changes, and cortical hyperostosis on x-rays.

Uncertainty Assessment. A UF of 10 was selected to account for the uncertainty of extrapolating a LOAEL to a NOAEL for a nonsevere and reversible effect (i.e., bulging fontanel) and the interindividual variability in sensitivity.

Derivation of a UL. The LOAEL of 6,000 µg/day was divided by a UF of 10 to calculate a UL of 600 µg/day of preformed vitamin A for infants.

Children and Adolescent Boys. There are limited case report data of hypervitaminosis A (e.g., bulging anterior fontanels, increased intracranial pressure, hair loss, increased suture markings on the skull, and periosteal new bone formation) in children and adolescents after doses ranging from 7,000 µg/day in young children to 15,000 µg/day in older children and adolescents (Farris and Erdman, 1982; Siegel and Spackman, 1972; Smith and Goodman, 1976). Given the dearth of information and the need for conservativism, the UL values for children and adolescents are extrapolated from those established for adults. Thus, the adult UL of 3,000 µg/day of preformed vitamin A was adjusted for children and adolescents on the basis of relative body weight as described in Chapter 2 with use of reference weights from Chapter 1 (Table 1-1). Values have been rounded.

Supplementary Table S9. References relating to setting upper limits for vitamin A in infants and children

Guideline - title	Guideline – linking section	Guideline – linking text	Study - title and Endnote link	Study - abstract	Study - ref	Study - type
Vitamin and mineral requirements in human nutrition. 2004: World Health Organization	Toxicity	Infants, including neonates, administered single doses equivalent to 15000–30000µg retinol (50000–100000IU) in oil generally show no adverse symptoms.	Neonatal vitamin A supplementation: effect on development and growth at 3y of age [163]	We reported recently that neonatal supplementation with 52 micromol vitamin A reduced infant mortality by 64%; acute side effects were limited to a 3% excess rate of a bulging fontanelle. The current study was conducted to identify developmental changes at 3 y of age associated with neonatal vitamin A supplementation or a bulging fontanelle. Children who had a bulging fontanelle (n = 91) and 432 children who had normal fontanelles after receiving vitamin A or placebo were evaluated with the Bayley Scales of Infant Development. Mean scores for the mental, psychomotor, and behavioral rating scale (BRS) plus 3 subscales of the BRS were not significantly different for treatment-fontanelle-specific groups. In regression models predicting each score, a bulging fontanelle had a small negative effect in all models; when 1 child who was injured from birth was removed from the analysis the effect of a bulging fontanelle was not significant in any model (P > or = 0.35). Vitamin A supplementation had a small beneficial effect on all developmental scores, which was significant for one of the BRS subscales (orientation-engagement) and also for a second (motor quality) when the outlier child was removed. Compared with children with normal fontanelles in the placebo group, children with a bulging fontanelle in the vitamin A group tended to grow less (-0.5 cm, P = 0.33), whereas those with normal fontanelles in the vitamin A group grew significantly more (0.68 cm, P < 0.05), over the first 3 y of life. This study provides no evidence that neonatal vitamin A supplementation is associated with biologically significant adverse growth or developmental sequelae.	2	RCT
Vitamin and mineral requirements in human nutrition. 2004: World Health Organization	Toxicity	An increase in bulging fontanelles occurred in infants under 6 months of age in one endemically deficient	Bulging fontanelle after supplementation with 25,000IU vitamin A in infancy using immunization contacts [164]	To evaluate the safety of vitamin A supplementation in early infancy using DPT/OPV immunization contracts, a double-blind, randomized, placebo-controlled trial was conducted in Bangladesh. One hundred and sixty-seven infants received three doses of either 25,000 IU of vitamin A or a placebo at about 6.5, 11.8 and 17.0 weeks of age. Trained physicians examined each of the infants on days 1, 2, 3 and 8 after supplementation. Nine	2	RCT

		population given two or more doses of 7500μg or 15000μg preformed vitamin A in oil		infants (10.5%) supplemented with vitamin A had episodes of bulging of the fontanelle compared with two infants (2.5%) in the placebo group ($p < 0.05$). Twelve of the 14 episodes occurred in infants supplemented with vitamin A. Of these 12 episodes, none occurred with the first dose, 3 occurred with the second and 9 with the third dose. The higher incidence of bulging of the fontanelle in the vitamin A group relative to the placebo group and its temporal association with the vitamin A doses are suggestive of a causal association. The finding that increased numbers of vitamin A doses were associated with a higher probability of bulging of the fontanelle suggests a cumulative effect.		
Vitamin and mineral requirements in human nutrition. 2004: World Health Organization	Toxicity	An increase in bulging fontanelles occurred in infants under 6 months of age in one endemically deficient population given two or more doses of 7500μg or 15000μg preformed vitamin A in oil	Acute toxicity of vitamin A given with vaccines in Infancy [165]	A double-blind, randomised, placebo-controlled trial was conducted to evaluate the safety and toxicity of vitamin A supplementation within the Expanded Programme on Immunisation (EPI) in rural Bangladesh. 191 infants received 3 doses of either 50,000 IU of vitamin A or placebo at about 1.5, 2.5, and 3.5 months and were examined on days 1, 2, 3, and 8 after supplementation. 11 infants (11.5%) supplemented with vitamin A had episodes of bulging of the fontanelle as opposed to 1 (1%) in the placebo group. 16 of the 17 events occurred in the vitamin A supplemented group. No other side effects were noted. There was a tendency towards a cumulative effect of toxicity with increasing doses.	2	RCT
Vitamin and mineral requirements in human nutrition. 2004: World Health Organization	Toxicity	other large-scale controlled clinical trials have not reported increased bulging after three doses of 7500μg given with diphtheria-pertussis-tetanus immunizations at about 6, 10, and 14 weeks of age	Randomised trial to assess benefits and safety of vitamin A supplementation linked to immunisation in early infancy [166]	Background The benefits and safety of vitamin A supplementation linked to immunisation in infancy need to be assessed before it can be widely recommended. We assessed the safety and benefits of maternal postpartum and infant vitamin A supplementation administered with each of the three diphtheria-tetanus-pertussis (DPT) and poliomyelitis immunisations and with a fourth dose with measles immunisation. Methods From January, 1995, we enrolled 9424 mother-infant pairs from Ghana, India, and Peru in this randomised, double-blind, placebo-controlled trial. 4716 mothers of infants in the vitamin A group received 200 000 IU vitamin A, and their infants were given 25 000 IU vitamin A with each of the first three doses of DPT/poliomyelitis immunisation at 6, 10, and 14 weeks. In the control group, 4708 mothers and their infants received placebo at the same times. At 9 months, with measles immunisation,	2	RCT

				infants in the vitamin A group were given a further dose of 25 000 IU and those in the control group received 100 000 IU vitamin A. Infants were followed up to age 12 months. The primary outcome measures were vitamin A status, signs of acute toxic effects, anthropometric indicators, and severe morbidity. Analysis was by intention to treat. Findings 3933 (93%) of the eligible 4212 infants on vitamin A and 3938 (93%) of the eligible 4227 controls received all four study doses. At the 6-month follow-up, there was a small decrease in vitamin A deficiency in the vitamin A group compared with controls (serum retinol ⩽0·70 µmol/L 101 [29·9%] vs 122 [37·1%; 95% CI of the difference−14·3% to −0·2%]). This effect was no longer apparent at 9 and 12 months. There were no significant between-group differences in mortality throughout the study. The rate ratio to compare all deaths up to age 9 months in the two groups was 0·96 (95% CI 0·73 to 1·27). Fewer than 1% of the infants had bulging fontanelle. The intervention had no effect on anthropometric status, or on overall or severe morbidity. Interpretation The trial confirmed the safety of the intervention, but shows no sustained benefits in terms of vitamin A status beyond age 6 months or infant morbidity.		
Vitamin and mineral requirements in human nutrition. 2004: World Health Organization	Toxicity	No effects were detected at 3 years of age that related to transient vitamin A-induced bulging that had occurred before 6 months of age	Long-term effect of vitamin A with vaccines [167]	No abstract available.	2	RCT
Vitamin and mineral requirements in human nutrition. 2004: World Health Organization	Toxicity	Most children aged 1–6 years tolerate single oral doses of 60000µg (200000IU) vitamin A in oil at intervals of 4–6 months without adverse Symptoms. Occasionally diarrhoea or	Evaluation of vitamin A toxicity [168]	Toxicity has been associated with abuse of vitamin A supplements and with diets extremely high in preformed vitamin A. Consumption of 25,000-50,000 IU/d for periods of several months or more can produce multiple adverse effects. The lowest reported intakes causing toxicity have occurred in persons with liver function compromised by drugs, viral hepatitis, or protein-energy malnutrition. Certain drugs or other chemicals may markedly potentiate vitamin A toxicity in animals. Especially vulnerable groups include children, with adverse effects occurring with intakes as low as 1,500 IU.kg-1.d-1, and pregnant women, with birth defects being associated with maternal	6	Non-systematic review

		vomiting is reported but these symptoms are transient with no lasting sequelae. Older children seldom experience toxic symptoms unless they habitually ingest vitamin A in excess of 7500µg (25000IU) for prolonged periods of time		intakes as low as approximately 25,000 IU/d. The maternal dose threshold for birth defects cannot be identified from present data. An identifiable fraction of the population surveyed consumes vitamin A supplements at 25,000 IU/d and a few individuals consume much more. beta-Carotene is much less toxic than vitamin A.		
Dietary Reference Intakes for Vitamin A, Vitamin K, Arsenic, Boron, Chromium, Copper, Iodine, Iron, Manganese, Molybdenum, Nickel, Silicon, Vanadium, and Zinc	Hazard Identification	Acute toxicity is characterized by … bulging fontanel in infants	Chronic vitamin A intoxication during the first half year of life [169]	No abstract available	5	Observational
Dietary Reference Intakes for Vitamin A, Vitamin K, Arsenic, Boron, Chromium, Copper, Iodine, Iron, Manganese, Molybdenum, Nickel, Silicon, Vanadium, and Zinc	Hazard Identification	These are usually transient effects involving single or short-term large doses of greater than or equal to 150,000µg in adults and proportionately less in children	Safety of vitamin A [170]	Vitamin A adequacy is discussed in terms of the recommended allowances appropriate for the needs of the majority of individuals. Deficiency can result in xerophthalmia and permanent blindness and in increased mortality rates among children. Toxicity has been associated with the overconsumption of vitamin A supplements. Acute hypervitaminosis A may occur after ingestion of greater than or equal to 500,000 IU (over 100 times the RDA) by adults or proportionately less by children. Symptoms are usually reversible on cessation of overdosing. Factors influencing chronic hypervitaminosis A include dosing regimen, physical form of the vitamin, general health status, dietary factors such as ethanol and protein intake, and interactions with vitamins C, D, E, and K. Both excess and deficiency of vitamin A in pregnant animals was shown to be	6	Non-systematic review

				teratogenic. In humans, congenital malformations associated with maternal over-use of high doses of vitamin A were reported but no cause-and-effect relationship has been established. Deficiency of the vitamin during pregnancy has also been associated with congenital abnormalities. Reported incidences of vitamin A toxicity are rare and have averaged fewer than 10 cases per year from 1976 to 1987.		
Dietary Reference Intakes for Vitamin A, Vitamin K, Arsenic, Boron, Chromium, Copper, Iodine, Iron, Manganese, Molybdenum, Nickel, Silicon, Vanadium, and Zinc	Adverse Effects in Infants and Children	The more commonly recognized signs and symptoms include skeletal abnormalities, bone tenderness and pain, increased intracranial pressure, desquamation, brittle nails, mouth fissures, alopecia, fever, headache, lethargy, irritability, weight loss, vomiting, and hepatomegaly	Fatal hypervitaminosis A in a neonate [171]	Although hypervitaminosis A is not uncommon, fatal cases are rare. We describe a neonate who died after having ingested more than 60 times the suggested dose of vitamin A per day, for 11 days. His hospital course was marked by hypercalcemia, hyperphosphatemia, a bleeding disorder, and pulmonary insufficiency. An autopsy showed extensive calcifications of the alveolar septa and bronchioles. Metastatic calcifications were also present in the kidneys, stomach, soft tissue, and skin. The skeleton showed prominent alteration of the endochondral bone formation. There was also evidence of accelerated resorption of bone, which is presumably responsible for the development of hypercalcemia and metastatic calcification.	5	Observational
Dietary Reference Intakes for Vitamin A, Vitamin K, Arsenic, Boron, Chromium, Copper, Iodine, Iron, Manganese, Molybdenum, Nickel, Silicon, Vanadium, and Zinc	Adverse Effects in Infants and Children	Carpenter and coworkers (1987) described two boys who developed hypervitaminosis A by age 2 years for one and by age 6 years for the other. Both were given chicken liver that supplied about 690 µg/day of vitamin A and various supplements that supplied another	Severe hypervitaminosis A in siblings: Evidence of variable tolerance to retinol intake [172]	A 2-year-old boy had signs and symptoms of chronic hypervitaminosis A. A course of increasing severity led to eventual death. A younger brother later had similar clinical features. Chicken liver spread containing up to 420 IU/g vitamin A was the likely source of intoxication. Markedly elevated circulating retinyl ester levels have persisted in the surviving sibling for 3 subsequent years despite severe restriction of vitamin A intake. A therapeutic trial of the carbohydrate-derived complexing agent 2-hydroxypropyl-beta-cyclodextrin was initiated. Circulating retinyl esters transiently increased during the infusion (from 407 to 4791 micrograms/dL), and urinary total vitamin A excretion, undetectable before infusion, increased to 23 micrograms/dL after infusion. The frequency of hypervitaminotic episodes has decreased somewhat in the 2 years since the infusion, probably related to dietary vitamin A	5	Observational

		135 to 750µg/day. An older sister who had been treated similarly remained completely healthy.		restriction. The occurrence of this syndrome in two brothers, while a sister ingesting the same diet remains completely healthy, suggests an inherited variance in tolerance to vitamin A intake.		
Dietary Reference Intakes for Vitamin A, Vitamin K, Arsenic, Boron, Chromium, Copper, Iodine, Iron, Manganese, Molybdenum, Nickel, Silicon, Vanadium, and Zinc	Adverse Effects in Infants and Children	Cases of Subchronic and Chronic, Low-Dose Vitamin A Toxicity in Infants	Chronic vitamin A intoxication in infants fed chicken liver [173]	Twin female infants were fed 120 gm of chicken liver homogenate daily for four months. They developed irritability, vomiting, and bulging anterior fontanelles. Computed tomograms of the brain revealed enlarged ventricles in both infants and dilated subarachnoid spaces in one. Plasma vitamin A concentrations were elevated. After all sources of vitamin A intake were stopped, the infants recovered without sequelae. The chicken liver homogenate contained 36,000 IU of vitamin A per 120 gm. Since infants often receive 4,000 units of vitamin A daily from fortified milk and vitamin supplements, they probably cannot be fed 60 gm of chicken liver safely more often than once weekly.	5	Observational
Dietary Reference Intakes for Vitamin A, Vitamin K, Arsenic, Boron, Chromium, Copper, Iodine, Iron, Manganese, Molybdenum, Nickel, Silicon, Vanadium, and Zinc	Adverse Effects in Infants and Children	Cases of Subchronic and Chronic, Low-Dose Vitamin A Toxicity in Infants	Hypervitaminosis A: Report of an unusual case with marked craniotabes [174]	No abstract available.	5	Observational
Dietary Reference Intakes for Vitamin A, Vitamin K, Arsenic, Boron, Chromium, Copper, Iodine, Iron, Manganese, Molybdenum, Nickel, Silicon, Vanadium, and Zinc	Adverse Effects in Infants and Children	Cases of Subchronic and Chronic, Low-Dose Vitamin A Toxicity in Infants	Acute and chronic hypervitaminosis in a 4-month-old infant [175]	No abstract available.	5	Observational
Dietary Reference Intakes for Vitamin A, Vitamin K,	Adverse Effects in Infants and Children	Cases of Subchronic and Chronic, Low-	Hypervitaminosis A: A case report [176]	No abstract available.	5	Observational

Arsenic, Boron, Chromium, Copper, Iodine, Iron, Manganese, Molybdenum, Nickel, Silicon, Vanadium, and Zinc		Dose Vitamin A Toxicity in Infants				
Dietary Reference Intakes for Vitamin A, Vitamin K, Arsenic, Boron, Chromium, Copper, Iodine, Iron, Manganese, Molybdenum, Nickel, Silicon, Vanadium, and Zinc	Adverse Effects in Infants and Children	Cases of Subchronic and Chronic, Low-Dose Vitamin A Toxicity in Infants	Chronic hypervitaminosis A with intracranial hypertension and low cerebrospinal fluid concentration of protein. Two illustrative cases [177]	An unusually high dietary intake can lead to vitamin A intoxication. These two children had clinical and radiologic evidences of increased intracranial pressure and low cerebrospinal fluid protein concentrations.	5	Observational
Dietary Reference Values for Food Energy and Nutrients for the United Kingdom (1991)	Requirements	Children are more sensitive, and the Panel agreed with the recommendations of others that regular daily intakes should not exceed 900µg in infants; 1,800µg between 1 and 3 years of age; 3,000µg from 4 to 6 years old; 4,500µg from 6 to 12 years old; or 6,000µg for adolescents	The Safe Use of Vitamin A [178]	No abstract available.		Published guidelines

Supplementary References

1. Tanumihardjo SA, Russell RM, Stephensen CB, Gannon BM, Craft NE, Haskell MJ, et al. Biomarkers of Nutrition for Development (BOND)—Vitamin A Review. The Journal of Nutrition. 2016;146(9):1816S-48S. 10.3945/jn.115.229708
2. EFSA NDA Panel (EFSA Panel on Dietetic Products NaA. Scientific opinion on Dietary Reference Values for vitamin A. EFSA Journal,. 2015;13(3). 10.2903/j.efsa.2015.4028
3. WHO/FAO (World Health Organization/Food and Agriculture Organization of the United Nations). Vitamin and mineral requirements in human nutrition: report of a Joint FAO/WHO Expert Consultation 21-30 September 1998. Bangkok: Thailand; 2004.
4. IOM (Institute of Medicine). Dietary Reference Intakes for vitamin A, vitamin K, arsenic, boron, chromium, copper, iodine, iron, manganese, molybdenum, nickel, silicon, vanadium, and zinc. Washington DC: National Academy Press; 2001.
5. DH (Department of Health). Dietary Reference Values for food energy and nutrients for the United Kingdom. Report of the Panel on Dietary Reference Values of the Committee on Medical Aspects of Food Policy. London, UK: HMSO; 1991.
6. Katz J, West KP, Jr., Khatry SK, Thapa MD, LeClerq SC, Pradhan EK, et al. Impact of vitamin A supplementation on prevalence and incidence of xerophthalmia in Nepal. Invest Ophthalmol Vis Sci. 1995;36(13):2577-83.
7. Chase HP, Kumar V, Dodds JM, Sauberlich HE, Hunter RM, Burton RS, et al. Nutritional Status of Preschool Mexican-American Migrant Farm Children. American Journal of Diseases of Children. 1971;122(4):316-24. 10.1001/archpedi.1971.02110040100008
8. Mayo-Wilson E, Imdad A, Herzer K, Yakoob MY, Bhutta ZA. Vitamin A supplements for preventing mortality, illness, and blindness in children aged under 5: systematic review and meta-analysis. BMJ. 2011;343:d5094. 10.1136/bmj.d5094
9. Scientific Committee on Food. Opinion on the Tolerable Upper Intake Level of preformed vitamin A (retinol and retinyl esters). 2002.
10. Grotto I, Mimouni M, Gdalevich M, Mimouni D. Vitamin A supplementation and childhood morbidity from diarrhea and respiratory infections: a meta-analysis. J Pediatr. 2003;142(3):297-304. 10.1067/mpd.2003.116
11. Chen H, Zhuo Q, Yuan W, Wang J, Wu T. Vitamin A for preventing acute lower respiratory tract infections in children up to seven years of age. Cochrane Database Syst Rev. 2008(1):Cd006090. 10.1002/14651858.CD006090.pub2
12. Reddy V, Sivakumar B. Studies on vitamin A absorption in children. Indian Pediatr. 1972;9(6):307-10.
13. Sivakumar B, Reddy V. Absorption of labelled vitamin A in children during infection. British Journal of Nutrition. 1972;27(2):299-304. 10.1079/BJN19720094
14. Kusin JA, Reddy V, Sivakumar B. Vitamin E supplements and the absorption of a massive dose of vitamin A. The American journal of clinical nutrition. 1974;27(8):774-6. 10.1093/ajcn/27.8.774
15. Olson JA. The prevention of childhood blindness by the administration of massive doses of vitamin A. Isr J Med Sci. 1972;8(8):1199-206.
16. Haskell MJ, Lembcke JL, Salazar M, Green MH, Peerson JM, Brown KH. Population-based plasma kinetics of an oral dose of [2H4]retinyl acetate among preschool-aged, Peruvian children. Am J Clin Nutr. 2003;77(3):681-6. 10.1093/ajcn/77.3.681
17. Stoltzfus RJ, Underwood BA. Breast-milk vitamin A as an indicator of the vitamin A status of women and infants. Bulletin of the World Health Organization. 1995;73(5):703-11.
18. Macias C, Schweigert FJ. Changes in the concentration of carotenoids, vitamin A, alpha-tocopherol and total lipids in human milk throughout early lactation. Ann Nutr Metab. 2001;45(2):82-5. 10.1159/000046711
19. Underwood BA. Maternal vitamin A status and its importance in infancy and early childhood. The American journal of clinical nutrition. 1994;59(2 Suppl):517S-22S; discussion 22S-24S. 10.1093/ajcn/59.2.517S
20. Canfield LM, Clandinin MT, Davies DP, Fernandez MC, Jackson J, Hawkes J, et al. Multinational study of major breast milk carotenoids of healthy mothers. Eur J Nutr. 2003;42(3):133-41. 10.1007/s00394-003-0403-9
21. Schweigert FJ, Bathe K, Chen F, Büscher U, Dudenhausen JW. Effect of the stage of lactation in humans on carotenoid levels in milk, blood plasma and plasma lipoprotein fractions. European journal of nutrition. 2004;43(1):39-44.

22. Tokuşoğlu O, Tansuğ N, Akşit S, Dinç G, Kasirga E, Ozcan C. Retinol and alpha-tocopherol concentrations in breast milk of Turkish lactating mothers under different socio-economic status. Int J Food Sci Nutr. 2008;59(2):166-74. 10.1080/02699200701539171
23. Duda G, Nogala-KaBucka M, Karwowska W, Kupczyk B, Lampart-Szczapa E. Influence of the lactating women diet on the concentration of the lipophilic vitamins in human milk. Pakistan Journal of Nutrition. 2009;8:629-34.
24. Kašparová M, Plíšek J, Solichová D, Krčmová L, Kučerová B, Hronek M, et al. Rapid sample preparation procedure for determination of retinol and α-tocopherol in human breast milk. Talanta. 2012;93:147-52. 10.1016/j.talanta.2012.01.065
25. Butte NF, Lopez-Alarcon MG, Garza C, Expert Consultation on the Optimal Duration of Exclusive B. Nutrient adequacy of exclusive breastfeeding for the term infant during the first six months of life / Nancy F. Butte, Mardia G. Lopez-Alarcon, Cutberto Garza. Geneva: World Health Organization; 2002.
26. Bloem MW, Wedel M, Egger RJ, Speek AJ, Schrijver J, Saowakontha S, et al. Iron metabolism and vitamin A deficiency in children in northeast Thailand. The American journal of clinical nutrition. 1989;50(2):332-8. 10.1093/ajcn/50.2.332
27. Mwanri L, Worsley A, Ryan P, Masika J. Supplemental vitamin A improves anemia and growth in anemic school children in Tanzania. The Journal of nutrition. 2000;130(11):2691-6. 10.1093/jn/130.11.2691
28. Al-Mekhlafi HM, Al-Zabedi EM, Al-Maktari MT, Atroosh WM, Al-Delaimy AK, Moktar N, et al. Effects of vitamin A supplementation on iron status indices and iron deficiency anaemia: a randomized controlled trial. Nutrients. 2013;6(1):190-206. 10.3390/nu6010190
29. van Lieshout M, West CE, Muhilal, Permaesih D, Wang Y, Xu X, et al. Bioefficacy of beta-carotene dissolved in oil studied in children in Indonesia. The American journal of clinical nutrition. 2001;73(5):949-58. 10.1093/ajcn/73.5.949
30. Tang G, Hu Y, Yin SA, Wang Y, Dallal GE, Grusak MA, et al. β-Carotene in Golden Rice is as good as β-carotene in oil at providing vitamin A to children. Am J Clin Nutr. 2012;96(3):658-64. 10.3945/ajcn.111.030775
31. Hoppner K, Phillips WE, Erdody P, Murray TK, Perrin DE. Vitamin A reserves of Canadians. Can Med Assoc J. 1969;101(12):84-6.
32. Underwood BA, Siegel H, Weisell RC, Dolinski M. Liver Stores of Vitamin A in a Normal Population Dying Suddenly or Rapidly from Unnatural Causes in New York City. The American journal of clinical nutrition. 1970;23(8):1037-42. 10.1093/ajcn/23.8.1037
33. Mitchell GV, Young M, Seward CR. Vitamin A and carotene levels of a selected population in metropolitan Washington, D. C. The American journal of clinical nutrition. 1973;26(9):992-7. 10.1093/ajcn/26.9.992
34. Money D, DFL M. Vitamin E, selenium, iron and vitamin A content of livers from sudden infant death syndrome cases and control children: interrelationships and possible significance. 1978.
35. Huque T. A survey of human liver reserves of retinol in London. Brit J Nutr. 1982;47(2):165-72.
36. Schindler R, Friedrich DH, Krämer M, Wacker HH, Feldheim W. Size and composition of liver vitamin A reserves of human beings who died of various causes. Int J Vitam Nutr Res. 1988;58(2):146-54.
37. Suthutvoravoot S, Olson JA. Plasma and liver concentrations of vitamin A in a normal population of urban Thai. The American journal of clinical nutrition. 1974;27(8):883-91. 10.1093/ajcn/27.8.883
38. Olson JA. Liver vitamin A reserves of neonates, preschool children and adults dying of various causes in Salvador, Brazil. Arch Latinoam Nutr. 1979;29(4):521-45.
39. Flores H, de Araújo CR. Liver levels of retinol in unselected necropsy specimens: a prevalence survey of vitamin A deficiency in Recife, Brazil. The American journal of clinical nutrition. 1984;40(1):146-52. 10.1093/ajcn/40.1.146
40. World Health Organization. Indicators for assessing vitamin A deficiency and their application in monitoring and evaluating intervention programmes. WHO/NUT/96.10 Geneva, Switzerland: WHO; 1996.
41. World Health Organization. Serum retinol concentrations for determining the prevalence of vitamin A deficiency in populations. WHO/NMH/NHD/MNM/11.3. Geneva, Switzerland: WHO; 2011.
42. Pilch S. Analysis of Vitamin A Data from the Health and Nutrition Examination Surveys. The Journal of nutrition. 1987;117(4):636-40. 10.1093/jn/117.4.636
43. Flores H, Azevedo MN, Campos FA, Barreto-Lins MC, Cavalcanti AA, Salzano AC, et al. Serum vitamin A distribution curve for children aged 2-6 y known to have adequate vitamin A status: a reference population. The American journal of clinical nutrition. 1991;54(4):707-11. 10.1093/ajcn/54.4.707
44. Filteau SM, Morris SS, Abbott RA, Tomkins AM, Kirkwood BR, Arthur P, et al. Influence of morbidity on serum retinol of children in a community-based study in northern Ghana. The American journal of clinical nutrition. 1993;58(2):192-7. 10.1093/ajcn/58.2.192

45. Helldán A, Raulio S, Kosola M, Tapanainen H, Ovaskainen M-L, Virtanen S. Finravinto 2012-tutkimus: The national FINDIET 2012 survey. 2013.
46. Kersting M, Clausen K, Heseker H. Ernährungsphysiologische Auswertung einer repräsentativen Verzehrsstudie bei Säuglingen und Kleinkindern VELS mit dem Instrumentarium der DONALD Studie. Forschungsinstitut für Kinderernährung, Dortmund, Germany. 2003.
47. Sette S, Le Donne C, Piccinelli R, Arcella D, Turrini A, Leclercq C, et al. The third Italian national food consumption survey, INRAN-SCAI 2005–06–part 1: nutrient intakes in Italy. Nutrition, Metabolism and Cardiovascular Diseases. 2011;21(12):922-32.
48. Bates B, Lennox A, Prentice A, Bates C, Swan G. National diet and nutrition survey. Headline results from years 1, 2 and 3 (combined) of the rolling programme (2008/2009–2010/11). Department of Health London; 2012.
49. Mensink G, Heseker H, Richter A, Stahl A, Vohmann C, Fischer J, et al. Ernährungsstudie als KiGGS-Modul (EsKiMo). 2007.
50. AFSSA. Étude Individuelle Nationale des Consommations Alimentaires 2 (INCA 2)(2006–2007). Individual and National Study on Food Consumption. 2009;2:228.
51. van Rossum CT, Fransen HP, Verkaik-Kloosterman Ja, Buurma-Rethans EJ, Ocke MC. Dutch National Food Consumption Survey 2007-2010: Diet of children and adults aged 7 to 69 years. 2011.
52. Fawzi WW, Herrera MG, Nestel P. Vitamin A supplementation in malnourished Sudanese children. Int J Vitam Nutr Res. 1992;62(4):344.
53. Ghana VAST Study Team. Vitamin A supplementation in northern Ghana: effects on clinic attendances, hospital admissions, and child mortality. . Lancet. 1993;342(8862):7-12.
54. Beaton G, Martorell R, Aronson K, Edmonston B, McCabe G, Ross A, et al. Effectiveness of Vitamin A Supplementation in the Control of Young Child Morbidity and Mortality in Developing Countries. United Nations Administrative Committee on Coordination, Sub-committee on Nutrition Stateof-the-art Series. Nutrition policy discussion paper. 1993(13).
55. Glasziou PP, Mackerras DE. Vitamin A supplementation in infectious diseases: a meta-analysis. Bmj. 1993;306(6874):366-70. 10.1136/bmj.306.6874.366
56. Brown N, Roberts C. Vitamin A for acute respiratory infection in developing countries: a meta-analysis. Acta Paediatr. 2004;93(11):1437-42. 10.1080/08035250410022143
57. Ni J, Wei J, Wu T. Vitamin A for non-measles pneumonia in children. Cochrane Database Syst Rev. 2005;2005(3):Cd003700. 10.1002/14651858.CD003700.pub2
58. Imdad A, Yakoob MY, Sudfeld C, Haider BA, Black RE, Bhutta ZA. Impact of vitamin A supplementation on infant and childhood mortality. BMC Public Health. 2011;11 Suppl 3(Suppl 3):S20. 10.1186/1471-2458-11-s3-s20
59. McLaren DS, Kraemer K. Mortality and morbidity, especially in relation to infections. World Rev Nutr Diet. 2012;103:76-92. 10.1159/000170976
60. Nurmatov U, Devereux G, Sheikh A. Nutrients and foods for the primary prevention of asthma and allergy: systematic review and meta-analysis. J Allergy Clin Immunol. 2011;127(3):724-33.e1-30. 10.1016/j.jaci.2010.11.001
61. Ribaya-Mercado JD, Solon FS, Solon MA, Cabal-Barza MA, Perfecto CS, Tang G, et al. Bioconversion of plant carotenoids to vitamin A in Filipino school-aged children varies inversely with vitamin A status. The American journal of clinical nutrition. 2000;72(2):455-65.
62. Ribaya-Mercado JD, Maramag CC, Tengco LW, Dolnikowski GG, Blumberg JB, Solon FS. Carotene-rich plant foods ingested with minimal dietary fat enhance the total-body vitamin A pool size in Filipino schoolchildren as assessed by stable-isotope-dilution methodology. The American journal of clinical nutrition. 2007;85(4):1041-9. 10.1093/ajcn/85.4.1041
63. Van Loo-Bouwman CA, Naber THJ, Schaafsma G. A review of vitamin A equivalency of β-carotene in various food matrices for human consumption. Brit J Nutr. 2014;111(12):2153-66. 10.1017/S0007114514000166
64. Sommer A. Vitamin A deficiency and its consequences: a field guide to detection and control: World Health Organization; 1995.
65. Sommer A, Emran N, Tjakrasudjatma S. Clinical characteristics of vitamin A responsive and nonresponsive Bitot's spots. American journal of ophthalmology. 1980;90(2):160-71.
66. Newman V. Vitamin A and breast-feeding: a comparison of data from developed and developing countries. Food and Nutrition Bulletin. 1994;15(2):1-16.
67. Sellen D. Physical Status: The Use and Interpretation of Anthropometry. Report of a WHO Expert Committee. WHO Technical Report Series No. 854. Pp. 452.(WHO, Geneva, 1995.) Swiss Fr 71.00. Journal of Biosocial Science. 1998;30(1):135-44.

68. Sinha D, Bang F. Seasonal variation in signs of vitamin-A deficiency in rural West Bengal children. The Lancet. 1973;302(7823):228-31.
69. Mele L, West KP, Jr., Kusdiono, Pandji A, Nendrawati H, Tilden RL, et al. Nutritional and household risk factors for xerophthalmia in Aceh, Indonesia: a case-control study. The Aceh Study Group. The American journal of clinical nutrition. 1991;53(6):1460-5. 10.1093/ajcn/53.6.1460
70. Tarwotjo I, Sommer A, Soegiharto T, Susanto D, Muhilal. Dietary practices and xerophthalmia among Indonesian children. The American journal of clinical nutrition. 1982;35(3):574-81. 10.1093/ajcn/35.3.574
71. Zeitlin MF, Megawangi R, Kramer EM, Armstrong HC. Mothers' and children's intakes of vitamin A in rural Bangladesh. The American journal of clinical nutrition. 1992;56(1):136-47. 10.1093/ajcn/56.1.136
72. Ross DA, Dollimore N, Smith P, Kirkwood B, Arthur P, Morris S, et al. Vitamin A supplementation in northern Ghana: effects on clinic attendances, hospital admissions, and child mortality. The Lancet. 1993;342(8862):7-12.
73. Barreto ML, Santos LM, Assis AM, Araújo MP, Farenzena GG, Santos PA, et al. Effect of vitamin A supplementation on diarrhoea and acute lower-respiratory-tract infections in young children in Brazil. Lancet. 1994;344(8917):228-31. 10.1016/s0140-6736(94)92998-x
74. Bhandari N, Bhan MK, Sazawal S. Impact of massive dose of vitamin A given to preschool children with acute diarrhoea on subsequent respiratory and diarrhoeal morbidity. Bmj. 1994;309(6966):1404-7. 10.1136/bmj.309.6966.1404
75. Fawzi WW, Chalmers TC, Herrera MG, Mosteller F. Vitamin A supplementation and child mortality. A meta-analysis. JAMA. 1993;269(7):898-903.
76. Menon K, Vijayaraghavan K. Sequelae of severe xerophthalmia--a follow-up study. The American journal of clinical nutrition. 1980;33(2):218-20. 10.1093/ajcn/33.2.218
77. Hussey GD, Klein M. A randomized, controlled trial of vitamin A in children with severe measles. N Engl J Med. 1990;323(3):160-4. 10.1056/nejm199007193230304
78. The Vitamin A and Pneumonia Working Group. Potential interventions for the prevention of childhood pneumonia in developing countries: a meta-analysis of data from field trials to assess the impact of vitamin A supplementation on pneumonia morbidity and mortality. The Vitamin A and Pneumonia Working Group. Bull World Health Organ. 1995;73(5):609-19.
79. Coutsoudis A, Broughton M, Coovadia HM. Vitamin A supplementation reduces measles morbidity in young African children: a randomized, placebo-controlled, double-blind trial. The American journal of clinical nutrition. 1991;54(5):890-5. 10.1093/ajcn/54.5.890
80. Feachem RG. Vitamin A deficiency and diarrhoea: a review of interrelationships and their implications for the control of xerophthalmia and diarrhoea. Tropical diseases bulletin 1987: 84 (3): R1-R16. 1987.
81. Campos FA, Flores H, Underwood BA. Effect of an infection on vitamin A status of children as measured by the relative dose response (RDR). The American journal of clinical nutrition. 1987;46(1):91-4. 10.1093/ajcn/46.1.91
82. Foster A, Yorston D. Corneal ulceration in Tanzanian children: relationship between measles and vitamin A deficiency. Trans R Soc Trop Med Hyg. 1992;86(4):454-5. 10.1016/0035-9203(92)90268-h
83. ARROYAVE G, WILSON D, MÉNDEZ J, BÉHAR M, SCRIMSHAW NS. Serum and Liver Vitamin A and Lipids in Children with Severe Protein Malnutrition. The American journal of clinical nutrition. 1961;9(2):180-5. 10.1093/ajcn/9.2.180
84. Tang G, Gu X, Hu S, Xu Q, Qin J, Dolnikowski GG, et al. Green and yellow vegetables can maintain body stores of vitamin A in Chinese children. The American journal of clinical nutrition. 1999;70(6):1069-76. 10.1093/ajcn/70.6.1069
85. Moshfegh A, Tippett K, Borrud L, Perloff B. Food and nutrient intakes by individuals in the United States, by sex and age, 1994–96. The Third Nationwide Food Consumption Survey. 1998.
86. US Department of Health Human Services. Third National Health and Nutrition Examination Survey (NHANES III), 1988-94. NHANES III examination data file documentation catalog. 1996(76200).
87. Yin S, Oin J, Gu S, Xu O, Zhao S, Tang G, et al., editors. Green and yellow vegetables rich in provitamin A carotenoids can sustain vitamin A status of children. FASEB JOURNAL; 1998: Federation Amer Soc Exp Biol 9650 Rockville Pike, Bethesda, Md 20814-3998 USA.
88. Jalal F, Nesheim MC, Agus Z, Sanjur D, Habicht JP. Serum retinol concentrations in children are affected by food sources of beta-carotene, fat intake, and anthelmintic drug treatment. The American journal of clinical nutrition. 1998;68(3):623-9. 10.1093/ajcn/68.3.623
89. Flores H, Campos F, Araujo CR, Underwood BA. Assessment of marginal vitamin A deficiency in Brazilian children using the relative dose response procedure. The American journal of clinical nutrition. 1984;40(6):1281-9.

90. UNICEF. Complementary feeding of young children in developing countries: a review of current scientific knowledge: WHO; 1998.
91. Rahmathullah L, Underwood BA, Thulasiraj RD, Milton RC, Ramaswamy K, Rahmathullah R, et al. Reduced mortality among children in southern India receiving a small weekly dose of vitamin A. New England journal of medicine. 1990;323(14):929-35.
92. Karr M, Mira M, Causer J, Earl J, Alperstein G, Wood F, et al. Age-specific reference intervals for plasma vitamins A, E and beta-carotene and for serum zinc, retinol-binding protein and prealbumin for Sydney children aged 9-62 months. Int J Vitam Nutr Res. 1997;67(6):432-6.
93. de Pee S, West CE, Muhilal, Karyadi D, Hautvast JG. Lack of improvement in vitamin A status with increased consumption of dark-green leafy vegetables. Lancet. 1995;346(8967):75-81. 10.1016/s0140-6736(95)92111-7
94. de Pee S, West CE, Permaesih D, Martuti S, Muhilal, Hautvast JG. Orange fruit is more effective than are dark-green, leafy vegetables in increasing serum concentrations of retinol and beta-carotene in schoolchildren in Indonesia. Am J Clin Nutr. 1998;68(5):1058-67. 10.1093/ajcn/68.5.1058
95. Sommer A, WEST K. Vitamin A deficiency health, survival y vision. OxforUniversity, Press. Inc New Andork. 1996.
96. Sommer A. Nutritional blindness. Xerophthalmia and keratomalacia: New York, USA; Oxford University Press.; 1982.
97. Sommer A, Katz J, Tarwotjo I. Increased risk of respiratory disease and diarrhea in children with preexisting mild vitamin A deficiency. The American journal of clinical nutrition. 1984;40(5):1090-5.
98. Sommer A, Hussaini G, Tarwotjo I, Susanto D. Increased mortality in children with mild vitamin A deficiency. The Lancet. 1983;322(8350):585-8.
99. Barclay AJ, Foster A, Sommer A. Vitamin A supplements and mortality related to measles: a randomised clinical trial. Br Med J (Clin Res Ed). 1987;294(6567):294-6. 10.1136/bmj.294.6567.294
100. Donnen P, Dramaix M, Brasseur D, Bitwe R, Vertongen F, Hennart P. Randomized placebo-controlled clinical trial of the effect of a single high dose or daily low doses of vitamin A on the morbidity of hospitalized, malnourished children. The American journal of clinical nutrition. 1998;68(6):1254-60. 10.1093/ajcn/68.6.1254
101. Shankar AH, Genton B, Semba RD, Baisor M, Paino J, Tamja S, et al. Effect of vitamin A supplementation on morbidity due to Plasmodium falciparum in young children in Papua New Guinea: a randomised trial. Lancet. 1999;354(9174):203-9. 10.1016/s0140-6736(98)08293-2
102. Humphrey JH, Agoestina T, Wu L, Usman A, Nurachim M, Subardja D, et al. Impact of neonatal vitamin A supplementation on infant morbidity and mortality. J Pediatr. 1996;128(4):489-96. 10.1016/s0022-3476(96)70359-1
103. Muhilal, Permeisih D, Idjradinata YR, Muherdiyantiningsih, Karyadi D. Vitamin A-fortified monosodium glutamate and health, growth, and survival of children: a controlled field trial. The American journal of clinical nutrition. 1988;48(5):1271-6. 10.1093/ajcn/48.5.1271
104. Sommer A, Tarwotjo I, Djunaedi E, West KP, Jr., Loeden AA, Tilden R, et al. Impact of vitamin A supplementation on childhood mortality. A randomised controlled community trial. Lancet. 1986;1(8491):1169-73. 10.1016/s0140-6736(86)91157-8
105. West Jr KP, Katz J, LeClerq SC, Pradhan EK, Tielsch JM, Sommer A, et al. Efficacy of vitamin A in reducing preschool child mortality in Nepal. The Lancet. 1991;338(8759):67-71.
106. World Health Organization. Vitamin A supplements: a guide to their use in the treatment of vitamin A deficiency and xerophthalmia. 1997.
107. Committee on Infectious Diseases. Vitamin A treatment of measles. Pediatrics. 1993;91(5):1014-5.
108. Arthur P, Kirkwood B, Ross D, Morris S, Gyapong J, Tomkins A, et al. Impact of vitamin A supplementation on childhood morbidity in northern Ghana. Lancet. 1992;339(8789):361-2. 10.1016/0140-6736(92)91677-z
109. Bloem MW, Wedel M, Egger RJ, Speek AJ, Schrijver J, Saowakontha S, et al. Mild vitamin A deficiency and risk of respiratory tract diseases and diarrhea in preschool and school children in northeastern Thailand. American journal of epidemiology. 1990;131(2):332-9. 10.1093/oxfordjournals.aje.a115502
110. Lloyd-Puryear MA, Mahoney J, Humphrey JH, Mahoney F, Siren N, Moorman C, et al. Vitamin a deficiency in micronesia: A statewide survey in chuuk. Nutrition Research. 1991;11(10):1101-10. https://doi.org/10.1016/S0271-5317(05)80688-8
111. Salazar-Lindo E, Salazar M, Alvarez JO. Association of diarrhea and low serum retinol in Peruvian children. The American journal of clinical nutrition. 1993;58(1):110-3. 10.1093/ajcn/58.1.110
112. Congdon N, Sommer A, Severns M, Humphrey J, Friedman D, Clement L, et al. Pupillary and visual thresholds in young children as an index of population vitamin A status. The American journal of clinical nutrition. 1995;61(5):1076-82. 10.1093/ajcn/61.4.1076

113. Sanchez AM, Congdon NG, Sommer A, Rahmathullah L, Venkataswamy PG, Chandravathi PS, et al. Pupillary threshold as an index of population vitamin A status among children in India. The American journal of clinical nutrition. 1997;65(1):61-6. 10.1093/ajcn/65.1.61
114. Devadas RR, Premakumari S, Subramaniam G. Biological availability of beta carotene from fresh and dried green leafy vegetables on preschool children. Indian journal of nutrition and dietetics. 1978.
115. Filteau SM, Morris SS, Raynes JG, Arthur P, Ross DA, Kirkwood BR, et al. Vitamin A supplementation, morbidity, and serum acute-phase proteins in young Ghanaian children. The American journal of clinical nutrition. 1995;62(2):434-8. 10.1093/ajcn/62.2.434
116. Looker AC, Johnson CL, Woteki CE, Yetley EA, Underwood BA. Ethnic and racial differences in serum vitamin A levels of children aged 4-11 years. The American journal of clinical nutrition. 1988;47(2):247-52. 10.1093/ajcn/47.2.247
117. Natadisastra G, Wittpenn JR, West KP, Jr, Muhilal, Sommer A. Impression Cytology for Detection of Vitamin A Deficiency. Archives of Ophthalmology. 1987;105(9):1224-8. 10.1001/archopht.1987.01060090082033
118. Wittpenn JR, Tseng SC, Sommer A. Detection of early xerophthalmia by impression cytology. Arch Ophthalmol. 1986;104(2):237-9. 10.1001/archopht.1986.01050140091027
119. Keenum DG, Semba RD, Wirasasmita S, Natadisastra G, Muhilal, West KP, Jr, et al. Assessment of Vitamin A Status by a Disk Applicator for Conjunctival Impression Cytology. Archives of Ophthalmology. 1990;108(10):1436-41. 10.1001/archopht.1990.01070120084034
120. Carlier C, Moulia-Pelat JP, Ceccon JF, Mourey MS, Fall M, N'Diaye M, et al. Prevalence of malnutrition and vitamin A deficiency in the Diourbel, Fatick, and Kaolack regions of Senegal: feasibility of the method of impression cytology with transfer. The American journal of clinical nutrition. 1991;53(1):66-9. 10.1093/ajcn/53.1.66
121. Semba RD, Muhilal, Scott AL, Natadisastra G, Wirasasmita S, Mele L, et al. Depressed immune response to tetanus in children with vitamin A deficiency. The Journal of nutrition. 1992;122(1):101-7. 10.1093/jn/122.1.101
122. Semba RD, Bulterys M, Munyeshuli V, Gatsinzi T, Saah A, Chao A, et al. Vitamin A deficiency and T-cell subpopulations in children with meningococcal disease. J Trop Pediatr. 1996;42(5):287-90. 10.1093/tropej/42.5.287
123. REDDY V, SRIKANTIA SG. Serum Vitamin A in Kwashiorkor. The American journal of clinical nutrition. 1966;18(2):105-9. 10.1093/ajcn/18.2.105
124. Roels OA, Djaeni S, Trout ME, Lauw TG, Heath A, Poey SH, et al. The effect of protein and fat supplements on vitamin A-deficient Indonesian children. The American journal of clinical nutrition. 1963;12:380-7. 10.1093/ajcn/12.5.380
125. Figueira F, Mendonça S, Rocha J, Azevedo M, Bunce GE, Reynolds JW. Absorption of vitamin A by infants receiving fat-free or fat-containing dried skim milk formulas. The American journal of clinical nutrition. 1969;22(5):588-93. 10.1093/ajcn/22.5.588
126. Roels OA, Trout M, Dujacquier R. Carotene Balances on Boys in Ruanda where Vitamin A Deficiency is Prevalent. The Journal of nutrition. 1958;65(1):115-27. 10.1093/jn/65.1.115
127. Jayarajan P, Reddy V, Mohanram M. Effect of dietary fat on absorption of beta carotene from green leafy vegetables in children. Indian J Med Res. 1980;71:53-6.
128. Mahalanabis D, Simpson TW, Chakraborty ML, Ganguli C, Bhattacharjee AK, Mukherjee KL. Malabsorption of water miscible vitamin A in children with giardiasis and ascariasis. The American journal of clinical nutrition. 1979;32(2):313-8. 10.1093/ajcn/32.2.313
129. Sivakumar B, Reddy V. Absorption of vitamin A in children with ascariasis. J Trop Med Hyg. 1975;78(5):114-5.
130. Maxwell JD, Murray D, Ferguson A, Calder E. Ascaris lumbricoides infectation associated with jejunal mucosal abnormalities. Scott Med J. 1968;13(8):280-1. 10.1177/003693306801300806
131. Wolde-Gebriel Z, West CE, Gebru H, Tadesse AS, Fisseha T, Gabre P, et al. Interrelationship between vitamin A, iodine and iron status in schoolchildren in Shoa Region, central Ethiopia. The British journal of nutrition. 1993;70(2):593-607. 10.1079/bjn19930151
132. Shingwekar AG, Mohanram M, Reddy V. Effect of zinc supplementation on plasma levels of vitamin A and retinol-binding protein in malnourished children. Clinica chimica acta; international journal of clinical chemistry. 1979;93(1):97-100. 10.1016/0009-8981(79)90249-3
133. Canfield LM, Giuliano AR, Neilson EM, Yap HH, Graver EJ, Cui HA, et al. beta-Carotene in breast milk and serum is increased after a single beta-carotene dose. Am J Clin Nutr. 1997;66(1):52-61. 10.1093/ajcn/66.1.52
134. Canfield LM, Giuliano AR, Neilson EM, Blashil BM, Graver EJ, Yap HH. Kinetics of the response of milk and serum beta-carotene to daily beta-carotene supplementation in healthy, lactating women. Am J Clin Nutr. 1998;67(2):276-83. 10.1093/ajcn/67.2.276

135. Alvarez JO, Salazar-Lindo E, Kohatsu J, Miranda P, Stephensen CB. Urinary excretion of retinol in children with acute diarrhea. The American journal of clinical nutrition. 1995;61(6):1273-6. 10.1093/ajcn/61.6.1273
136. Large S, Neal G, Glover J, Thanangkul O, Olson RE. The early changes in retinol-binding protein and prealbumin concentrations in plasma of protein-energy malnourished children after treatment with retinol and an improved diet. The British journal of nutrition. 1980;43(3):393-402. 10.1079/bjn19800107
137. Takyi EEK. Children's Consumption of Dark Green, Leafy Vegetables with Added Fat Enhances Serum Retinol. The Journal of nutrition. 1999;129(8):1549-54. 10.1093/jn/129.8.1549
138. Muñoz EC, Rosado JL, López P, Furr HC, Allen LH. Iron and zinc supplementation improves indicators of vitamin A status of Mexican preschoolers. The American journal of clinical nutrition. 2000;71(3):789-94. 10.1093/ajcn/71.3.789
139. D'Souza RM, D'Souza R. Vitamin A for preventing secondary infections in children with measles--a systematic review. J Trop Pediatr. 2002;48(2):72-7. 10.1093/tropej/48.2.72
140. Humphrey J, West Jr K, Sommer A. Vitamin A deficiency and attributable mortality among under-5-year-olds. Bulletin of the World Health Organization. 1992;70(2):225.
141. Commission Directive 91/321/EEC of 14 May 1991 on infant formulae and follow-on formulae. Official Journal of the European Commission 1991;175(04/07):0035-49.
142. National Research Council. Recommended dietary allowances. 1989.
143. CSAH. Reports of the scientific committee for food, 31st series, 20 April 1994. : Office of the publications of the EU, ; 1994.
144. Organization WH. Requirements of Vitamin A, Iron, Folate, and Vitamin B12: Report of a Joint FAO/WHO Expert Consultation: Food & Agriculture Org.; 1988.
145. Reddy V. Observations on vitamin-A requirement. Indian J Med Res. 1971;59(6 Suppl):34-7.
146. Belavady B, Gopalan C. Chemical composition of human milk in poor Indian women. Indian J Med Res. 1959;47(2):234-45.
147. Butte NF, Calloway DH. Evaluation of lactational performance of Navajo women. Am J Clin Nutr. 1981;34(10):2210-5. 10.1093/ajcn/34.10.2210
148. D-A-CH (Deutsche Gesellschaft für Ernährung ÖGfrE, Schweizerische Gesellschaft für Ernährung). Referenzwerte für die Nährstoffzufuhr. Bonn, Germany: DGE; 2015.
149. Elmadfa I, Meyer AL. Vitamins for the first 1000 days: preparing for life. International journal for vitamin and nutrition research Internationale Zeitschrift fur Vitamin- und Ernahrungsforschung Journal international de vitaminologie et de nutrition. 2012;82(5):342-7. https://dx.doi.org/10.1024/0300-9831/a000129
150. Olson JA. Recommended dietary intakes (RDI) of vitamin A in humans. Am J Clin Nutr. 1987;45(4):704-16. 10.1093/ajcn/45.4.704
151. Clagett-Dame M, DeLuca HF. The role of vitamin A in mammalian reproduction and embryonic development. Annu Rev Nutr. 2002;22:347-81. 10.1146/annurev.nutr.22.010402.102745E
152. Ross SA, McCaffery PJ, Drager UC, De Luca LM. Retinoids in embryonal development. Physiol Rev. 2000;80(3):1021-54. 10.1152/physrev.2000.80.3.1021
153. Nau H. Teratogenicity of isotretinoin revisited: species variation and the role of all-trans-retinoic acid. J Am Acad Dermatol. 2001;45(5):S183-7. 10.1067/mjd.2001.113720
154. Buhrer C, Genzel-Boroviczeny O, Jochum F, Kauth T, Kersting M, Koletzko B, et al. Nutrition of healthy infants. Recommendations of the Nutrition Committee of the German Pediatric Society. Monatsschrift fur Kinderheilkunde. 2014;162(6):527-38. http://dx.doi.org/10.1007/s00112-014-3129-2
155. Allen LH, Haskell M. Vitamin A Requirements of Infants under Six Months of Age. Food and Nutrition Bulletin. 2001;22(3):214-34. 10.1177/156482650102200302
156. Chappell JE, Francis T, Clandinin MT. Vitamin A and E content of human milk at early stages of lactation. Early Hum Dev. 1985;11(2):157-67. 10.1016/0378-3782(85)90103-3
157. Haskell MJ, Brown KH. Maternal vitamin A nutriture and the vitamin A content of human milk. Journal of mammary gland biology and neoplasia. 1999;4(3):243-57.
158. Schweigert FJ, Bathe K, Chen F, Büscher U, Dudenhausen JW. Effect of the stage of lactation in humans on carotenoid levels in milk, blood plasma and plasma lipoprotein fractions. Eur J Nutr. 2004;43(1):39-44. 10.1007/s00394-004-0439-5
159. Neville MC, Keller R, Seacat J, Lutes V, Neifert M, Casey C, et al. Studies in human lactation: milk volumes in lactating women during the onset of lactation and full lactation. Am J Clin Nutr. 1988;48(6):1375-86. 10.1093/ajcn/48.6.1375

160. Lindquist S, Hernell O. Lipid digestion and absorption in early life: an update. Curr Opin Clin Nutr Metab Care. 2010;13(3):314-20. 10.1097/MCO.0b013e328337bbf0
161. Alcorn J, McNamara PJ. Pharmacokinetics in the newborn. Adv Drug Deliv Rev. 2003;55(5):667-86. 10.1016/s0169-409x(03)00030-9
162. Haddad S, Restieri C, Krishnan K. Characterization of age-related changes in body weight and organ weights from birth to adolescence in humans. J Toxicol Environ Health A. 2001;64(6):453-64. 10.1080/152873901753215911
163. Humphrey JH, Agoestina T, Juliana A, Septiana S, Widjaja H, Cerreto MC, et al. Neonatal vitamin A supplementation: effect on development and growth at 3 y of age. The American journal of clinical nutrition. 1998;68(1):109-17. 10.1093/ajcn/68.1.109
164. Baqui AH, de Francisco A, Arifeen SE, Siddique AK, Sack RB. Bulging fontanelle after supplementation with 25,000 IU of vitamin A in infancy using immunization contacts. Acta Paediatr. 1995;84(8):863-6. 10.1111/j.1651-2227.1995.tb13781.x
165. de Francisco A, Chakraborty J, Chowdhury HR, Yunus M, Baqui AH, Siddique AK, et al. Acute toxicity of vitamin A given with vaccines in infancy. Lancet. 1993;342(8870):526-7. 10.1016/0140-6736(93)91648-6
166. WHO/CHD Immunisation-Linked Vitamin A Supplementation Study Group. Randomised trial to assess benefits and safety of vitamin A supplementation linked to immunisation in early infancy. WHO/CHD Immunisation-Linked Vitamin A Supplementation Study Group. Lancet. 1998;352(9136):1257-63.
167. van Dillen J, de Francisco A, Overweg-Plandsoen WC. Long-term effect of vitamin A with vaccines. Lancet. 1996;347(9016):1705. 10.1016/s0140-6736(96)91541-x
168. Hathcock JN, Hattan DG, Jenkins MY, McDonald JT, Sundaresan PR, Wilkening VL. Evaluation of vitamin A toxicity. Am J Clin Nutr. 1990;52(2):183-202. 10.1093/ajcn/52.2.183
169. Persson B, Tunell R, Ekengren K. CHRONIC VITAMIN A INTOXICATION DURING THE FIRST HALF YEAR OF LIFE; DESCRIPTION OF 5 CASES. Acta Paediatr Scand. 1965;54:49-60. 10.1111/j.1651-2227.1965.tb06345.x
170. Bendich A, Langseth L. Safety of vitamin A. The American journal of clinical nutrition. 1989;49(2):358-71. 10.1093/ajcn/49.2.358
171. Bush ME, Dahms BB. Fatal hypervitaminosis A in a neonate. Arch Pathol Lab Med. 1984;108(10):838-42.
172. Carpenter TO, Pettifor JM, Russell RM, Pitha J, Mobarhan S, Ossip MS, et al. Severe hypervitaminosis A in siblings: evidence of variable tolerance to retinol intake. J Pediatr. 1987;111(4):507-12. 10.1016/s0022-3476(87)80109-9
173. Mahoney CP, Margolis MT, Knauss TA, Labbe RF. Chronic vitamin A intoxication in infants fed chicken liver. Pediatrics. 1980;65(5):893-7.
174. Arena JM, Sarazen P, Jr., Baylin GJ. Hypervitaminosis a; report of an unusual case with marked craniotabes. Pediatrics. 1951;8(6):788-93.
175. Woodard WK, Miller LJ, Legant O. Acute and chronic hypervitaminosis in a 4-month-old infant. J Pediatr. 1961;59:260-4. 10.1016/s0022-3476(61)80090-5
176. Naz JF, Edwards WM. Hypervitaminosis A. New England Journal of Medicine. 1952;246(3):87-9. 10.1056/nejm195201172460303
177. Siegel NJ, Spackman TJ. Chronic Hypervitaminosis A with Intracranial Hypertension and Low Cerebrospinal Fluid Concentration of Protein:Two Illustrative Cases. Clinical Pediatrics. 1972;11(10):580-4. 10.1177/000992287201101011
178. Bauernfeind JC. The safe use of vitamin A: a report of the International Vitamin A Consultative Group (IVACG). 1980.
